# Belief and Possibility Belief Interval-Valued N-Soft Set and Their Applications in Multi-Attribute Decision-Making Problems

**DOI:** 10.3390/e23111498

**Published:** 2021-11-13

**Authors:** Shahbaz Ali, Muneeba Kousar, Qin Xin, Dragan Pamučar, Muhammad Shazib Hameed, Rabia Fayyaz

**Affiliations:** 1 Department of Mathematics, Khwaja Fareed University of Engineering & Information Technology, Rahim Yar Khan 64200, Pakistan; shahbaz.ali@kfueit.edu.pk (S.A.); math202701001@kfueit.edu.pk (M.K.); shazib.hameed@kfueit.edu.pk (M.S.H.); 2 Faculty of Science and Technology, University of the Faroe Islands, Vestarabryggja 15, FO 100 Torshavn, Faroe Islands, Denmark; qinx@setur.fo; 3 Department of Logistics, Military Academy, University of Defence in Belgrade, 11000 Belgrade, Serbia; 4 Department of Mathematics, COMSATS University Islamabad, Islamabad 44000, Pakistan; rabia_fayyaz@comsats.edu.pk

**Keywords:** belief interval-valued soft set, belief interval-valued N-soft set, possibility belief interval-valued N-soft set, algorithms and applications for decision-making

## Abstract

In this research article, we motivate and introduce the concept of possibility belief interval-valued N-soft sets. It has a great significance for enhancing the performance of decision-making procedures in many theories of uncertainty. The N-soft set theory is arising as an effective mathematical tool for dealing with precision and uncertainties more than the soft set theory. In this regard, we extend the concept of belief interval-valued soft set to possibility belief interval-valued N-soft set (by accumulating possibility and belief interval with N-soft set), and we also explain its practical calculations. To this objective, we defined related theoretical notions, for example, belief interval-valued N-soft set, possibility belief interval-valued N-soft set, their algebraic operations, and examined some of their fundamental properties. Furthermore, we developed two algorithms by using max-AND and min-OR operations of possibility belief interval-valued N-soft set for decision-making problems and also justify its applicability with numerical examples.

## 1. Introduction

In real life, the limitation of precise research is progressively being recognized in various fields such as economics, social sciences, medical sciences, computer sciences, physical sciences, environmental sciences, management sciences, and engineering. It is familiar that the real world is full of vagueness, imprecision, and uncertainty, so research on these areas is of great significance. The solutions to such problems engaged the use of mathematical principles on the basis of imprecision and uncertainty. This article expands the scope of applications of one of the theories that can be used to deal with these attributes or characteristics, namely soft set theory.

In this unrealistic environment, there are many problems related to uncertainty [1,2,3,4]. However, the maximum mathematical tools that are in existence are crisp [5]. Numerous theories have been introduced to explore uncertainty in an efficient way. For instance, Bayesian network [6], evidence theory [7,8,9], fuzzy set theory [10,11], intuitionistic fuzzy set (IFS) [12,13], and gray prediction model [14]. Meanwhile, numerous properties of these theories have also been studied broadly [14]. In [15], Molodtsov indicates that there is a difficulty in the fuzzy set and intuitionistic fuzzy set theory, that is, the level of the membership defined by the individual regarded depends on the knowledge received by the individual, in consequence, vulnerable to subjective factors. Additionally, different attributes in one problem need to be thought about in an integrated manner. A soft set computing model was developed by Molodtsov [15] to address these restrictions. A problem considering multiple attributes is a virtue of the soft set theory and it has very good potential to solve problems and plays a very significant role in various fields [16,17]. Therefore, for soft set theory, many researchers are introducing methods and operations for it. For instance, the fuzzy soft set theory is an extension of the soft set proposed by Xu [18].

By accumulating soft set theory with the fuzzy set theory, Maji [19] introduced the notation of FSS (fuzzy soft set), and this theory was used to address decision-making problems. Generalized fuzzy soft sets were proposed by Majumdar and Samanta [20]; their properties were studied and used to solve problems of uncertainty. Maji [21,22] also introduced intuitionistic fuzzy soft sets by integrating IFS with the soft set. Dinda [23] introduced the generalized intuitionistic fuzzy soft sets, belief interval-valued soft sets [24], generalized belief interval-valued soft sets [25], interval-valued intuitionistic fuzzy soft sets [26], interval-valued picture fuzzy soft sets [27], interval-valued neutrosophic soft sets [28], and generalized picture fuzzy soft sets [29]. Further, there are many extension models of the soft set theory rapidly developed; for instance, possibility fuzzy soft set [30], possibility m-polar fuzzy soft sets [31], possibility Pythagorean fuzzy soft sets [32], possibility neutrosophic soft sets [33], possibility multi-fuzzy soft sets [34], and possibility belief interval-valued soft sets [35].

The belief theory was proposed by Dempster and Shafer [35,36]. This theory has been applied in various fields. For instance, uncertainty modeling [37], uncertainty reasoning [14,38,39], decision-making [40,41], information fusion [42,43], and other fields [44]. Fatimah [45] extended the soft set model under a non-binary evaluation environment and introduced the concept of N-soft set (NSS) and explained the significance of ordered grades in the practical problems. Furthermore, they also developed decision-making procedures for the N-soft set. Later on, Akram [46] proposed a novel hybrid model known as hesitant N-soft set (HNSS) by accumulating hesitancy and N-soft set. Meanwhile, in [47], they also introduced the concept of fuzzy N-soft set (FNSS) by accumulating a fuzzy set with an N-soft set. Many problems related to decision-making are discussed by using different kinds of environments in [48,49,50,51,52,53,54,55,56,57,58]. The developed model gives a more flexible decision-making method for dealing with uncertainties referring to which specific level is allocated to objects in the parameterizations by attributes.

In this article, we present the concept of a possibility belief interval-valued N-soft set, which can be viewable as a possibility belief interval-valued N-soft model. In Section 2, we review the basic idea concerning the Dempster–Shafer theory and in addition, soft set, belief interval-valued soft set (BIVSS) and N-soft set are briefly reminded of with examples. In Section 3, we propose the model of the belief interval-valued N-soft set (BIVNSS). In Section 4, we discuss some algebraic operations (for instance, restricted intersection, restricted union, extended intersection, extended union, complement, top complement, bottom complement, max-AND, and min-OR) on the belief interval-valued N-soft set and many fundamental properties of these operations are introduced. In Section 5, we proposed the model of possibility belief interval-valued N-soft set (PBIVNSS). In Section 6, we introduce many algebraic operations (for example, restricted intersection, restricted union, extended intersection, extended union, complement, top complement, bottom complement, max-AND, and min-OR) on possibility belief interval-valued N-soft set, and various fundamental properties on these operations are also discussed. In Section 7, we develop algorithms on max-AND and min-OR operations of possibility belief interval-valued N-soft sets for decision making. Then in Section 8, we present the applications on decision-making problems that yield the optimum solution. While in Section 9, we conclude the article.

## 2. Preliminaries

In this section, a short review of basic definitions and relevant theories are given, which we used to develop the methods introduced in this paper. There are several problems related to uncertainty in this real-life [59,60,61]. The Dempster–Shafer theory has been broadly used in dealing with the uncertain problems [62,63]. The Dempster–Shafer theory is a generalized scheme for demonstrating uncertainty. Dempster proposed a belief measure theory that developed lower and upper probabilities of a system while Shafer provided a thorough belief function explanation.

**Definition** **1.** *Let Y be a finite set of frame of discernment (hypotheses), $2^{Y}$ be the set of all subsets of Y and $\hat{Y}\subseteq Y$. The belief structure of Dempster-Shafer is associated with a mapping $\varepsilon :2^{Y}\to \left[0,1\right]$ such that*

$$\begin{array}{c} \varepsilon \left(\Phi \right)=0,\sum_{\hat{Y}\in 2^{Y}}\varepsilon \left(\hat{Y}\right)=1, \end{array}$$

*is the basic probability assignment function, where $\varepsilon (\hat{Y})$ indicates the belief values of Y. For which subsets of Y mapping allot non-zero values are known as focal elements [24].*

*Basic probability assignment has various operations for instance divergence [64], entropy function [65,66,67], and others [68].*

**Definition** **2** ([35]). *The measure of belief function associated with ε is determined as a mapping $Bel:2^{Y}\to \left[0,1\right]$ such that for any subset $\hat{Z}$ of Y,*

$$\begin{array}{ccc} Bel(\hat{Z}) & = & \sum_{\Phi \neq \hat{Y}\subseteq \hat{Z}}\varepsilon \left(\hat{Y}\right). \end{array}$$

**Definition** **3** ([35]). *The measure of plausibility function associated with ε is determined as a mapping $Pl:2^{Y}\to \left[0,1\right]$ such that for any subset $\hat{Z}$ of Y,*

$$\begin{array}{c} Pl\left(\hat{Z}\right)=\sum_{\hat{Y}\cap \hat{Z}\neq \Phi }\varepsilon \left(\hat{Y}\right). \end{array}$$

Obviously, $Bel\left(\hat{Z}\right)\leq Pl\left(\hat{Z}\right)$. The interval $\left[Bel\left(\hat{Z}\right),Pl\left(\hat{Z}\right)\right]$ is called belief interval (BI) [69].

**Definition** **4.** *Let U be the non-empty universal set of objects and E be the set of attributes, for any non-empty set K⊆E. A pair (A,K) is called soft set over U if there is a mapping $A:K\to 2^{U}$ where $2^{U}$ denotes the set of all subsets of U.*

*Thus, the soft set is a parametric family of the subsets of a universal set. For each $k_{j}\in K$, we can interpret $A(k_{j})$ as a subset of universal set U. We can also consider $A(k_{j})$ as a mapping $A\left(k_{j}\right):U\to \left\{0,1\right\}$ and then $A\left(k_{j}\right)\left(u_{i}\right)=1$ equivalent to $u_{i}\in A\left(k_{j}\right)$, otherwise $A\left(k_{j}\right)\left(u_{i}\right)=0$ [45]. Molodtsov considered many examples in [15] to illustrate the soft set.*

**Definition** **5.** *Let U be the non-empty universal set of objects. Let $BI^{U}$ denote the collection of all belief interval-valued subsets of U and E be the set of attributes, for any non-empty set K⊆E. A pair (B,K) is called a belief interval-valued soft set over U (in short BIVSS) if there is a mapping*$B:K\to BI^{U}$. *It is represented as:*

$$\begin{array}{c} B\left(k_{j}\right)=\{\frac{u_{i}}{BI_{B(k_{j})}\left(u_{i}\right)}\;|\;u_{i}\in U\wedge k_{j}\in K\}, \end{array}$$

*where $BI_{B(k_{j})}\left(u_{i}\right)=\left[Bel_{B(k_{j})}\left(u_{i}\right),Pl_{B(k_{j})}\left(u_{i}\right)\right],Bel_{B(k_{j})}\left(u_{i}\right)\in \left[0,1\right],Pl_{B(k_{j})}\left(u_{i}\right)\in \left[0,1\right]$, and $0\leq Bel_{B(k_{j})}\left(u_{i}\right)$$\leq Pl_{B(k_{j})}\left(u_{i}\right)\leq 1$  for all $u_{i}\in U$.*

**Example** **1.** *Let $U=\{u_{1},u_{2},u_{3},u_{4},u_{5}\}$ be the set of universe, $E=\{k_{1},k_{2},k_{3},k_{4}\}$ be the set of attributes, and K⊆E such that $K=\{k_{1},k_{2}\}$. Then BIVSS over U is:*

$$B\left(k_{1}\right)=\{\frac{u_{1}}{\left[0.5,1.0\right]},\frac{u_{2}}{\left[0.4,0.7\right]},\frac{u_{3}}{\left[0.2,0.4\right]}\},\;\;B\left(k_{2}\right)=\{\frac{u_{1}}{\left[0.2,0.7\right]},\frac{u_{2}}{\left[0.1,0.6\right]},\frac{u_{3}}{\left[0.3,0.9\right]}\}.$$

**Definition** **6.** *Let U be the non-empty universal set of objects. Let $2^{U}$ denote the set of all subsets of U and let R={0,1,2,…,N−1} be a set of ordered grades where N∈{2,3,4,…} and E are the set of attributes, for any non-empty set K⊆E. A triple (C,K,N) is called N-soft set over U if there is a mapping $C:K\to 2^{U\times R}$, with the property that for each $k_{j}\in K$ there exists a unique $\left(u_{i},r_{ij}\right)\in \left(U\times R\right)$ such that $\left(u_{i},r_{ij}\right)\in C\left(k_{j}\right),\;k_{j}\in K,\;u_{i}\in U\;and\;r_{ij}\in R$, where $2^{U\times R}$ is the collection of all soft sets over U×R [45].*

**Example** **2.** *Let $U=\{u_{1},u_{2},u_{3}\}$ be the set of students, $E=\{k_{1},k_{2},k_{3},k_{4},k_{5}\}$ be the set of attributes evaluations of students by skills, and K⊆E such that*

*$K=\{k_{1}=communication\;skills,k_{3}=collaboration\;skills,k_{5}=critical\;thinking\}$ and let R={0,1,2,3,4,5} be the set of grade evaluation. Then, (C,K,6) is the 6-soft set as follows:*

$C\left(k_{1}\right)=\{\left(u_{1},4\right),\left(u_{2},2\right),\left(u_{3},3\right)\},\;\;C\left(k_{3}\right)=\{\left(u_{1},2\right),\left(u_{2},1\right),\left(u_{3},5\right)\},\;and\;C\left(k_{5}\right)=\{\left(u_{1},5\right),\left(u_{2},3\right),\left(u_{3},0\right)\}.$

*It can also be represented in tabular form as follows:*


(C,K,6)



$k_{1}$



$k_{3}$



$k_{5}$



$u_{1}$

425

$u_{2}$

213

$u_{3}$

350

For illustration, the above table is of a 6-soft set (C,K,6) established on communication skills, collaboration skills, and critical thinking of the students. Where in the top left cell 4 is the ordered grade $\left(r_{11}\right)$ of the student $u_{1}$ with respect to $k_{1}$ = communication skills. Similarly, in the bottom right cell, 0 is the ordered grade $\left(r_{35}\right)$ of the student $u_{3}$ with respect to $k_{5}$ = critical thinking. Here, 0 is the lowest grade; it does not mean that there is no evaluation or incomplete information. There are many examples to illustrate the N-soft set in [45].

## 3. Belief Interval-Valued N-Soft Set (BIVNSS)

In this section, we derive some basic concepts of a new extended model of a belief interval-valued N-soft set with examples from real practice.

**Definition** **7.** *Let U be the non-empty universal set of objects and E be the set of attributes, for any non-empty set K⊆E and let $BI^{U}$ denote the collection of all belief interval-valued subsets of U and R={0,1,2,…,N−1} be a set of ordered grades where N∈{2,3,4,…}. A triple (A,K,N) is called a belief interval valued N-soft set over U if there is a mapping $A:K\to BI^{U\times R}$, where $BI^{U\times R}$ is the collection of all belief interval-valued soft sets over U×R. It is represented as:*

$$\begin{array}{c} A\left(k_{j}\right)=\{\frac{\left(u_{i},r_{ij}\right)}{BI_{A(k_{j})}\left(u_{i},r_{ij}\right)}\;|\;\left(u_{i},r_{ij}\right)\in \left(U\times R\right)\},\;\;\forall k_{j}\in K\subseteq E, \end{array}$$

*where,*

$$BI_{A(k_{j})}\left(u_{i},r_{ij}\right)=\left[Bel_{A(k_{j})}\left(u_{i},r_{ij}\right),Pl_{A(k_{j})}\left(u_{i},r_{ij}\right)\right],Bel_{A(k_{j})}\left(u_{i},r_{ij}\right)\in \left[0,1\right]$$

*and*

$$Pl_{A(k_{j})}\left(u_{i},r_{ij}\right)\in \left[0,1\right],0\leq Bel_{A(k_{j})}\left(u_{i},r_{ij}\right)\leq Pl_{A(k_{j})}\left(u_{i},r_{ij}\right)\leq 1\;;\;\forall \;u_{i}\in U\;and\;r_{ij}\in R.$$

**Example** **3.** *Let $U=\{u_{1},u_{2},u_{3}\}$ be the universe of gardens, R={0,1,2,3,4} be the set of grade evaluation, $E=\{k_{1}=Rose,\;k_{2}=Tulip,\;k_{3}=Jasmine,\;k_{4}=Daffodils\}$ be the set of attributes (evaluation of gardens by flowers), and K⊆E such that $K=\{k_{1},k_{3},k_{4}\}$.*

*Thus,* (A,K,5) *is the belief interval of 5−soft set as follow:*

$$\left(A,K,5\right)=\{(k_{1},(\frac{\left(u_{1},3\right)}{\left[0.4,0.8\right]},\frac{\left(u_{2},0\right)}{\left[0.1,0.7\right]},\frac{\left(u_{3},4\right)}{\left[0.5,0.8\right]})),(k_{3},(\frac{\left(u_{1},2\right)}{\left[0.3,0.5\right]},\frac{\left(u_{2},4\right)}{\left[0.8,0.9\right]},\frac{\left(u_{3},1\right)}{\left[0.2,0.4\right]})),(k_{4},(\frac{\left(u_{1},1\right)}{\left[0.7,0.9\right]},\frac{\left(u_{2},3\right)}{\left[0.3,0.8\right]},\frac{\left(u_{3},2\right)}{\left[0.4,0.7\right]}))\}.$$

## 4. Operations on BIVNSS

In this section, we discussed some algebraic operations on belief interval-valued N-soft set and their properties.

**Definition** **8.** *Let U be the non-empty universal set of objects. Given that (B,K,M) and (C,L,N) are two BIVNSS on U, their restricted intersection is defined as:*

$$\left(D,T,O\right)=\left(B,K,M\right)\cap_{R}\left(C,L,N\right),$$

*where $D=B\cap_{R}C,\;\;T=K\cap L\neq \Phi$ and $O=min\left(M,N\right),i.e.,\;\forall t_{j}\in T\wedge u_{i}=U,$*

$$\begin{array}{cc} \frac{\left(u_{i},r_{ij}\right)}{BI_{D(t_{j})}\left(u_{i},r_{ij}\right)}\in D\left(t_{j}\right)\;\Leftrightarrow \; & r_{ij}=min\left(r_{ij}^{1},r_{ij}^{2}\right),\\ \; & BI_{D(t_{j})}\left(u_{i},r_{ij}\right)=[Bel_{D(t_{j})}\left(u_{i},r_{ij}\right),Pl_{D(t_{j})}\left(u_{i},r_{ij}\right)] \end{array}$$

*where,*

$$\begin{array}{c} \begin{array}{cc} {}[Bel_{D(t_{j})}\left(u_{i},r_{ij}\right),Pl_{D(t_{j})}\left(u_{i},r_{ij}\right)]=[ & min(Bel_{D(t_{j})}\left(u_{i},r_{ij}^{1}\right),Bel_{D(t_{j})}\left(u_{i},r_{ij}^{2}\right)),\\ & min(Pl_{D(t_{j})}\left(u_{i},r_{ij}^{1}\right),Pl_{D(t_{j})}\left(u_{i},r_{ij}^{2}\right))]. \end{array} \end{array}$$

*If $\frac{\left(u_{i},r_{ij}^{1}\right)}{\left[Bel_{D(t_{j})}\left(u_{i},r_{ij}^{1}\right),Pl_{D(t_{j})}\left(u_{i},r_{ij}^{1}\right)\right]}\in B\left(t_{j}^{1}\right)\;and\;\frac{\left(u_{i},r_{ij}^{2}\right)}{\left[BelD(t_{j})\left(u_{i},r_{ij}^{2}\right),Pl_{D(t_{j})}\left(u_{i},r_{ij}^{2}\right)\right]}\in C\left(t_{j}^{2}\right)$ with $t_{j}^{1}\in K\;and\;t_{j}^{2}\in L.$*

**Definition** **9.** *Let U be the non-empty universal set of objects. Given that (B,K,M) and (C,L,N) are two BIVNSS on U, their restricted union is defined as:*

$$\left(E,T,P\right)=\left(B,K,M\right)\cup_{R}\left(C,L,N\right),$$

*where $E=B\cup_{R}C,\;\;T=K\cap L$ and $P=max\left(M,N\right),i.e.,\;\forall t_{j}\in T\;and\;u_{i}=U$,*

$$\begin{array}{cc} \frac{\left(u_{i},r_{ij}\right)}{BI_{E(t_{j})}\left(u_{i},r_{ij}\right)}\in E\left(t_{j}\right)\;\Leftrightarrow \; & r_{ij}=max\left(r_{ij}^{1},r_{ij}^{2}\right),\\ \; & BI_{E(t_{j})}\left(u_{i},r_{ij}\right)=[Bel_{E(t_{j})}\left(u_{i},r_{ij}\right),Pl_{E(t_{j})}\left(u_{i},r_{ij}\right)], \end{array}$$

*where,*

$$\begin{array}{c} \begin{array}{cc} {}[Bel_{E(t_{j})}\left(u_{i},r_{ij}\right),Pl_{E(t_{j})}\left(u_{i},r_{ij}\right)]=[ & max(Bel_{E(t_{j})}\left(u_{i},r_{ij}^{1}\right),Bel_{E(t_{j})}\left(u_{i},r_{ij}^{2}\right)),\\ & max(Pl_{E(t_{j})}\left(u_{i},r_{ij}^{1}\right),Pl_{E(t_{j})}\left(u_{i},r_{ij}^{2}\right))]. \end{array} \end{array}$$

*If $\frac{\left(u_{i},r_{ij}^{1}\right)}{\left[Bel_{E(t_{j})}\left(u_{i},r_{ij}^{1}\right),Pl_{E(t_{j})}\left(u_{i},r_{ij}^{1}\right)\right]}\in B\left(t_{j}^{1}\right)\;and\;\frac{\left(u_{i},r_{ij}^{2}\right)}{\left[BelE(t_{j})\left(u_{i},r_{ij}^{2}\right),Pl_{E(t_{j})}\left(u_{i},r_{ij}^{2}\right)\right]}\in C\left(t_{j}^{2}\right)$ with $t_{j}^{1}\in K\;and\;t_{j}^{2}\in L.$*

**Definition** **10.** *Let U be the non-empty universal set of objects. Given that (B,K,M) and (C,L,N) are two BIVNSS on U, their extended intersection is defined as:*

$$\left(F,S,P\right)=\left(B,K,M\right)\cap_{E}\left(C,L,N\right),$$

*where* $F=B\cap_{E}C,\;\;S=K\cup L$ *and* $P=max\left(M,N\right),i.e.,\;\forall s_{j}\in S,\;u_{i}=U\;with\;s_{j}^{1}\in K,\;and\;s_{j}^{2}\in L,$

$$\;F\left(s_{j}\right)=\left\{\begin{array}{cc} \;B(s_{j}^{1}) & ,\;\mathrm{if}\;\;s_{j}\in K-L,\\ C(s_{j}^{2}) & ,\;\mathrm{if}\;\;s_{j}\in L-K,\\ B\left(s_{j}^{1}\right)\cap_{R}C\left(s_{j}^{2}\right) & ,\;\mathrm{if}\;\;s_{j}\in K\cap L. \end{array}\right.$$

**Definition** **11.** *Let U be the non-empty universal set of objects. Given that (B,K,M) and (C,L,N) are two BIVNSS on U, their extended union is defined as:*

$$\left(H,S,P\right)=\left(B,K,M\right)\cup_{E}\left(C,L,N\right),$$

*where*$H=B\cup_{E}C,\;\;S=K\cup L$ and $P=max\left(M,N\right),i.e.,\;\forall s_{j}\in S,u_{i}=U\;with\;\;s_{j}^{1}\in K\;and\;s_{j}^{2}\in L$,

$$\;H\left(s_{j}\right)=\left\{\begin{array}{cc} \;B(s_{j}^{1}) & ,\;\mathrm{if}\;\;s_{j}\in K-L,\\ \;C(s_{j}^{2}) & ,\;\mathrm{if}\;\;s_{j}\in L-K,\\ \;B\left(s_{j}^{1}\right)\cup_{R}C\left(s_{j}^{2}\right) & ,\;\mathrm{if}\;\;s_{j}\in L\cap K. \end{array}\right.$$

**Example** **4.** *Let $U=\{u_{1},u_{2},u_{3}\}$ be the set of Covid-19 patients, $E=\{k_{1}=tiredness,\;k_{2}=skin\;rashes,\;k_{3}=dry\;cough,\;k_{4}=shortness\;of\;breath\}$ be the set of attributes and K,L⊆E such that $K=\left\{k_{1},k_{3},k_{4}\right\},\;\;L=\left\{k_{1},k_{3}\right\}$. The BIVNSS are defined as follows:*

$$\begin{array}{c} \begin{array}{cc} (B,K,5)=\{ & (k_{1},(\frac{\left(u_{1},1\right)}{\left[0.3,0.8\right]},\frac{\left(u_{2},4\right)}{\left[0.1,0.5\right]},\frac{\left(u_{3},2\right)}{\left[0.8,0.9\right]})),(k_{3},(\frac{\left(u_{1},3\right)}{\left[0.1,0.5\right]},\frac{\left(u_{2},1\right)}{\left[0.3,0.4\right]},\frac{\left(u_{3},4\right)}{\left[0.5,0.9\right]})),\\ & (k_{4},(\frac{\left(u_{1},2\right)}{\left[0.3,0.7\right]},\frac{\left(u_{2},4\right)}{\left[0.4,0.6\right]},\frac{\left(u_{3},3\right)}{\left[0.3,0.8\right]}))\}. \end{array} \end{array}$$

$$\begin{array}{c} \begin{array}{c} \left(C,L,4\right)=\{(k_{1},(\frac{\left(u_{1},0\right)}{\left[0.3,0.7\right]},\frac{\left(u_{2},1\right)}{\left[0.1,0.4\right]},\frac{\left(u_{3},3\right)}{\left[0.7,0.8\right]})),(k_{3},(\frac{\left(u_{1},2\right)}{\left[0.5,0.8\right]},\frac{\left(u_{2},2\right)}{\left[0.4,0.9\right]},\frac{\left(u_{3},0\right)}{\left[0.7,0.8\right]}))\}. \end{array} \end{array}$$

*Then their restricted intersection is:*

$$\begin{array}{c} \begin{array}{cc} (D,T,4)=\{ & (k_{1},(\frac{\left(u_{1},0\right)}{\left[0.3,0.7\right]},\frac{\left(u_{2},1\right)}{\left[0.1,0.4\right]},\frac{\left(u_{3},2\right)}{\left[0.7,0.8\right]})),(k_{3},(\frac{\left(u_{1},2\right)}{\left[0.1,0.5\right]},\frac{\left(u_{2},1\right)}{\left[0.3,0.4\right]},\frac{\left(u_{3},0\right)}{\left[0.5,0.8\right]}))\}. \end{array} \end{array}$$

*their restricted union is:*

$$\begin{array}{c} \begin{array}{cc} (E,T,5)=\{ & (k_{1},(\frac{\left(u_{1},1\right)}{\left[0.3,0.8\right]},\frac{\left(u_{2},4\right)}{\left[0.1,0.5\right]},\frac{\left(u_{3},3\right)}{\left[0.8,0.9\right]})),(k_{3},(\frac{\left(u_{1},3\right)}{\left[0.5,0.8\right]},\frac{\left(u_{2},2\right)}{\left[0.4,0.9\right]},\frac{\left(u_{3},4\right)}{\left[0.7,0.9\right]}))\}. \end{array} \end{array}$$

*their extended intersection is:*

$$\begin{array}{c} \begin{array}{cc} (F,S,5)=\{ & \left(k_{1},\left(\frac{\left(u_{1},0\right)}{\left[0.3,0.7\right]},\frac{\left(u_{2},1\right)}{\left[0.1,0.4\right]},\frac{\left(u_{3},2\right)}{\left[0.7,0.8\right]}\right)\right),\left(k_{3},\left(\frac{\left(u_{1},2\right)}{\left[0.1,0.5\right]},\frac{\left(u_{2},1\right)}{\left[0.3,0.4\right]},\frac{\left(u_{3},0\right)}{\left[0.5,0.8\right]}\right)\right),\\ & \left(k_{4},\left(\frac{\left(u_{1},2\right)}{\left[0.3,0.7\right]},\frac{\left(u_{2},4\right)}{\left[0.4,0.6\right]},\frac{\left(u_{3},3\right)}{\left[0.3,0.8\right]}\right)\right)\}. \end{array} \end{array}$$

*their extended union is:*

$$\begin{array}{c} \begin{array}{cc} (H,S,5)=\{ & \left(k_{1},\left(\frac{\left(u_{1},1\right)}{\left[0.3,0.8\right]},\frac{\left(u_{2},4\right)}{\left[0.1,0.5\right]},\frac{\left(u_{3},3\right)}{\left[0.8,0.9\right]}\right)\right),\left(k_{3},\left(\frac{\left(u_{1},3\right)}{\left[0.5,0.8\right]},\frac{\left(u_{2},2\right)}{\left[0.4,0.9\right]},\frac{\left(u_{3},4\right)}{\left[0.7,0.9\right]}\right)\right),\\ & \left(k_{4},\left(\frac{\left(u_{1},2\right)}{\left[0.3,0.7\right]},\frac{\left(u_{2},4\right)}{\left[0.4,0.6\right]},\frac{\left(u_{3},3\right)}{\left[0.3,0.8\right]}\right)\right)\}. \end{array} \end{array}$$

**Definition** **12.** *Let (A,K,N) be a BIVNSS on a non-empty universe U. Then a weak belief interval-valued complement is denoted by $\left(A^{c},K,N\right)$ where $A^{c}\left(k_{j}\right)\cap A\left(k_{j}\right)=\Phi ;\;\forall k_{j}\in K$ and $A^{c}\left(k_{j}\right)$ is defined as:*

$$\begin{array}{c} A^{c}\left(k_{j}\right)=\{\frac{\left(u_{i},r_{ij}\right)}{BI_{A^{c}\left(k_{j}\right)}\left(u_{i},r_{ij}\right)}\;|\;\left(u_{i},r_{ij}\right)\in U\times R\}. \end{array}$$

*where, $BI_{A^{c}\left(k_{j}\right)}\left(u_{i},r_{ij}\right)=\left[1-Pl_{A(k_{j})}\left(u_{i},r_{ij}\right),1-Bel_{A(k_{j})}\left(u_{i},r_{ij}\right)\right]$.*

**Definition** **13.** *For any BIVNSS(A,K,N) on U. The bottom weak belief interval-valued complement $\left(A^{<},K,N\right)$ of (A,K,N) is defined as: $\left(A^{<},K,N\right)=A\left(k_{j}\right)=$*

$$\left\{\begin{array}{cc} \frac{\left(u_{i},0\right)}{\left[1-Pl_{A(k_{j})}\left(u_{i},r_{ij}\right),1-Bel_{A(k_{j})}\left(u_{i},r_{ij}\right)\right]} & ,\;\mathrm{if}\;\;r_{ij}\;>\;0,\\ \frac{\left(u_{i},N-1\right)}{\left[1-Pl_{A(k_{j})}\left(u_{i},r_{ij}\right),1-Bel_{A(k_{j})}\left(u_{i},r_{ij}\right)\right]} & ,\;\mathrm{if}\;\;r_{ij}\;=\;0. \end{array}\right.$$

**Definition** **14.** *For any BIVNSS(A,K,N) on U. The top weak belief interval-valued complement $\left(A^{>},K,N\right)$ of (A,K,N) is defined as: $\left(A^{>},K,N\right)=A\left(k_{j}\right)=$*

$$\left\{\begin{array}{cc} \frac{\left(u_{i},N-1\right)}{\left[1-Pl_{A(k_{j})}\left(u_{i},r_{ij}\right),1-Bel_{A(k_{j})}\left(u_{i},r_{ij}\right)\right]} & ,\;\mathrm{if}\;\;r_{ij}\;<\;N-1,\\ \frac{\left(u_{i},0\right)}{\left[1-Pl_{A(k_{j})}\left(u_{i},r_{ij}\right),1-Bel_{A(k_{j})}\left(u_{i},r_{ij}\right)\right]} & ,\;\mathrm{if}\;\;r_{ij}\;=\;N-1. \end{array}\right.$$

**Example** **5.** *Consider (A,K,5) as described in Example 3 then its weak belief interval-valued complement is:*

$$\begin{array}{c} \begin{array}{cc} (A^{c},K,5)=\{ & \left(k_{1},\left(\frac{\left(u_{1},3\right)}{\left[0.2,0.6\right]},\frac{\left(u_{2},0\right)}{\left[0.3,0.9\right]},\frac{u_{3},4}{\left[0.2,0.5\right]}\right)\right),\left(k_{3},\left(\frac{\left(u_{1},2\right)}{\left[0.5,0.7\right]},\frac{\left(u_{2},4\right)}{\left[0.1,0.2\right]},\frac{u_{3},1}{\left[0.6,0.8\right]}\right)\right),\\ & \left(k_{4},\left(\frac{\left(u_{1},1\right)}{\left[0.1,0.3\right]},\frac{\left(u_{2},3\right)}{\left[0.2,0.7\right]},\frac{u_{3},2}{\left[0.3,0.6\right]}\right)\right)\}. \end{array} \end{array}$$

*its bottom weak belief interval valued complement is:*

$$\begin{array}{c} \begin{array}{cc} (A^{<},K,5)=\{ & \left(k_{1},\left(\frac{\left(u_{1},0\right)}{\left[0.2,0.6\right]},\frac{\left(u_{2},4\right)}{\left[0.3,0.9\right]},\frac{\left(u_{3},0\right)}{\left[0.2,0.5\right]}\right)\right),\left(k_{3},\left(\frac{\left(u_{1},0\right)}{\left[0.5,0.7\right]},\frac{\left(u_{2},0\right)}{\left[0.1,0.2\right]},\frac{\left(u_{3},0\right)}{\left[0.6,0.8\right]}\right)\right),\\ & \left(k_{4},\left(\frac{\left(u_{1},0\right)}{\left[0.1,0.3\right]},\frac{\left(u_{2},0\right)}{\left[0.2,0.7\right]},\frac{\left(u_{3},0\right)}{\left[0.3,0.6\right]}\right)\right)\}. \end{array} \end{array}$$

*its top weak belief interval-valued complement is:*

$$\begin{array}{c} \begin{array}{cc} (A^{>},K,5)=\{ & \left(k_{1},\left(\frac{\left(u_{1},4\right)}{\left[0.2,0.6\right]},\frac{\left(u_{2},4\right)}{\left[0.3,0.9\right]},\frac{\left(u_{3},0\right)}{\left[0.2,0.5\right]}\right)\right),\left(k_{3},\left(\frac{\left(u_{1},4\right)}{\left[0.5,0.7\right]},\frac{\left(u_{2},0\right)}{\left[0.1,0.2\right]},\frac{\left(u_{3},4\right)}{\left[0.6,0.8\right]}\right)\right),\\ & \left(k_{4},\left(\frac{\left(u_{1},4\right)}{\left[0.1,0.3\right]},\frac{\left(u_{2},4\right)}{\left[0.2,0.7\right]},\frac{\left(u_{3},4\right)}{\left[0.3,0.6\right]}\right)\right)\}. \end{array} \end{array}$$

**Definition** **15.** *Soft max-AND operation of two BIVNSS(B,K,M) and (C,L,N) (where $B:K\to BI^{U\times R}$ and $C:L\to BI^{U\times R})$ defined as:*

(B,K,M)∧(C,L,N)=(G,K×L,P),

$$where,G:K\times L\to BI^{U\times R}\;;\;\forall \left(k_{s^{\prime }},l_{t^{\prime }}\right)\in \left(K\times L\right),\;s^{\prime },t^{\prime }\in \Lambda ,\;and\;P=max\left(M,N\right),$$

$$\begin{array}{c} \begin{array}{cc} G(k_{s^{\prime }},l_{t^{\prime }})\in & \frac{\left(u_{i},r_{i(s^{\prime },t^{\prime })}\right)}{BI_{G(k_{s^{\prime }},l_{t^{\prime }})}\left(u_{i},r_{i(s^{\prime },t^{\prime })}\right)}\Leftrightarrow r_{i(s^{\prime },t^{\prime })}=max\left(r_{i(s^{\prime },t^{\prime })}^{1},r_{i(s^{\prime },t^{\prime })}^{2}\right),\\ & BI_{G(k_{s^{\prime }},l_{t^{\prime }})}\left(u_{i},r_{i(s^{\prime },t^{\prime })}\right)=\left[Bel_{G(k_{s^{\prime }},l_{t^{\prime }})}\left(u_{i},r_{i(s^{\prime },t^{\prime })}\right),Pl_{G(k_{s^{\prime }},l_{t^{\prime }})}\left(u_{i},r_{i(s^{\prime },t^{\prime })}\right)\right]. \end{array} \end{array}$$

$$\begin{array}{c} \begin{array}{cc} where,[Bel_{G(k_{s^{\prime }},l_{t^{\prime }})}\left(u_{i},r_{i(s^{\prime },t^{\prime })}\right),Pl_{G(k_{s^{\prime }},l_{t^{\prime }})} & (u_{i},r_{i(s^{\prime },t^{\prime })})]=\\ & [\frac{2}{3}\left(Bel_{B(k_{s^{\prime }})}\left(u_{i},r_{i(s^{\prime },t^{\prime })}\right)+Bel_{C(l_{t^{\prime }})}\left(u_{i},r_{i(s^{\prime },t^{\prime })}\right)\right)-\\ & \frac{1}{3}max\left\{Bel_{B(k_{s^{\prime }})}\left(u_{i},r_{i(s^{\prime },t^{\prime })}\right),Bel_{C(l_{t^{\prime }})}\left(u_{i},r_{i(s^{\prime },t^{\prime })}\right)\right\},\\ & \frac{2}{3}\left(Pl_{B(k_{s^{\prime }})}\left(u_{i},r_{i(s^{\prime },t^{\prime })}\right)+Pl_{C(l_{t^{\prime }})}\left(u_{i},r_{i(s^{\prime },t^{\prime })}\right)\right)-\\ & \frac{1}{3}max\left\{Pl_{B(k_{s^{\prime }})}\left(u_{i},r_{i(s^{\prime },t^{\prime })}\right),Pl_{C(l_{t^{\prime }})}\left(u_{i},r_{i(s^{\prime },t^{\prime })}\right)\right\}] \end{array} \end{array}$$

*with $\left(u_{i},r_{i(s^{\prime },t^{\prime })}^{1}\right)\in B\left(K\right)$ and $\left(u_{i},r_{i(s^{\prime },t^{\prime })}^{2}\right)\in C\left(L\right)$.*

**Definition** **16.** *Soft min-OR operation of two BIVNSS(B,K,M) and (C,L,N) (where $B:K\to BI^{U\times R}$ and $C:L\to BI^{U\times R})$ defined as:*

(B,K,M)∨(C,L,N)=(Q,K×L,O),

*where, $Q:K\times L\to BI^{U\times R}\;;\;\forall \left(k_{s^{\prime }},l_{t^{\prime }}\right)\in \left(K\times L\right),\;s^{\prime },t^{\prime }\in \Lambda ,\;and\;O=min\left(M,N\right)$*

$$\begin{array}{c} \begin{array}{cc} Q(k_{s^{\prime }},l_{t^{\prime }})\in & \frac{\left(u_{i},r_{i(s^{\prime },t^{\prime })}\right)}{BI_{Q(k_{s^{\prime }},l_{t^{\prime }})}\left(u_{i},r_{i(s^{\prime },t^{\prime })}\right)}\Leftrightarrow r_{i(s^{\prime },t^{\prime })}=min\left(r_{i(s^{\prime },t^{\prime })}^{1},r_{i(s^{\prime },t^{\prime })}^{2}\right),\\ & BI_{Q(k_{s^{\prime }},l_{t^{\prime }})}\left(u_{i},r_{i(s^{\prime },t^{\prime })}\right)=\left[Bel_{Q(k_{s^{\prime }},l_{t^{\prime }})}\left(u_{i},r_{i(s^{\prime },t^{\prime })}\right),Pl_{Q(k_{s^{\prime }},l_{t^{\prime }})}\left(u_{i},r_{i(s^{\prime },t^{\prime })}\right)\right]. \end{array} \end{array}$$

*where,*

$$\begin{array}{c} \begin{array}{cc} {}[Bel_{Q(k_{s^{\prime }},l_{t^{\prime }})}\left(u_{i},r_{i(s^{\prime },t^{\prime })}\right),Pl_{Q(k_{s^{\prime }},l_{t^{\prime }})} & (u_{i},r_{i(s^{\prime },t^{\prime })})]=\\ & [\frac{2}{3}\left(Bel_{B(k_{s^{\prime }})}\left(u_{i},r_{i(s^{\prime },t^{\prime })}\right)+Bel_{C(l_{t^{\prime }})}\left(u_{i},r_{i(s^{\prime },t^{\prime })}\right)\right)-\\ & \frac{1}{3}min\left\{Bel_{B(k_{s^{\prime }})}\left(u_{i},r_{i(s^{\prime },t^{\prime })}\right),Bel_{C(l_{t^{\prime }})}\left(u_{i},r_{i(s^{\prime },t^{\prime })}\right)\right\},\\ & \frac{2}{3}\left(Pl_{B(k_{s^{\prime }})}\left(u_{i},r_{i(s^{\prime },t^{\prime })}\right)+Pl_{C(l_{t^{\prime }})}\left(u_{i},r_{i(s^{\prime },t^{\prime })}\right)\right)-\\ & \frac{1}{3}min\left\{Pl_{B(k_{s^{\prime }})}\left(u_{i},r_{i(s^{\prime },t^{\prime })}\right),Pl_{C(l_{t^{\prime }})}\left(u_{i},r_{i(s^{\prime },t^{\prime })}\right)\right\}] \end{array} \end{array}$$

*with $\left(u_{i},r_{i(s^{\prime },t^{\prime })}^{1}\right)\in B\left(K\right)$ and $\left(u_{i},r_{i(s^{\prime },t^{\prime })}^{2}\right)\in C\left(L\right)$.*

**Example** **6.** *Consider (B,K,5) and (C,L,4) as described in Example 4, then their soft max-AND is:*

$$\begin{array}{c} \begin{array}{cc} (G,K\times L,5)=\{ & \left(\left(k_{1},k_{1}\right),\left(\frac{\left(u_{1},1\right)}{\left[0.30,0.73\right]},\frac{\left(u_{2},40\right)}{\left[0.10,0.43\right]},\frac{\left(u_{3},3\right)}{\left[0.73,0.83\right]}\right)\right),\\ & \;\;\left(\left(k_{1},k_{3}\right),\left(\frac{\left(u_{1},2\right)}{\left[0.37,0.80\right]},\frac{\left(u_{2},4\right)}{\left[0.20,0.63\right]},\frac{\left(u_{3},2\right)}{\left[0.73,0.83\right]}\right)\right),\\ & \;\;\left(\left(k_{4},k_{1}\right),\left(\frac{\left(u_{1},2\right)}{\left[0.30,0.70\right]},\frac{\left(u_{2},4\right)}{\left[0.20,0.47\right]},\frac{\left(u_{3},3\right)}{\left[0.43,0.80\right]}\right)\right),\\ & \;\;\left(\left(k_{4},k_{3}\right),\left(\frac{\left(u_{1},2\right)}{\left[0.37,0.73\right]},\frac{\left(u_{2},4\right)}{\left[0.40,0.70\right]},\frac{\left(u_{3},3\right)}{\left[0.43,0.80\right]}\right)\right),\\ & \;\;\left(\left(k_{3},k_{1}\right),\left(\frac{\left(u_{1},3\right)}{\left[0.17,0.57\right]},\frac{\left(u_{2},1\right)}{\left[0.17,0.40\right]},\frac{\left(u_{3},4\right)}{\left[0.57,0.83\right]}\right)\right),\\ & \;\;\left(\left(k_{3},k_{3}\right),\left(\frac{\left(u_{1},3\right)}{\left[0.23,0.60\right]},\frac{\left(u_{2},2\right)}{\left[0.30,0.57\right]},\frac{\left(u_{3},4\right)}{\left[0.57,0.83\right]}\right)\right)\}. \end{array} \end{array}$$

*their soft min-OR is:*

$$\begin{array}{c} \begin{array}{cc} (Q,K\times L,4)=\{ & \left(\left(k_{1},k_{1}\right),\left(\frac{\left(u_{1},0\right)}{\left[0.30,0.76\right]},\frac{\left(u_{2},1\right)}{\left[0.10,0.46\right]},\frac{\left(u_{3},2\right)}{\left[0.76,0.86\right]}\right)\right),\\ & \;\;\left(\left(k_{1},k_{3}\right),\left(\frac{\left(u_{1},1\right)}{\left[0.43,0.80\right]},\frac{\left(u_{2},2\right)}{\left[0.30,0.76\right]},\frac{\left(u_{3},0\right)}{\left[0.76,0.86\right]}\right)\right),\\ & \;\;\left(\left(k_{4},k_{1}\right),\left(\frac{\left(u_{1},0\right)}{\left[0.30,0.70\right]},\frac{\left(u_{2},1\right)}{\left[0.30,0.53\right]},\frac{\left(u_{3},3\right)}{\left[0.56,0.80\right]}\right)\right),\\ & \;\;\left(\left(k_{4},k_{3}\right),\left(\frac{\left(u_{1},2\right)}{\left[0.43,0.76\right]},\frac{\left(u_{2},2\right)}{\left[0.40,0.80\right]},\frac{\left(u_{3},0\right)}{\left[0.56,0.80\right]}\right)\right),\\ & \;\;\left(\left(k_{3},k_{1}\right),\left(\frac{\left(u_{1},0\right)}{\left[0.23,0.63\right]},\frac{\left(u_{2},1\right)}{\left[0.23,0.40\right]},\frac{\left(u_{3},3\right)}{\left[0.63,0.86\right]}\right)\right),\\ & \;\;\left(\left(k_{3},k_{3}\right),\left(\frac{\left(u_{1},2\right)}{\left[0.36,0.70\right]},\frac{\left(u_{2},1\right)}{\left[0.36,0.73\right]},\frac{\left(u_{3},0\right)}{\left[0.63,0.86\right]}\right)\right)\}. \end{array} \end{array}$$

**Proposition** **1.** *Given that (B,K,M),(C,L,N), and (D,T,O) are any three BIVNSS on U, then the commutative and associative properties are held:*

*(1)* 
$\left(B,K,M\right)\cap_{E}\left(B,K,M\right)=\left(B,K,M\right),$

*(2)* 
$\left(B,K,M\right)\cap_{R}\left(B,K,M\right)=\left(B,K,M\right),$

*(3)* 
$\left(B,K,M\right)\cup_{E}\left(B,K,M\right)=\left(B,K,M\right),$

*(4)* 
$\left(B,K,M\right)\cup_{R}\left(B,K,M\right)=\left(B,K,M\right),$

*(5)*  *$\left(B,K,M\right)\cap_{E}\left(C,L,N\right)=\left(C,L,N\right)\cap_{E}\left(B,K,M\right)$,*
*(6)*  *$\left(B,K,M\right)\cap_{R}\left(C,L,N\right)=\left(C,L,N\right)\cap_{R}\left(B,K,M\right)$,*
*(7)*  *$\left(B,K,M\right)\cup_{E}\left(C,L,N\right)=\left(C,L,N\right)\cup_{E}\left(B,K,M\right)$,*
*(8)*  *$\left(B,K,M\right)\cup_{R}\left(C,L,N\right)=\left(C,L,N\right)\cup_{R}\left(B,K,M\right)$,*
*(9)*  *$\left(B,K,M\right)\cap_{E}\left(\left(C,L,N\right)\cap_{E}\left(D,T,O\right)\right)=\left(\left(B,K,M\right)\cap_{E}\left(C,L,N\right)\right)\cap_{E}\left(D,T,O\right)$,*
*(10)*  *$\left(B,K,M\right)\cap_{R}\left(\left(C,L,N\right)\cap_{R}\left(D,T,O\right)\right)=\left(\left(B,K,M\right)\cap_{R}\left(C,L,N\right)\right)\cap_{R}\left(D,T,O\right)$,*
*(11)*  *$\left(B,K,M\right)\cup_{E}\left(\left(C,L,N\right)\cup_{E}\left(D,T,O\right)\right)=\left(\left(B,K,M\right)\cup_{E}\left(C,L,N\right)\right)\cup_{E}\left(D,T,O\right)$,*
*(12)*  *$\left(B,K,M\right)\cup_{R}\left(\left(C,L,N\right)\cup_{R}\left(D,T,O\right)\right)=\left(\left(B,K,M\right)\cup_{R}\left(C,L,N\right)\right)\cup_{R}\left(D,T,O\right)$.*

**Proof.**  (1)–(8) follows from the definition. (9) Let (C,L,N) and (D,T,O) (where $C:L\to BI^{U\times R}$ and $D:T\to BI^{U\times R}$) be two BIVNSS. By the definition of extended intersection we have $\left(S,V,Z\right)\left(where\;S:V\to BI^{U\times R}\right)$ such that,

$$\left(S,V,Z\right)=\left(C,L,N\right)\cap_{E}\left(D,T,O\right),$$

where $S=C\cap_{E}D,\;V=L\cup T,\;Z=max\left(N,O\right)\;;\forall v_{j}\in V$ with $v_{j}^{1}\in L$ and $v_{j}^{2}\in T$,

$$\;S\left(v_{j}\right)=\left\{\begin{array}{cc} \;C(v_{j}^{1}) & ,\;\mathrm{if}\;\;v_{j}\in L-T,\\ \;D(v_{j}^{2}) & ,\;\mathrm{if}\;\;v_{j}\in T-L,\\ \;C\left(v_{j}^{1}\right)\cap_{R}D\left(v_{j}^{2}\right) & ,\;\;\mathrm{if}\;\;v_{j}\in L\cap T. \end{array}\right.$$

As, $\left(B,K,M\right)\cap_{E}\left(\left(C,L,N\right)\cap_{E}\left(D,T,O\right)\right)=\left(B,K,M\right)\cap_{E}\left(S,V,Z\right).$ Suppose that $\left(B,K,M\right)\cap_{E}$(S,V,Z)=(G,W,X) such that $G:W\to BI^{U\times R}$, where $G=B\cap_{E}S,\;W=K\cup V=K\cup L\cup T,\;X=max\left(M,Z\right)\;;\;\forall w_{j}\in W$ with $w_{j}^{1}\in K$, $w_{j}^{2}\in L$ and $w_{j}^{3}\in T$,

$$G\left(w_{j}\right)=\left\{\begin{array}{cc} \;B(w_{j}^{1}) & ,\;\mathrm{if}\;\;w_{j}\in K-L-T,\\ \;C(w_{j}^{2}) & ,\;\mathrm{if}\;\;w_{j}\in L-K-T,\\ \;D(w_{j}^{3}) & ,\;\mathrm{if}\;\;w_{j}\in T-K-L,\\ \;C\left(w_{j}^{2}\right)\cap_{R}D\left(w_{j}^{3}\right) & ,\;\mathrm{if}\;\;w_{j}\in L\cap T-K,\\ \;B\left(w_{j}^{1}\right)\cap_{R}C\left(w_{j}^{2}\right) & ,\;\mathrm{if}\;\;w_{j}\in K\cap L-T,\\ \;B\left(w_{j}^{1}\right)\cap_{R}D\left(w_{j}^{3}\right) & ,\;\mathrm{if}\;\;w_{j}\in K\cap T-L,\\ \;B\left(w_{j}^{1}\right)\cap_{R}C\left(w_{j}^{2}\right)\cap_{R}D\left(w_{j}^{3}\right) & ,\;\;\mathrm{if}\;\;w_{j}\in K\cap L\cap T. \end{array}\right.$$

Again, let (B,K,M) and (C,L,N) (where $B:K\to BI^{U\times R}$ and $C:L\to BI^{U\times R}$) be two BIVNSS. By the definition of extended intersection we have (F,S,P) where $\left(F:S\to BI^{U\times R}\right)$ such that,

$$\left(F,S,P\right)=\left(B,K,M\right)\cap_{E}\left(C,L,N\right),$$

where $F=B\cap_{E}C,\;S=K\cup L,\;P=max\left(M,N\right)\;;\;\forall s_{j}\in S$ with $s_{j}^{1}\in K$ and $s_{j}^{2}\in L,$

$$\;F\left(s_{j}\right)=\left\{\begin{array}{cc} \;B(s_{j}^{1}) & ,\;\mathrm{if}\;\;s_{j}\in K-L,\\ \;C(s_{j}^{2}) & ,\;\mathrm{if}\;\;s_{j}\in L-K,\\ \;B\left(s_{j}^{1}\right)\cap_{R}C\left(s_{j}^{2}\right) & ,\;\;\mathrm{if}\;\;s_{j}\in K\cap L. \end{array}\right.$$

As, $\left(\left(B,K,M\right)\cap_{E}\left(C,L,N\right)\right)\cap_{E}\left(D,T,O\right)=\left(F,S,P\right)\cap_{E}\left(D,T,O\right)$. Suppose that $\left(F,S,P\right)\cap_{E}\left(D,T,O\right)=\left(I,W,X\right)$ such that $I:W\to BI^{U\times R},$ where $I=F\cap_{E}D,\;W=S\cup T=K\cup L\cup T,\;X=max\left(P,O\right)\;;\;\forall w_{j}\in W$ with $w_{j}^{1}\in K$, $w_{j}^{2}\in L$ and $w_{j}^{3}\in T$,

$$I\left(w_{j}\right)=\left\{\begin{array}{cc} \;B(w_{j}^{1}) & ,\;\mathrm{if}\;\;w_{j}\in K-L-T,\\ \;C(w_{j}^{2}) & ,\;\mathrm{if}\;\;w_{j}\in L-K-T,\\ \;D(w_{j}^{3}) & ,\;\mathrm{if}\;\;w_{j}\in T-K-L,\\ \;C\left(w_{j}^{2}\right)\cap_{R}D\left(w_{j}^{3}\right) & ,\;\mathrm{if}\;\;w_{j}\in L\cap T-K,\\ \;B\left(w_{j}^{1}\right)\cap_{R}C\left(w_{j}^{2}\right) & ,\;\mathrm{if}\;\;w_{j}\in K\cap L-T,\\ \;B\left(w_{j}^{1}\right)\cap_{R}D\left(w_{j}^{3}\right) & ,\;\mathrm{if}\;\;w_{j}\in K\cap T-L,\\ \;B\left(w_{j}^{1}\right)\cap_{R}C\left(w_{j}^{2}\right)\cap_{R}D\left(w_{j}^{3}\right) & ,\;\;\mathrm{if}\;\;w_{j}\in K\cap L\cap T. \end{array}\right.$$

Then $G\left(w_{j}\right)=I\left(w_{j}\right),\;\forall w_{j}\in W$. Hence, (9) is held, (10)-(12) are similar to (9).    □

**Proposition** **2.** *Given that (B,K,M) and (C,L,N) are any two BIVNSS on U, then the following results hold:*

*(1)*  *$\left({\left(B^{c}\right)}^{c},K,M\right)=\left(B,K,M\right)$,*
*(2)*  *$\left(B^{c},K,M\right)\cup_{E}\left(C^{c},L,N\right)=\left({\left(B\cap_{E}C\right)}^{c},\left(K\cup L\right),max\left(M,N\right)\right)$,*
*(3)*  *$\left(B^{c},K,M\right)\cap_{E}\left(C^{c},L,N\right)=\left({\left(B\cup_{E}C\right)}^{c},\left(K\cup L\right),max\left(M,N\right)\right)$,*
*(4)*  *$\left(B^{c},K,M\right)\cup_{R}\left(C^{c},L,N\right)=\left({\left(B\cap_{R}C\right)}^{c},\left(K\cap L\right),max\left(M,N\right)\right)$,*
*(5)*  *$\left(B^{c},K,M\right)\cap_{R}\left(C^{c},L,N\right)=\left({\left(B\cup_{R}C\right)}^{c},\left(K\cap L\right),min\left(M,N\right)\right)$.*

**Proof.**  (1) is straight-forward. We start from (2), let (B,K,M) and (C,L,N) (where $B:K\to BI^{U\times R}$$C:L\to BI^{U\times R}$) be two BIVNSS on *U*. Then by the definition of weak belief interval-valued complement and extended union we have,

$$\left(B^{c},K,M\right)\cup_{E}\left(C^{c},L,N\right)=\left(J,S,P\right),$$

where $J=B^{c}\cup_{E}C^{c},\;S=K\cup L,\;P=max\left(M,N\right);\forall s_{j}\in S$ with $s_{j}^{1}\in K$ and $s_{j}^{2}\in L$,

$$\;J\left(s_{j}\right)=\left\{\begin{array}{cc} \;B^{c}\left(s_{j}^{1}\right) & ,\;\mathrm{if}\;\;s_{j}\in K-L,\\ \;C^{c}\left(s_{j}^{2}\right) & ,\;\mathrm{if}\;\;s_{j}\in L-K,\\ \;B^{c}\left(s_{j}^{1}\right)\cup_{E}C^{c}\left(s_{j}^{2}\right) & ,\;\;\mathrm{if}\;\;s_{j}\in K\cap L. \end{array}\right.$$

Again by the definition of extended intersection we have $\left(F,S,P\right)=\left(B\cap_{E}C\right),\;S=K\cup L,\;P=max\left(M,N\right);\forall s_{j}\in S$ with $s_{j}^{1}\in K$ and $s_{j}^{2}\in L$,

$$\;F\left(s_{j}\right)=\left\{\begin{array}{cc} \;B(s_{j}^{1}) & ,\;\mathrm{if}\;\;s_{j}\in K-L,\\ \;C(s_{j}^{2}) & ,\;\mathrm{if}\;\;s_{j}\in L-K,\\ \;B\left(s_{j}^{1}\right)\cap_{E}C\left(s_{j}^{2}\right) & ,\;\;\mathrm{if}\;\;s_{j}\in K\cap L. \end{array}\right.$$

Thus, by the definition of weak complement we have,

$$\;F^{c}\left(s_{j}\right)=\left\{\begin{array}{cc} \;B^{c}\left(s_{j}^{1}\right) & ,\;\mathrm{if}\;\;s_{j}\in K-L,\\ \;C^{c}\left(s_{j}^{2}\right) & ,\;\mathrm{if}\;\;s_{j}\in L-K,\\ \;B^{c}\left(s_{j}^{1}\right)\cup_{E}C^{c}\left(s_{j}^{2}\right) & ,\;\;\mathrm{if}\;\;s_{j}\in K\cap L. \end{array}\right.$$

Then, $J\left(s_{j}\right)=F^{c}\left(s_{j}\right);\;\forall s_{j}\in S$. Hence, (2) holds, and (3)–(5) are similar to (2).    □

## 5. Possibility Belief Interval-Valued N-Soft Set (PBIVNSS)

In this section, we defined the notion of possibility belief interval-valued *N*-soft set.

**Definition** **17.** *Assume that $BI^{U}$ is the set of all belief interval-valued subsets of U and E is the set of attributes, for any non-empty set K⊆E. The pair (U,K) is called a soft universe, and R={0,1,2,…,N−1} is a set of ordered grades where N∈{2,3,4,…}. Let $B:K\to BI^{U\times R}$, and b is a belief interval-valued subsets of K, i.e., $b:K\to BI^{U\times R}$ where $BI^{U\times R}$ is the collection of all belief interval-valued soft sets over U×R. A triple $\left(B_{b},K,N\right)$ is called a possibility belief interval-valued N-soft set over (U,K) if there is a mapping $B_{b}:K\to BI^{U\times R}\times BI^{U\times R}$. It is represented as:*

$$\begin{array}{c} \begin{array}{cc} B_{b}\left(k_{j}\right)\left(u_{i},r_{ij}\right)=\{ & \left[Bel_{B(k_{j})}\left(u_{i},r_{ij}\right),Pl_{B(k_{j})}\left(u_{i},r_{ij}\right)\right],\left[Bel_{b(k_{j})}\left(u_{i},r_{ij}\right),Pl_{b(k_{j})}\left(u_{i},r_{ij}\right)\right]\} \end{array} \end{array}$$

*such that $(u_{i},r_{ij})\in U\times R$ for all $k_{j}\in K\subseteq E.$*

**Example** **7.** *Let $U=\left\{u_{1},u_{2},u_{3}\right\},\;\;R=\left\{0,1,2,3,4,5,6\right\},E=\left\{k_{1},k_{2},k_{3},k_{4}\right\}$ and K⊆E such that $K=\{k_{1},k_{2},k_{3}\}$. Then,$\left(B_{b},K,7\right)$ is the possibility belief interval of 7−soft set as follows:*

$$\begin{array}{c} \begin{array}{cc} (B_{b},K,7)=\{ & \left(k_{1},\left(\frac{\left(u_{1},1\right)}{\left[0.3,0.8],[0.3,0.7\right]},\frac{\left(u_{2},5\right)}{\left[0.3,0.4],[0.1,0.5\right]},\frac{\left(u_{3},2\right)}{\left[0.4,0.6],[0.3,0.5\right]}\right)\right),\\ & \left(k_{2},\left(\frac{\left(u_{1},4\right)}{\left[0.6,0.7],[0.5,0.7\right]},\frac{\left(u_{2},0\right)}{\left[0.1,0.8],[0.7,0.9\right]},\frac{\left(u_{3},6\right)}{\left[0.6,0.8],[0.4,0.7\right]}\right)\right),\\ & \left(k_{3},\left(\frac{\left(u_{1},6\right)}{\left[0.1,0.2],[0.4,0.5\right]},\frac{\left(u_{2},3\right)}{\left[0.8,0.9],[0.7,0.8\right]},\frac{\left(u_{3},5\right)}{\left[0.8,0.9],[0.3,0.6\right]}\right)\right)\}. \end{array} \end{array}$$

## 6. Operations on PBIVNSS

In this section, we discussed some algebraic operations on a possibility belief interval valued N-soft set and their fundamental properties.

**Definition** **18.** *Let U be the non-empty universal set of objects. Given that $\left(B_{b},K,M\right)$ and $\left(Y_{y},L,N\right)$ are two PBIVNSS on U, their restricted intersection is defined as:*

$$\left(D_{d},T,O\right)=\left(B_{b},K,M\right){⊓}_{R}\left(Y_{y},L,N\right),$$

*where $D=B\cap_{R}Y,\;\;d=b\cap_{R}y,\;\;T=K\cap L\neq \Phi$ and $O=min\left(M,N\right),\;i.e.,\;\forall t_{j}\in T\;and\;u_{i}=U$,*

$$\begin{array}{c} \begin{array}{cc} \frac{\left(u_{i},r_{ij}\right)}{BI_{D(t_{j})}\left(u_{i},r_{ij}\right),BI_{d(t_{j})}\left(u_{i},r_{ij}\right)}\in D_{d}\left(t_{j}\right) & \;\Leftrightarrow r_{ij}=min\left(r_{ij}^{1},r_{ij}^{2}\right),\\ & BI_{D(t_{j})}\left(u_{i},r_{ij}\right)=\left[Bel_{D(t_{j})}\left(u_{i},r_{ij}\right),Pl_{D(t_{j})}\left(u_{i},r_{ij}\right)\right],\\ & BI_{d(t_{j})}\left(u_{i},r_{ij}\right)=\left[Bel_{d(t_{j})}\left(u_{i},r_{ij}\right),Pl_{d(t_{j})}\left(u_{i},r_{ij}\right)\right]. \end{array} \end{array}$$

*where,*

$$\begin{array}{c} \begin{array}{cc} \left[Bel_{D(t_{j})}\left(u_{i},r_{ij}\right),Pl_{D(t_{j})}\left(u_{i},r_{ij}\right)\right]= & [min\left(Bel_{D(t_{j})}\left(u_{i},r_{ij}^{1}\right),Bel_{D(t_{j})}\left(u_{i},r_{ij}^{2}\right)\right),\\ & \;\;min\left(Pl_{D(t_{j})}\left(u_{i},r_{ij}^{1}\right),Pl_{D(t_{j})}\left(u_{i},r_{ij}^{2}\right)\right)] \end{array} \end{array}$$

*and*

$$\begin{array}{c} \begin{array}{cc} \left[Bel_{d(t_{j})}\left(u_{i},r_{ij}\right),Pl_{d(t_{j})}\left(u_{i},r_{ij}\right)\right]= & [min\left(Bel_{d(t_{j})}\left(u_{i},r_{ij}^{1}\right),Bel_{d(t_{j})}\left(u_{i},r_{ij}^{2}\right)\right),\\ & \;\;min\left(Pl_{d(t_{j})}\left(u_{i},r_{ij}^{1}\right),Pl_{d(t_{j})}\left(u_{i},r_{ij}^{2}\right)\right)]. \end{array} \end{array}$$

*If $\left(\left(u_{i},r_{ij}^{1}\right),\;\left[Bel_{D(t_{j})}\left(u_{i},r_{ij}^{1}\right),Pl_{D(t_{j})}\left(u_{i},r_{ij}^{1}\right)\right],\left[Bel_{d(t_{j})}\left(u_{i},r_{ij}^{1}\right),Pl_{d(t_{j})}\left(u_{i},r_{ij}^{1}\right)\right]\right)\in B_{b}\left(t_{j}^{1}\right)$ and $\left(\left(u_{i},r_{ij}^{2}\right),\;\left[Bel_{D(t_{j})}\left(u_{i},r_{ij}^{2}\right),Pl_{D(t_{j})}\left(u_{i},r_{ij}^{2}\right)\right],\left[Bel_{d(t_{j})}\left(u_{i},r_{ij}^{2}\right),Pl_{d(t_{j})}\left(u_{i},r_{ij}^{2}\right)\right]\right)\in Y_{y}\left(t_{j}^{2}\right),$ with $t_{j}^{1}\in K\;and\;t_{j}^{2}\in L.$*

**Definition** **19.** *Let U be the non-empty universal set of objects. Given that $\left(B_{b},K,M\right)$ and $\left(Y_{y},L,N\right)$ are two PBIVNSS on U, their restricted union is defined as:*

$$\left(E_{e},T,P\right)=\left(B_{b},K,M\right){⊔}_{R}\left(Y_{y},L,N\right)$$

*where $E=B\cup_{R}Y,\;e=b\;\cup_{R}\;y,\;T=K\;\cap \;L\;and\;P=max\left(M,N\right),i.e.,\;\forall t_{j}\in T\;and\;u_{i}=U$,*

$$\begin{array}{c} \begin{array}{cc} \frac{\left(u_{i},r_{ij}\right)}{BI_{E(t_{j})}\left(u_{i},r_{ij}\right),BI_{e(t_{j})}\left(u_{i},r_{ij}\right)}\in E_{e}\left(t_{j}\right)\; & \Leftrightarrow r_{ij}=max\left(r_{ij}^{1},r_{ij}^{2}\right),\\ & BI_{E(t_{j})}\left(u_{i},r_{ij}\right)=\left[Bel_{E(t_{j})}\left(u_{i},r_{ij}\right),Pl_{E(t_{j})}\left(u_{i},r_{ij}\right)\right],\\ & BI_{e(t_{j})}\left(u_{i},r_{ij}\right)=\left[Bel_{e(t_{j})}\left(u_{i},r_{ij}\right),Pl_{e(t_{j})}\left(u_{i},r_{ij}\right)\right]. \end{array} \end{array}$$

*where,*

$$\begin{array}{c} \begin{array}{cc} \left[Bel_{E(t_{j})}\left(u_{i},r_{ij}\right),Pl_{E(t_{j})}\left(u_{i},r_{ij}\right)\right]= & [max\left(Bel_{E(t_{j})}\left(u_{i},r_{ij}^{1}\right),Bel_{E(t_{j})}\left(u_{i},r_{ij}^{2}\right)\right),\\ & \;max\left(Pl_{E(t_{j})}\left(u_{i},r_{ij}^{1}\right),Pl_{E(t_{j})}\left(u_{i},r_{ij}^{2}\right)\right)] \end{array} \end{array}$$

*and*

$$\begin{array}{c} \begin{array}{cc} \left[Bel_{e(t_{j})}\left(u_{i},r_{ij}\right),Pl_{e(t_{j})}\left(u_{i},r_{ij}\right)\right]= & [max\left(Bel_{e(t_{j})}\left(u_{i},r_{ij}^{1}\right),Bel_{e(t_{j})}\left(u_{i},r_{ij}^{2}\right)\right),\\ & \;max\left(Pl_{e(t_{j})}\left(u_{i},r_{ij}^{1}\right),Pl_{e(t_{j})}\left(u_{i},r_{ij}^{2}\right)\right)]. \end{array} \end{array}$$

*If $\left(\left(u_{i},r_{ij}^{1}\right),\;\left[Bel_{E(t_{j})}\left(u_{i},r_{ij}^{1}\right),Pl_{E(t_{j})}\left(u_{i},r_{ij}^{1}\right)\right],\left[Bel_{e(t_{j})}\left(u_{i},r_{ij}^{1}\right),Pl_{e(t_{j})}\left(u_{i},r_{ij}^{1}\right)\right]\right)\in B_{b}\left(t_{j}^{1}\right)$ and $\left(\left(u_{i},r_{ij}^{2}\right),\;\left[Bel_{E(t_{j})}\left(u_{i},r_{ij}^{2}\right),Pl_{E(t_{j})}\left(u_{i},r_{ij}^{2}\right)\right],\left[Bel_{e(t_{j})}\left(u_{i},r_{ij}^{2}\right),Pl_{e(t_{j})}\left(u_{i},r_{ij}^{2}\right)\right]\right)\;\in Y_{y}\left(t_{j}^{2}\right),$ with $t_{j}^{1}\in K\;and\;t_{j}^{2}\in L.$*

**Definition** **20.** *Let U be the non-empty universal set of objects. Given that $\left(B_{b},K,M\right)$ and $\left(Y_{y},L,N\right)$ are two PBIVNSS on U, their extended intersection is defined as:*

$$\left(F_{f},S,P\right)=\left(B_{b},K,M\right){⊓}_{E}\left(Y_{y},L,N\right)$$

*where $F=B\cap_{E}Y,\;\;f=b\cap_{E}y,\;\;S=K\cup L$ and $P=max\left(M,N\right),i.e.,\;\forall s_{j}\in S,\;u_{i}=U\;with\;\;s_{j}^{1}\in K\;and\;s_{j}^{2}\in L,$*

$$\;F_{f}\left(s_{j}\right)=\left\{\begin{array}{cc} \;B_{b}\left(s_{j}^{1}\right) & ,\;\mathrm{if}\;\;s_{j}\in K-L,\\ \;Y_{y}\left(s_{j}^{2}\right) & ,\;\mathrm{if}\;\;s_{j}\in L-K,\\ \;B_{b}\left(s_{j}^{1}\right){⊓}_{R}Y_{y}\left(s_{j}^{2}\right) & ,\;\mathrm{if}\;\;s_{j}\in L\cap K. \end{array}\right.$$

**Definition** **21.** *Let U be the non-empty universal set of objects. Given that $\left(B_{b},K,M\right)$ and $\left(Y_{y},L,N\right)$ are two PBIVNSS on U, their extended union is defined as:*

$$\left(H_{h},S,P\right)=\left(B_{b},K,M\right){⊔}_{E}\left(Y_{y},L,N\right)$$

*where $H=B\cup_{E}Y,\;\;h=b\cup_{E}y,\;\;S=K\cup L$ and $P=max\left(M,N\right),i.e.,\;\forall s_{j}\in S,u_{i}=U,\;u_{i}=U$$with\;s_{j}^{1}\in K\;and\;s_{j}^{2}\in L,$*

$$\;H_{h}\left(s_{j}\right)=\left\{\begin{array}{cc} \;B_{b}\left(s_{j}^{1}\right) & ,\;\mathrm{if}\;\;s_{j}\in K-L,\\ \;Y_{y}\left(s_{j}^{2}\right) & ,\;\mathrm{if}\;\;s_{j}\in L-K,\\ \;B_{b}\left(s_{j}^{1}\right){⊔}_{R}Y_{y}\left(s_{j}^{2}\right) & ,\;\mathrm{if}\;\;s_{j}\in L\cap K. \end{array}\right.$$

**Example** **8.** *Let $U=\left\{u_{1},u_{2},u_{3}\right\},\;\;E=\left\{k_{1},k_{2},k_{3},k_{4}\right\}$ and K∧L⊆E such that $K=\left\{k_{1},k_{2},k_{3}\right\},\;\;L=\left\{k_{2},k_{4}\right\}$. Then PBIVNSS are defined as follows:*

$$\begin{array}{c} \begin{array}{cc} (B_{b},K,7)=\{ & \left(k_{1},\left(\frac{\left(u_{1},1\right)}{\left[0.3,0.8],[0.3,0.7\right]},\frac{\left(u_{2},5\right)}{\left[0.3,0.4],[0.1,0.5\right]},\frac{\left(u_{3},2\right)}{\left[0.4,0.6],[0.3,0.5\right]}\right)\right),\\ & \left(k_{2},\left(\frac{\left(u_{1},4\right)}{\left[0.6,0.7],[0.5,0.7\right]},\frac{\left(u_{2},0\right)}{\left[0.1,0.8],[0.7,0.9\right]},\frac{\left(u_{3},6\right)}{\left[0.6,0.8],[0.4,0.7\right]}\right)\right),\\ & \left(k_{3},\left(\frac{\left(u_{1},6\right)}{\left[0.1,0.2],[0.4,0.5\right]},\frac{\left(u_{2},3\right)}{\left[0.8,0.9],[0.7,0.8\right]},\frac{\left(u_{3},5\right)}{\left[0.8,0.9],[0.3,0.6\right]}\right)\right)\}. \end{array} \end{array}$$

$$\begin{array}{c} \begin{array}{cc} (Y_{y},L,6)=\{ & \left(k_{2},\left(\frac{\left(u_{1},2\right)}{\left[0.2,0.6],[0.4,0.5\right]},\frac{\left(u_{2},1\right)}{\left[0.2,0.5],[0.1,0.7\right]},\frac{\left(u_{3},4\right)}{\left[0.2,0.7],[0.3,0.7\right]}\right)\right),\\ & \left(k_{4},\left(\frac{\left(u_{1},0\right)}{\left[0.6,0.9],[0.5,0.8\right]},\frac{\left(u_{2},5\right)}{\left[0.5,0.7],[0.4,0.8\right]},\frac{\left(u_{3},3\right)}{\left[0.3,0.9],[0.1,0.9\right]}\right)\right)\}. \end{array} \end{array}$$

*Then their restricted intersection is:*

$$\begin{array}{c} \left(D_{d},T,6\right)=\{\left(k_{2},\left(\frac{\left(u_{1},2\right)}{\left[0.2,0.6],[0.4,0.5\right]},\frac{\left(u_{2},0\right)}{\left[0.1,0.5],[0.1,0.7\right]},\frac{\left(u_{3},4\right)}{\left[0.2,0.7],[0.3,0.7\right]}\right)\right)\}. \end{array}$$

*their restricted union is:*

$$\begin{array}{c} \left(E_{e},T,7\right)=\{\left(k_{2},\left(\frac{\left(u_{1},4\right)}{\left[0.6,0.7],[0.5,0.7\right]},\frac{\left(u_{2},1\right)}{\left[0.2,0.8],[0.7,0.9\right]},\frac{\left(u_{3},6\right)}{\left[0.6,0.8],[0.4,0.7\right]}\right)\right)\}. \end{array}$$

*their extended intersection is*

$$\begin{array}{c} \begin{array}{cc} (F_{f},S,7)=\{ & \left(k_{1},\left(\frac{\left(u_{1},1\right)}{\left[0.3,0.8],[0.3,0.7\right]},\frac{\left(u_{2},5\right)}{\left[0.3,0.4],[0.1,0.5\right]},\frac{\left(u_{3},2\right)}{\left[0.4,0.6],[0.3,0.5\right]}\right)\right),\\ & \left(k_{2},\left(\frac{\left(u_{1},2\right)}{\left[0.2,0.6],[0.4,0.5\right]},\frac{\left(u_{2},0\right)}{\left[0.1,0.5],[0.1,0.7\right]},\frac{\left(u_{3},4\right)}{\left[0.2,0.7],[0.3,0.7\right]}\right)\right),\\ & \left(k_{3},\left(\frac{\left(u_{1},6\right)}{\left[0.1,0.2],[0.4,0.5\right]},\frac{\left(u_{2},3\right)}{\left[0.8,0.9],[0.7,0.8\right]},\frac{\left(u_{3},5\right)}{\left[0.8,0.9],[0.3,0.6\right]}\right)\right),\\ & \left(k_{4},\left(\frac{\left(u_{1},0\right)}{\left[0.6,0.9],[0.5,0.8\right]},\frac{\left(u_{2},5\right)}{\left[0.5,0.7],[0.4,0.8\right]},\frac{\left(u_{3},3\right)}{\left[0.3,0.9],[0.1,0.9\right]}\right)\right)\}. \end{array} \end{array}$$

*their extended union is:*

$$\begin{array}{c} \begin{array}{cc} (H_{h},S,7)=\{ & \left(k_{1},\left(\frac{\left(u_{1},1\right)}{\left[0.3,0.8],[0.3,0.7\right]},\frac{\left(u_{2},5\right)}{\left[0.3,0.4],[0.1,0.5\right]},\frac{\left(u_{3},2\right)}{\left[0.4,0.6],[0.3,0.5\right]}\right)\right),\\ & \left(k_{2},\left(\frac{\left(u_{1},4\right)}{\left[0.6,0.7],[0.5,0.7\right]},\frac{\left(u_{2},1\right)}{\left[0.2,0.8],[0.7,0.9\right]},\frac{\left(u_{3},6\right)}{\left[0.6,0.8],[0.4,0.7\right]}\right)\right),\\ & \left(k_{3},\left(\frac{\left(u_{1},6\right)}{\left[0.1,0.2],[0.4,0.5\right]},\frac{\left(u_{2},3\right)}{\left[0.8,0.9],[0.7,0.8\right]},\frac{\left(u_{3},5\right)}{\left[0.8,0.9],[0.3,0.6\right]}\right)\right),\\ & \left(k_{4},\left(\frac{\left(u_{1},0\right)}{\left[0.6,0.9],[0.5,0.8\right]},\frac{\left(u_{2},5\right)}{\left[0.5,0.7],[0.4,0.8\right]},\frac{\left(u_{3},3\right)}{\left[0.3,0.9],[0.1,0.9\right]}\right)\right)\}. \end{array} \end{array}$$

**Definition** **22.** *Let $\left(B_{b},K,N\right)$ be a PBIVNSS on a non-empty universe U. Then a weak possibility belief interval-valued complement is denoted by $\left(B_{b^{c}}^{c},K,N\right)$ where $B_{b^{c}}^{c}\left(k_{j}\right)\cap B_{b}\left(k_{j}\right)=\Phi ;\;\forall k_{j}\in K$ and $B_{b^{c}}^{c}\left(k_{j}\right)$ is defined as:*

$$B_{b^{c}}^{c}\left(k_{j}\right)=\left\{\frac{\left(u_{i},r_{ij}\right)}{BI_{B^{c}\left(k_{j}\right)}\left(u_{i},r_{ij}\right),BI_{b^{c}\left(k_{j}\right)}\left(u_{i},r_{ij}\right)}|\;\left(u_{i},r_{ij}\right)\in U\times R\right\},$$

$$where,\;\;\;\;\;\;\;\;\;BI_{B^{c}\left(k_{j}\right)}\left(u_{i},r_{ij}\right)=\left[1-Pl_{B(k_{j})}\left(u_{i},r_{ij}\right),1-Bel_{B(k_{j})}\left(u_{i},r_{ij}\right)\right],$$

$$\;\;\;\;\;\;\;\;\;\;\;\;\;\;\;\;\;\;BI_{b^{c}\left(k_{j}\right)}\left(u_{i},r_{ij}\right)=\left[1-Pl_{b(k_{j})}\left(u_{i},r_{ij}\right),1-Bel_{b(k_{j})}\left(u_{i},r_{ij}\right)\right].$$

**Definition** **23.** *For any $PBIVNSS\;(B_{b},K,N)$ on U. The bottom weak possibility belief interval-valued complement $\left(B_{b}^{≺},K,N\right)$ of $\left(B_{b},K,N\right)$ is defined as:*

*
$\left(B_{b}^{≺},K,N\right)=B_{b}\left(k_{j}\right)=$
*

$$\left\{\begin{array}{c} \frac{\left(u_{i},0\right)}{BI_{B^{c}\left(k_{j}\right)}\left(u_{i},r_{ij}\right),BI_{b^{c}\left(k_{j}\right)}\left(u_{i},r_{ij}\right)}\;,\mathrm{if}\;\;r_{ij}\;>\;0,\\ \;\frac{\left(u_{i},N-1\right)}{BI_{B^{c}\left(k_{j}\right)}\left(u_{i},r_{ij}\right),BI_{b^{c}\left(k_{j}\right)}\left(u_{i},r_{ij}\right)}\;,\mathrm{if}\;\;r_{ij}\;=\;0. \end{array}\right.$$

**Definition** **24.** *For any $PBIVNSS\;(B_{b},K,N)$ on U. The top weak possibility belief interval-valued complement $\left(B_{b}^{≻},K,N\right)$ of $\left(B_{b},K,N\right)$ is defined as:*

$$\left(B_{b}^{≻},K,N\right)=B_{b}\left(k_{j}\right)=\left\{\begin{array}{c} \frac{\left(u_{i},N-1\right)}{BI_{B^{c}\left(k_{j}\right)}\left(u_{i},r_{ij}\right),BI_{b^{c}\left(k_{j}\right)}\left(u_{i},r_{ij}\right)}\;,\mathrm{if}\;r_{ij}<\;N-1,\\ \;\frac{\left(u_{i},0\right)}{BI_{B^{c}\left(k_{j}\right)}\left(u_{i},r_{ij}\right),BI_{b^{c}\left(k_{j}\right)}\left(u_{i},r_{ij}\right)}\;,\mathrm{if}\;r_{ij}=\;N-1. \end{array}\right.$$

**Example** **9.** *Consider $\left(B_{b},K,7\right)$ as described in Example 7, then its weak possibility belief interval-valued complement is:*

$$\begin{array}{c} \begin{array}{cc} (B_{b^{c}}^{c},K,7)=\{ & \left(k_{1},\left(\frac{\left(u_{1},1\right)}{\left[0.2,0.7],[0.3,0.7\right]},\frac{\left(u_{2},5\right)}{\left[0.6,0.7],[0.5,0.9\right]},\frac{\left(u_{3},2\right)}{\left[0.4,0.6],[0.5,0.7\right]}\right)\right),\\ & \left(k_{2},\left(\frac{\left(u_{1},4\right)}{\left[0.3,0.4],[0.3,0.5\right]},\frac{\left(u_{2},0\right)}{\left[0.2,0.9],[0.1,0.3\right]},\frac{\left(u_{3},6\right)}{\left[0.2,0.4],[0.3,0.6\right]}\right)\right),\\ & \left(k_{3},\left(\frac{\left(u_{1},6\right)}{\left[0.8,0.9],[0.5,0.6\right]},\frac{\left(u_{2},3\right)}{\left[0.1,0.2],[0.2,0.3\right]},\frac{\left(u_{3},5\right)}{\left[0.1,0.2],[0.4,0.7\right]}\right)\right)\}. \end{array} \end{array}$$

*its bottom weak possibility belief interval-valued complement is:*

$$\begin{array}{c} \begin{array}{cc} (B_{b}^{≺},K,7)=\{ & \left(k_{1},\left(\frac{\left(u_{1},0\right)}{\left[0.2,0.7],[0.3,0.7\right]},\frac{\left(u_{2},0\right)}{\left[0.6,0.7],[0.5,0.9\right]},\frac{\left(u_{3},0\right)}{\left[0.4,0.6],[0.5,0.7\right]}\right)\right),\\ & \left(k_{2},\left(\frac{\left(u_{1},0\right)}{\left[0.3,0.4],[0.3,0.5\right]},\frac{\left(u_{2},6\right)}{\left[0.2,0.9],[0.1,0.3\right]},\frac{\left(u_{3},0\right)}{\left[0.2,0.4],[0.3,0.6\right]}\right)\right),\\ & \left(k_{3},\left(\frac{\left(u_{1},0\right)}{\left[0.8,0.9],[0.5,0.6\right]},\frac{\left(u_{2},0\right)}{\left[0.1,0.2],[0.2,0.3\right]},\frac{\left(u_{3},0\right)}{\left[0.1,0.2],[0.4,0.7\right]}\right)\right)\}. \end{array} \end{array}$$

*its top weak possibility belief interval-valued complement is:*

$$\begin{array}{c} \begin{array}{cc} (B_{b}^{≺},K,7)=\{ & \left(k_{1},\left(\frac{\left(u_{1},6\right)}{\left[0.2,0.7],[0.3,0.7\right]},\frac{\left(u_{2},6\right)}{\left[0.6,0.7],[0.5,0.9\right]},\frac{\left(u_{3},6\right)}{\left[0.4,0.6],[0.5,0.7\right]}\right)\right),\\ & \left(k_{2},\left(\frac{\left(u_{1},6\right)}{\left[0.3,0.4],[0.3,0.5\right]},\frac{\left(u_{2},6\right)}{\left[0.2,0.9],[0.1,0.3\right]},\frac{\left(u_{3},0\right)}{\left[0.2,0.4],[0.3,0.6\right]}\right)\right),\\ & \left(k_{3},\left(\frac{\left(u_{1},0\right)}{\left[0.8,0.9],[0.5,0.6\right]},\frac{\left(u_{2},6\right)}{\left[0.1,0.2],[0.2,0.3\right]},\frac{\left(u_{3},6s\right)}{\left[0.1,0.2],[0.4,0.7\right]}\right)\right)\}. \end{array} \end{array}$$

**Definition** **25.** *Soft max-AND operation of two PBIVNSS$\left(B_{b},K,M\right)$ and $\left(Y_{y},L,N\right)$ (where $B_{b}:K\to BI^{U\times R}\times BI^{U\times R}$ and $Y_{y}:L\to BI^{U\times R}\times BI^{U\times R})$ defined as:*

$$\left(B_{b},K,M\right)\hat{\wedge }\left(Y_{y},L,N\right)=\left(G_{g},K\times L,P\right),$$

*where $G_{g}:K\times L\to BI^{U\times R}\times BI^{U\times R}\;;\;\forall \left(k_{s^{\prime }},l_{t^{\prime }}\right)\in \left(K\times L\right),\;s^{\prime },t^{\prime }\in \Lambda ,\;and\;P=max\left(M,N\right),$*

$$\begin{array}{cc} G_{g}\left(k_{s^{\prime }},l_{t^{\prime }}\right)\in & \frac{\left(u_{i},r_{i(s^{\prime },t^{\prime })}\right)}{BI_{G(k_{s^{\prime }},l_{t^{\prime }})}\left(u_{i},r_{i(s^{\prime },t^{\prime })}\right),BI_{g(k_{s^{\prime }},l_{t^{\prime }})}\left(u_{i},r_{i(s^{\prime },t^{\prime })}\right)}\Leftrightarrow r_{i(s^{\prime },t^{\prime })}=max\left(r_{i(s^{\prime },t^{\prime })}^{1},r_{i(s^{\prime },t^{\prime })}^{2}\right),\\ \; & \;\;\;\;\;BI_{G(k_{s^{\prime }},l_{t^{\prime }})}\left(u_{i},r_{i(s^{\prime },t^{\prime })}\right)=\left[BelG(k_{s^{\prime }},l_{t^{\prime }})\left(u_{i},r_{i(s^{\prime },t^{\prime })}\right),Pl_{G(k_{s^{\prime }},l_{t^{\prime }})}\left(u_{i},r_{i(s^{\prime },t^{\prime })}\right)\right],\\ \; & \;\;\;\;\;BI_{g(k_{s^{\prime }},l_{t^{\prime }})}\left(u_{i},r_{i(s^{\prime },t^{\prime })}\right)=\;\left[Bel_{g(k_{s^{\prime }},l_{t^{\prime }})}\left(u_{i},r_{i(s^{\prime },t^{\prime })}\right),Pl_{g(k_{s^{\prime }},l_{t^{\prime }})}\left(u_{i},r_{i(s^{\prime },t^{\prime })}\right)\right]. \end{array}$$

*where,*

$$\begin{array}{cc} {}[BelG(k_{s^{\prime }},l_{t^{\prime }})\left(u_{i},r_{i(s^{\prime },t^{\prime })}\right), & Pl_{G(k_{s^{\prime }},l_{t^{\prime }})}\left(u_{i},r_{i(s^{\prime },t^{\prime })}\right)]=\\ \; & [\frac{2}{3}\left(Bel_{B(k_{s^{\prime }})}\left(u_{i},r_{i(s^{\prime },t^{\prime })}\right)+Bel_{Y(l_{t^{\prime }})}\left(u_{i},r_{i(s^{\prime },t^{\prime })}\right)\right)-\\ \; & \frac{1}{3}max\left\{Bel_{B(k_{s^{\prime }})}(u_{i},r_{i(s^{\prime },t^{\prime })},Bel_{Y(l_{t^{\prime }})}\left(u_{i},r_{i(s^{\prime },t^{\prime })}\right)\right\},\\ \; & \frac{2}{3}\left(Pl_{B(k_{s^{\prime }})}\left(u_{i},r_{i(s^{\prime },t^{\prime })}\right)+Pl_{Y(l_{t^{\prime }})}\left(u_{i},r_{i(s^{\prime },t^{\prime })}\right)\right)-\\ \; & \frac{1}{3}max\left\{Pl_{B(k_{s^{\prime }})}\left(u_{i},r_{i(s^{\prime },t^{\prime })}\right),Pl_{Y(l_{t^{\prime }})}\left(u_{i},r_{i(s^{\prime },t^{\prime })}\right)\right\}] \end{array}$$

*and*

$$\begin{array}{cc} {}[Bel_{g(k_{s^{\prime }},l_{t^{\prime }})}\left(u_{i},r_{i(s^{\prime },t^{\prime })}\right),Pl_{g(k_{s^{\prime }},l_{t^{\prime }})} & (u_{i},r_{i(s^{\prime },t^{\prime })})]=\\ \; & [\frac{2}{3}\left(Bel_{b(k_{s^{\prime }})}\left(u_{i},r_{i(s^{\prime },t^{\prime })}\right)+Bel_{y(l_{t^{\prime }})}\left(u_{i},r_{i(s^{\prime },t^{\prime })}\right)\right)-\\ \; & \frac{1}{3}max\left\{Bel_{b(k_{s^{\prime }})}\left(u_{i},r_{i(s^{\prime },t^{\prime })}\right),Bel_{y(l_{t^{\prime }})}\left(u_{i},r_{i(s^{\prime },t^{\prime })}\right)\right\},\\ \; & \frac{2}{3}\left(Pl_{b(k_{s^{\prime }})}\left(u_{i},r_{i(s^{\prime },t^{\prime })}\right)+Pl_{y(l_{t^{\prime }})}\left(u_{i},r_{i(s^{\prime },t^{\prime })}\right)\right)-\\ \; & \frac{1}{3}max\left\{Pl_{b(k_{s^{\prime }})}\left(u_{i},r_{i(s^{\prime },t^{\prime })}\right),Pl_{y(l_{t^{\prime }})}\left(u_{i},r_{i(s^{\prime },t^{\prime })}\right)\right\}] \end{array}$$

*with $\left(u_{i},r_{i(s^{\prime },t^{\prime })}^{1}\right)\in B_{b}\left(K\right)$ and $\left(u_{i},r_{i(s^{\prime },t^{\prime })}^{2}\right)\in Y_{y}\left(L\right)$.*

**Definition** **26.** *Soft min-OR operation of two PBIVNSS$\left(B_{b},K,M\right)$ and $\left(Y_{y},L,N\right)$ (where $B_{b}:K\to BI^{U\times R}\times BI^{U\times R}$ and $Y_{y}:L\to BI^{U\times R}\times BI^{U\times R})$ defined as:*

$$\left(B_{b},K,M\right)\;\hat{\vee }\;\left(Y_{y},L,N\right)=\left(Q_{q},K\times L,O\right),$$

*where $Q_{q}:K\times L\to BI^{U\times R}\times BI^{U\times R}\;;\;\forall \left(k_{s^{\prime }},l_{t^{\prime }}\right)\in \left(K\times L\right),\;s^{\prime },t^{\prime }\in \Lambda ,\;and\;O=min\left(M,N\right),$*

$$\begin{array}{cc} Q_{q}\left(k_{s^{\prime }},l_{t^{\prime }}\right)\in & \frac{\left(u_{i},r_{i(s^{\prime },t^{\prime })}\right)}{BI_{Q(k_{s^{\prime }},l_{t^{\prime }})}\left(u_{i},r_{i(s^{\prime },t^{\prime })}\right),BI_{q(k_{s^{\prime }},l_{t^{\prime }})}\left(u_{i},r_{i(s^{\prime },t^{\prime })}\right)}\Leftrightarrow r_{i(s^{\prime },t^{\prime })}=min\left(r_{i(s^{\prime },t^{\prime })}^{1},r_{i(s^{\prime },t^{\prime })}^{2}\right),\\ \; & \;\;\;\;\;BI_{Q(k_{s^{\prime }},l_{t^{\prime }})}\left(u_{i},r_{i(s^{\prime },t^{\prime })}\right)=\left[Bel_{Q(k_{s^{\prime }},l_{t^{\prime }})}\left(u_{i},r_{i(s^{\prime },t^{\prime })}\right),Pl_{Q(k_{s^{\prime }},l_{t^{\prime }})}\left(u_{i},r_{i(s^{\prime },t^{\prime })}\right)\right],\\ \; & \;\;\;\;\;BI_{q(k_{s^{\prime }},l_{t^{\prime }})}\left(u_{i},r_{i(s^{\prime },t^{\prime })}\right)=\left[Bel_{q(k_{s^{\prime }},l_{t^{\prime }})}\left(u_{i},r_{i(s^{\prime },t^{\prime })}\right),Pl_{q(k_{s^{\prime }},l_{t^{\prime }})}\left(u_{i},r_{i(s^{\prime },t^{\prime })}\right)\right]. \end{array}$$

*where,*

$$\begin{array}{cc} {}[Bel_{Q(k_{s^{\prime }},l_{t^{\prime }})}\left(u_{i},r_{i(s^{\prime },t^{\prime })}\right), & Pl_{Q(k_{s^{\prime }},l_{t^{\prime }})}\left(u_{i},r_{i(s^{\prime },t^{\prime })}\right)]=\\ \; & [\frac{2}{3}\left(Bel_{B(k_{s^{\prime }})}\left(u_{i},r_{i(s^{\prime },t^{\prime })}\right)+Bel_{Y(l_{t^{\prime }})}\left(u_{i},r_{i(s^{\prime },t^{\prime })}\right)\right)-\\ \; & \frac{1}{3}min\left\{Bel_{B(k_{s^{\prime }})}\left(u_{i},r_{i(s^{\prime },t^{\prime })}\right),Bel_{Y(l_{t^{\prime }})}\left(u_{i},r_{i(s^{\prime },t^{\prime })}\right)\right\},\\ \; & \frac{2}{3}\left(Pl_{B(k_{s^{\prime }})}\left(u_{i},r_{i(s^{\prime },t^{\prime })}\right)+Pl_{Y(l_{t^{\prime }})}\left(u_{i},r_{i(s^{\prime },t^{\prime })}\right)\right)-\\ \; & \frac{1}{3}min\left\{Pl_{B(k_{s^{\prime }})}\left(u_{i},r_{i(s^{\prime },t^{\prime })}\right),Pl_{Y(l_{t^{\prime }})}\left(u_{i},r_{i(s^{\prime },t^{\prime })}\right)\right\}] \end{array}$$

*and*

$$\begin{array}{cc} {}[Bel_{q(k_{s^{\prime }},l_{t^{\prime }})}\left(u_{i},r_{i(s^{\prime },t^{\prime })}\right),Pl_{q(k_{s^{\prime }},l_{t^{\prime }})} & (u_{i},r_{i(s^{\prime },t^{\prime })})]=\\ \; & [\frac{2}{3}\left(Bel_{b(k_{s^{\prime }})}\left(u_{i},r_{i(s^{\prime },t^{\prime })}\right)+Bel_{y(l_{t^{\prime }})}\left(u_{i},r_{i(s^{\prime },t^{\prime })}\right)\right)-\\ \; & \frac{1}{3}min\left\{Bel_{b(k_{s^{\prime }})}\left(u_{i},r_{i(s^{\prime },t^{\prime })}\right),Bel_{y(l_{t^{\prime }})}\left(u_{i},r_{i(s^{\prime },t^{\prime })}\right)\right\},\\ \; & \frac{2}{3}\left(Pl_{b(k_{s^{\prime }})}\left(u_{i},r_{i(s^{\prime },t^{\prime })}\right)+Pl_{y(l_{t^{\prime }})}\left(u_{i},r_{i(s^{\prime },t^{\prime })}\right)\right)-\\ \; & \frac{1}{3}min\left\{Pl_{b(k_{s^{\prime }})}\left(u_{i},r_{i(s^{\prime },t^{\prime })}\right),Pl_{y(l_{t^{\prime }})}\left(u_{i},r_{i(s^{\prime },t^{\prime })}\right)\right\}] \end{array}$$

*with $\left(u_{i},r_{i(s^{\prime },t^{\prime })}^{1}\right)\in B_{b}\left(K\right)$ and $\left(u_{i},r_{i(s^{\prime },t^{\prime })}^{2}\right)\in Y_{y}\left(L\right)$.*

**Example** **10.** *Consider $\left(B_{b},K,7\right)$ and $\left(Y_{y},L,6\right)$ as described in Example 8, then their soft max-AND is:*

$$\begin{array}{c} \begin{array}{cc} (G_{g},K\times L,7)=\{ & \left(\left(k_{1},k_{2}\right),\left(\frac{\left(u_{1},2\right)}{\left[0.23,0.66],[0.33,0.56\right]},\frac{\left(u_{2},5\right)}{\left[0.23,0.43],[0.10,0.56\right]},\frac{\left(u_{3},4\right)}{\left[0.26,0.63],[0.30,0.56\right]}\right)\right),\\ & \left(\left(k_{1},k_{4}\right),\left(\frac{\left(u_{1},1\right)}{\left[0.40,0.83],[0.36,0.73\right]},\frac{\left(u_{2},5\right)}{\left[0.36,0.50],[0.20,0.60\right]},\frac{\left(u_{3},3\right)}{\left[0.33,0.70],[0.16,0.63\right]}\right)\right),\\ & \left(\left(k_{2},k_{2}\right),\left(\frac{\left(u_{1},4\right)}{\left[0.33,0.63],[0.43,0.56\right]},\frac{\left(u_{2},1\right)}{\left[0.13,0.60],[0.30,0.76\right]},\frac{\left(u_{3},6\right)}{\left[0.33,0.73],[0.33,0.70\right]}\right)\right),\\ & \left(\left(k_{2},k_{4}\right),\left(\frac{\left(u_{1},4\right)}{\left[0.60,0.76],[0.50,0.73\right]},\frac{\left(u_{2},5\right)}{\left[0.23,0.73],[0.50,0.83\right]},\frac{\left(u_{3},6\right)}{\left[0.40,0.83],[0.20,0.76\right]}\right)\right),\\ & \left(\left(k_{3},k_{2}\right),\left(\frac{\left(u_{1},6\right)}{\left[0.13,0.33],[0.40,0.50\right]},\frac{\left(u_{2},3\right)}{\left[0.40,0.63],[0.30,0.73\right]},\frac{\left(u_{3},5\right)}{\left[0.40,0.76],[0.30,0.63\right]}\right)\right),\\ & \left(\left(k_{3},k_{4}\right),\left(\frac{\left(u_{1},6\right)}{\left[0.26,0.43],[0.43,0.60\right]},\frac{\left(u_{2},5\right)}{\left[0.60,0.76],[0.50,0.80\right]},\frac{\left(u_{3},5\right)}{\left[0.46,0.90],[0.16,0.70\right]}\right)\right)\}. \end{array} \end{array}$$

*their soft min-OR is:*

$$\begin{array}{c} \begin{array}{cc} (Q_{q},K\times L,6)=\{ & \left(\left(k_{1},k_{2}\right),\left(\frac{\left(u_{1},1\right)}{\left[0.26,0.73],[0.36,0.63\right]},\frac{\left(u_{2},1\right)}{\left[0.26,0.46],[0.10,0.63\right]},\frac{\left(u_{3},2\right)}{\left[0.33,0.66],[0.30,0.63\right]}\right)\right),\\ & \left(\left(k_{1},k_{4}\right),\left(\frac{\left(u_{1},0\right)}{\left[0.50,0.86],[0.43,0.76\right]},\frac{\left(u_{2},5\right)}{\left[0.43,0.60],[0.30,0.70\right]},\frac{\left(u_{3},2\right)}{\left[0.36,0.80],[0.23,0.76\right]}\right)\right),\\ & \left(\left(k_{2},k_{2}\right),\left(\frac{\left(u_{1},2\right)}{\left[0.46,0.66],[0.46,0.63\right]},\frac{\left(u_{2},0\right)}{\left[0.16,0.70],[0.50,0.83\right]},\frac{\left(u_{3},4\right)}{\left[0.46,0.76],[0.36,0.70\right]}\right)\right),\\ & \left(\left(k_{2},k_{4}\right),\left(\frac{\left(u_{1},0\right)}{\left[0.60,0.83],[0.50,0.76\right]},\frac{\left(u_{2},0\right)}{\left[0.36,0.76],[0.60,0.86\right]},\frac{\left(u_{3},3\right)}{\left[0.50,0.86],[0.30,0.83\right]}\right)\right),\\ & \left(\left(k_{3},k_{2}\right),\left(\frac{\left(u_{1},2\right)}{\left[0.16,0.46],[0.40,0.50\right]},\frac{\left(u_{2},1\right)}{\left[0.60,0.76],[0.50,0.76\right]},\frac{\left(u_{3},4\right)}{\left[0.60,0.83],[0.30,0.66\right]}\right)\right),\\ & \left(\left(k_{3},k_{4}\right),\left(\frac{\left(u_{1},0\right)}{\left[0.43,0.66],[0.46,0.70\right]},\frac{\left(u_{2},3\right)}{\left[0.70,0.83],[0.60,0.80\right]},\frac{\left(u_{3},3\right)}{\left[0.63,0.90],[0.23,0.80\right]}\right)\right)\}. \end{array} \end{array}$$

**Proposition** **3.** *Given that $\left(B_{b},K,M\right),\left(Y_{y},L,N\right),$ and $\left(D_{d},T,O\right)$ are any three BIVNSS on U, then the commutative and associative properties hold:*

*(1)*  *$\left(B_{b},K,M\right){⊓}_{E}\left(B_{b},K,M\right)=\left(B_{b},K,M\right)$,*
*(2)*  *$\left(B_{b},K,M\right){⊓}_{R}\left(B_{b},K,M\right)=\left(B_{b},K,M\right)$,*
*(3)*  *$\left(B_{b},K,M\right){⊔}_{E}\left(B_{b},K,M\right)=\left(B_{b},K,M\right)$,*
*(4)*  *$\left(B_{b},K,M\right){⊔}_{R}\left(B_{b},K,M\right)=\left(B_{b},K,M\right)$,*
*(5)*  *$\left(B_{b},K,M\right){⊓}_{E}\left(Y_{y},L,N\right)=\left(Y_{y},L,N\right){⊓}_{E}\left(B_{b},K,M\right)$,*
*(6)*  *$\left(B_{b},K,M\right){⊓}_{R}\left(Y_{y},L,N\right)=\left(Y_{y},L,N\right){⊓}_{R}\left(B_{b},K,M\right)$,*
*(7)*  *$\left(B_{b},K,M\right){⊔}_{E}\left(Y_{y},L,N\right)=\left(Y_{y},L,N\right){⊔}_{E}\left(B_{b},K,M\right)$,*
*(8)*  *$\left(B_{b},K,M\right){⊔}_{R}\left(Y_{y},L,N\right)=\left(Y_{y},L,N\right){⊔}_{R}\left(B_{b},K,M\right)$,*
*(9)*  *$\left(B_{b},K,M\right){⊓}_{E}\left(\left(Y_{y},L,N\right){⊓}_{E}\left(D_{d},T,O\right)\right)=\left(\left(B_{b},K,M\right){⊓}_{E}\left(Y_{y},L,N\right)\right){⊓}_{E}\left(D_{d},T,O\right)$,*
*(10)*  *$\left(B_{b},K,M\right){⊓}_{R}\left(\left(Y_{y},L,N\right){⊓}_{R}\left(D_{d},T,O\right)\right)=\left(\left(B_{b},K,M\right){⊓}_{R}\left(Y_{y},L,N\right)\right){⊓}_{R}\left(D_{d},T,O\right)$,*
*(11)*  *$\left(B_{b},K,M\right){⊔}_{E}\left(\left(Y_{y},L,N\right){⊔}_{E}\left(D_{d},T,O\right)\right)=\left(\left(B_{b},K,M\right){⊔}_{E}\left(Y_{y},L,N\right)\right){⊔}_{E}\left(D_{d},T,O\right)$,*
*(12)*  *$\left(B_{b},K,M\right){⊔}_{R}\left(\left(Y_{y},L,N\right){⊔}_{R}\left(D_{d},T,O\right)\right)=\left(\left(B_{b},K,M\right){⊔}_{R}\left(Y_{y},L,N\right)\right){⊔}_{R}\left(D_{d},T,O\right)$.*

**Proof.**  (1)–(8) follows from definition. For (9), let $\left(Y_{y},L,N\right)$ and $\left(D_{d},T,O\right)$ (where $Y_{y}:L\to BI^{U\times R}\times BI^{U\times R}$ and $D_{d}:T\to BI^{U\times R}\times BI^{U\times R})$ be two PBIVNSS by the definition of extended intersection we have $\left(S_{s},V,Z\right)\;\left(where\;S_{s}:V\to BI^{U\times R}\times BI^{U\times R}\right)$ such that $\left(S_{s},V,Z\right)=\left(Y_{y},L,N\right)\cap_{E}\left(D_{d},T,O\right)$, where $S_{s}=Y_{y}{⊓}_{E}D_{d},\;V=L\cup T,\;Z=max\left(N,O\right)\;;\;\forall v_{j}\in V$ with $v_{j}^{1}\in L$ and $v_{j}^{2}\in T$,

$$\;S_{s}\left(v_{j}\right)=\left\{\begin{array}{cc} \;Y_{y}\left(v_{j}^{1}\right) & ,\;\mathrm{if}\;\;v_{j}\in L-T,\\ \;D_{d}\left(v_{j}^{2}\right) & ,\;\mathrm{if}\;\;v_{j}\in T-L,\\ \;Y_{y}\left(v_{j}^{1}\right){⊓}_{R}D_{d}\left(v_{j}^{2}\right) & ,\;\;\mathrm{if}\;\;v_{j}\in L\cap T. \end{array}\right.$$

As, $\left(B_{b},K,M\right){⊓}_{E}\left(\left(Y_{y},L,N\right){⊓}_{E}\left(D_{d},T,O\right)\right)=\left(B_{b},K,M\right){⊓}_{E}\left(S_{s},V,Z\right)$. Suppose $\left(B_{b},K,M\right)$${⊓}_{E}\left(S_{s},V,Z\right)=\left(G_{g},W,X\right)$ such that $G_{g}:W\to BI^{U\times R}\times BI^{U\times R},$ where $G_{g}=B_{b}{⊓}_{E}S_{s},\;W=K\cup V=K\cup L\cup T,\;X=max\left(M,Z\right)\;;\;\forall w_{j}\in W$ with $w_{j}^{1}\in K$, $w_{j}^{2}\in L$ and

$$w_{j}^{3}\in T,\;G_{g}\left(w_{j}\right)=\left\{\begin{array}{cc} \;B_{b}\left(w_{j}^{1}\right) & ,\;\mathrm{if}\;\;w_{j}\in K-L-T,\\ \;Y_{y}\left(w_{j}^{2}\right) & ,\;\mathrm{if}\;\;w_{j}\in L-K-T,\\ \;D_{d}\left(w_{j}^{3}\right) & ,\;\mathrm{if}\;\;w_{j}\in T-K-L,\\ \;Y_{y}\left(w_{j}^{2}\right){⊓}_{R}D_{d}\left(w_{j}^{3}\right) & ,\;\mathrm{if}\;\;w_{j}\in L\cap T-K,\\ \;B_{b}\left(w_{j}^{1}\right){⊓}_{R}Y_{y}\left(w_{j}^{2}\right) & ,\;\mathrm{if}\;\;w_{j}\in K\cap L-T,\\ \;B_{b}\left(w_{j}^{1}\right){⊓}_{R}D_{d}\left(w_{j}^{3}\right) & ,\;\mathrm{if}\;\;w_{j}\in K\cap T-L,\\ \;B_{b}\left(w_{j}^{1}\right){⊓}_{R}Y_{y}\left(w_{j}^{2}\right){⊓}_{R}D_{d}\left(w_{j}^{3}\right) & ,\;\;\mathrm{if}\;\;w_{j}\in K\cap L\cap T. \end{array}\right.$$

Again, let $\left(B_{b},K,M\right)$ and $\left(Y_{y},L,N\right)$ (where $B_{b}:K\to BI^{U\times R}\times BI^{U\times R}$ and $Y_{y}:L\to BI^{U\times R}\times BI^{U\times R})$ be two PBIVNSS by the definition of extended intersection we have $\left(F_{f},S,P\right)\left(where\;F_{f}:S\to BI^{U\times R}\times BI^{U\times R}\right)$ such that $\left(F_{f},S,P\right)=\left(B_{b},K,M\right)\cap_{E}\left(Y_{y},L,N\right)$, where $F_{f}=B_{b}{⊓}_{E}Y_{y},\;S=K\cup L,\;P=max\left(M,N\right)\;;\;\forall s_{j}\in S$ with $s_{j}^{1}\in K$ and $s_{j}^{2}\in L$,

$$\;F_{f}\left(s_{j}\right)=\left\{\begin{array}{cc} \;B_{b}\left(s_{j}^{1}\right) & ,\;\mathrm{if}\;\;s_{j}\in K-L,\\ \;Y_{y}\left(s_{j}^{2}\right) & ,\;\mathrm{if}\;\;s_{j}\in L-K,\\ \;B_{b}\left(s_{j}^{1}\right){⊓}_{R}Y_{y}\left(s_{j}^{2}\right) & ,\;\;\mathrm{if}\;\;s_{j}\in K\cap L. \end{array}\right.$$

As, $\left(\left(B_{b},K,M\right){⊓}_{E}\left(Y_{y},L,N\right)\right){⊓}_{E}\left(D_{d},T,O\right)=\left(F_{f},S,P\right){⊓}_{E}\left(D_{d},T,O\right)$. Suppose $\left(F_{f},S,P\right){⊓}_{E}$$\left(D_{d},T,O\right)=\left(I_{i},W,X\right)$ such that $I_{i}:W\to BI^{U\times R}\times BI^{U\times R}$, where $I_{i}=F_{f}{⊓}_{E}D_{d},\;W=S\cup T=K\cup L\cup T,\;X=max\left(P,O\right)\;;\;\forall w_{j}\in W$ with $w_{j}^{1}\in K$, $w_{j}^{2}\in L$ and $w_{j}^{3}\in T$,

$$I_{i}\left(w_{j}\right)=\left\{\begin{array}{cc} \;B_{b}\left(w_{j}^{1}\right) & ,\;\mathrm{if}\;\;w_{j}\in K-L-T,\\ \;y_{y}\left(w_{j}^{2}\right) & ,\;\mathrm{if}\;\;w_{j}\in L-K-T,\\ \;D_{d}\left(w_{j}^{3}\right) & ,\;\mathrm{if}\;\;w_{j}\in T-K-L,\\ \;Y_{y}\left(w_{j}^{2}\right){⊓}_{R}D_{d}\left(w_{j}^{3}\right) & ,\;\mathrm{if}\;\;w_{j}\in L\cap T-K,\\ \;B_{b}\left(w_{j}^{1}\right){⊓}_{R}Y_{y}\left(w_{j}^{2}\right) & ,\;\mathrm{if}\;\;w_{j}\in K\cap L-T,\\ \;B_{b}\left(w_{j}^{1}\right){⊓}_{R}D_{d}\left(w_{j}^{3}\right) & ,\;\mathrm{if}\;\;w_{j}\in K\cap T-L,\\ \;B_{b}\left(w_{j}^{1}\right){⊓}_{R}Y_{y}\left(w_{j}^{2}\right){⊓}_{R}D_{d}\left(w_{j}^{3}\right) & ,\;\;\mathrm{if}\;\;w_{j}\in K\cap L\cap T. \end{array}\right.$$

Then, $G_{g}\left(w_{j}\right)=I_{i}\left(w_{j}\right),\;\forall w_{j}\in W$. Hence, (9) is hwld and (10)–(12) are similar to (9).    □

**Proposition** **4.** *Given that $\left(B_{b},K,M\right)$ and $\left(Y_{y},L,N\right)$ are any two PBIVNSS on U, then the following results hold:*

*(1)*  *$\left({\left(B^{c}\right)}_{{\left(b^{c}\right)}^{c}}^{c},K,M\right)=\left(B_{b},K,M\right)$,*
*(2)*  *$\left(B_{b^{c}}^{c},K,M\right){⊔}_{E}\left(Y_{y^{c}}^{c},L,N\right)=\left({\left(B_{b}{⊓}_{E}Y_{y}\right)}^{c},\left(K\cup L\right),max\left(M,N\right)\right)$,*
*(3)*  *$\left(B_{b^{c}}^{c},K,M\right){⊓}_{E}\left(Y_{y^{c}}^{c},L,N\right)=\left({\left(B_{b}{⊔}_{E}Y_{y}\right)}^{c},\left(K\cup L\right),max\left(M,N\right)\right)$,*
*(4)*  *$\left(B_{b^{c}}^{c},K,M\right){⊔}_{R}\left(Y_{y^{c}}^{c},L,N\right)=\left({\left(B_{b}{⊓}_{R}Y_{y}\right)}^{c},\left(K\cap L\right),max\left(M,N\right)\right)$,*
*(5)*  *$\left(B_{b^{c}}^{c},K,M\right){⊓}_{R}\left(Y_{y^{c}}^{c},L,N\right)=\left({\left(B_{b}{⊔}_{R}Y_{y}\right)}^{c},\left(K\cap L\right),min\left(M,N\right)\right)$.*

**Proof.**  (1) is straight-forward. We start from (2). Let $\left(B_{b},K,M\right)$ and $\left(Y_{y},L,N\right)$ (where $B_{b}:K\to BI^{U\times R}\times BI^{U\times R}$ and $Y_{y}:L\to BI^{U\times R}\times BI^{U\times R})$ be two PBIVNSS on *U*. Then by the definition of weak possibility belief interval-valued complement and extended union we have,

$$\left(B_{b^{c}}^{c},K,M\right){⊔}_{E}\left(Y_{y^{c}}^{c},L,N\right)=\left(J_{j},S,P\right),$$

where $J_{j}=B_{b^{c}}^{c}{⊔}_{E}Y_{y^{c}}^{c},\;S=K\cup L,\;P=max\left(M,N\right);\forall s_{j}\in S$ with $s_{j}^{1}\in K$ and $s_{j}^{2}\in L$,

$$\;J_{j}\left(s_{j}\right)=\left\{\begin{array}{cc} \;B_{b^{c}}^{c}\left(s_{j}^{1}\right) & ,\;\mathrm{if}\;\;s_{j}\in K-L,\\ \;Y_{y^{c}}^{c}\left(s_{j}^{2}\right) & ,\;\mathrm{if}\;\;s_{j}\in L-K,\\ \;B_{b^{c}}^{c}\left(s_{j}^{1}\right){⊔}_{E}Y_{y^{c}}^{c}\left(s_{j}^{2}\right) & ,\;\;\mathrm{if}\;\;s_{j}\in K\cap L. \end{array}\right.$$

Again by the definition of extended intersection we have $\left(F_{f},S,P\right)=\left(B_{b}\cap_{E}Y_{y}\right),\;S=K\cup L,\;P=max\left(M,N\right);\forall s_{j}\in S$ with $s_{j}^{1}\in K$ and $s_{j}^{2}\in L$,

$$\;F_{f}\left(s_{j}\right)=\left\{\begin{array}{cc} \;B_{b}\left(s_{j}^{1}\right) & ,\;\mathrm{if}\;\;s_{j}\in K-L,\\ \;y_{y}\left(s_{j}^{2}\right) & ,\;\mathrm{if}\;\;s_{j}\in L-K,\\ \;B_{b}\left(s_{j}^{1}\right){⊓}_{E}Y_{y}\left(s_{j}^{2}\right) & ,\;\;\mathrm{if}\;\;s_{j}\in K\cap L. \end{array}\right.$$

Thus, by the definition of weak possibility belief interval-valued complement we have,

$$\;F_{f^{c}}^{c}\left(s_{j}\right)=\left\{\begin{array}{cc} \;B_{b^{c}}^{c}\left(s_{j}^{1}\right) & ,\;\mathrm{if}\;\;s_{j}\in K-L,\\ \;Y_{y^{c}}^{c}\left(s_{j}^{2}\right) & ,\;\mathrm{if}\;\;s_{j}\in L-K,\\ \;B_{b^{c}}^{c}\left(s_{j}^{1}\right){⊔}_{E}Y_{y^{c}}^{c}\left(s_{j}^{2}\right) & ,\;\;\mathrm{if}\;\;s_{j}\in K\cap L. \end{array}\right.$$

Then, $J_{j}\left(s_{j}\right)=F_{f^{c}}^{c}\left(s_{j}\right);\;\forall s_{j}\in S$. Hence, (2) holds. Remaining (3)–(5) are similar to (2).    □

**Figure 1:**
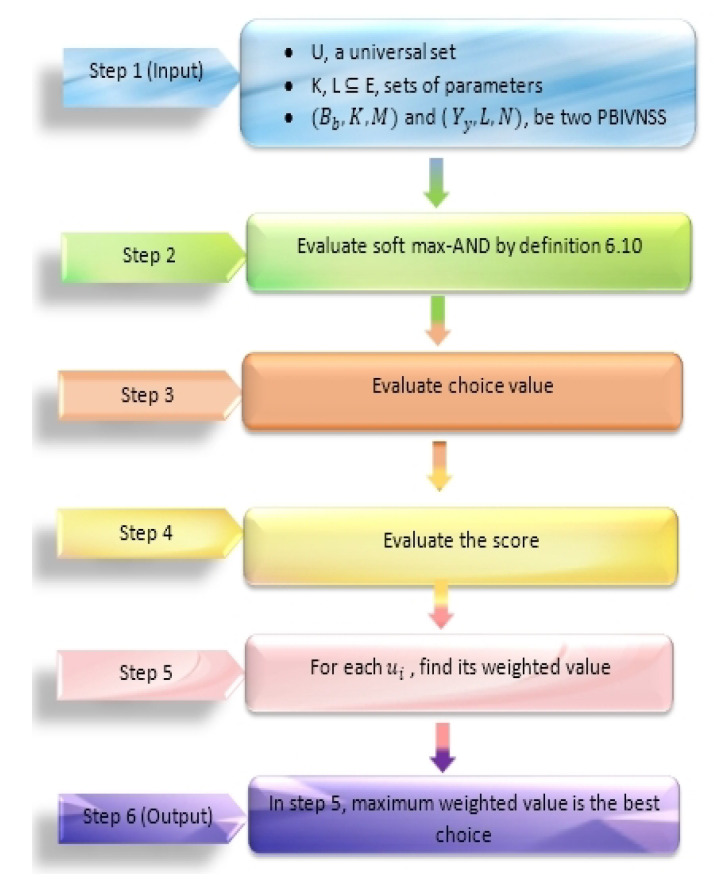
Flow chart of Algorithm 1.

**Figure 2:**
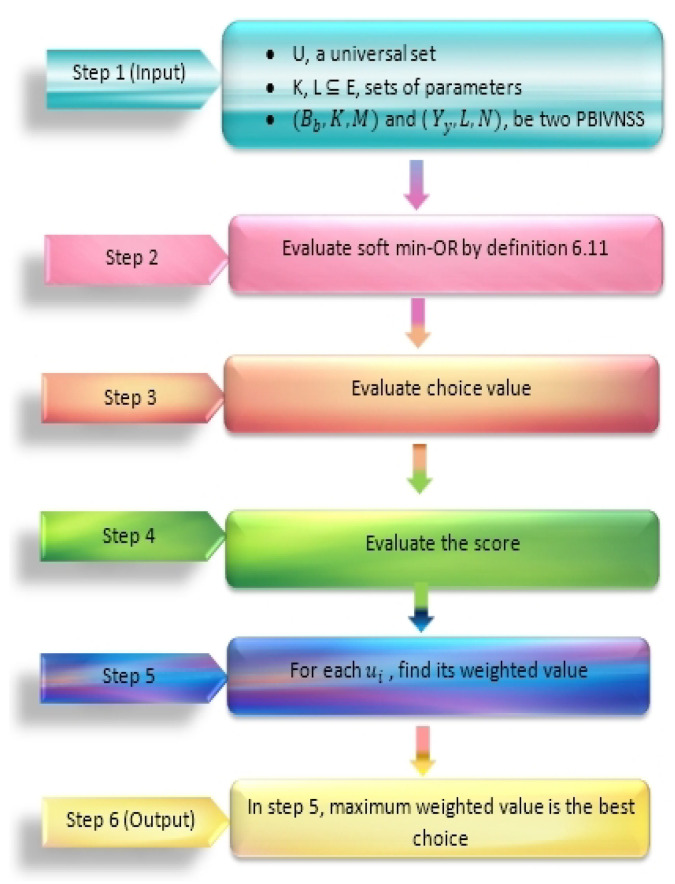
Flow chart of Algorithm 2.

In Figure 1 and Figure 2, we give the flow charts of Algorithms 1 and 2 respectively.

## 7. Algorithms

In this section, we will present the algorithms on soft max-AND and soft min-OR operations on two possibility belief interval valued N-soft sets for decision making.
**Algorithm 1** Soft max-AND operations**Step 1:** We have two $PBIVNSS\;(B_{b},K,M)$ and $\left(Y_{y},L,N\right)$ (where $B_{b}:K\to BI^{U\times R}\times BI^{U\times R}$ and $Y_{y}:L\to BI^{U\times R}\times BI^{U\times R})$ on universal set $U=\{u_{1},u_{2},\ldots ,u_{l}\}$.**Step 2:** Evaluate
$$\left(G_{g},K\times L,P\right)=\left(B_{b},K,M\right)\hat{\wedge }\left(Y_{y},L,N\right),$$
   where
$$G_{g}:K\times L\to BI^{U\times R}\times BI^{U\times R}\;;\;\forall \left(k_{s^{\prime }},l_{t^{\prime }}\right)\in \left(K\times L\right),\;s^{\prime },t^{\prime }\in \Lambda \;and\;P=max\left(M,N\right).$$**Step 3:** Evaluate the choice value $C\left(k_{s^{\prime }},l_{t^{\prime }}\right)\left(u_{i}\right);\;\forall \;u_{i}\in U,\left(k_{s^{\prime }},l_{t^{\prime }}\right)\in \left(K\times L\right),s^{\prime },t^{\prime }\in \Lambda$ defined as:$$C\left(k_{s^{\prime }},l_{t^{\prime }}\right)\left(u_{i}\right)=\left\{\frac{r_{i(s^{\prime },t^{\prime })}}{I_{C(k_{s^{\prime }},l_{t^{\prime }})}\left(u_{i},r_{i(s^{\prime },t^{\prime })}\right)}\right\},$$
   where the interval of choice value is:$$\begin{array}{c} \begin{array}{cc} I_{C(k_{s^{\prime }},l_{t^{\prime }})}\left(u_{i},r_{i(s^{\prime },t^{\prime })}\right)= & [Bel_{G(k_{s^{\prime }},l_{t^{\prime }})}\left(u_{i},r_{i(s^{\prime },t^{\prime })}\right)+Bel_{g(k_{s^{\prime }},l_{t^{\prime }})}\left(u_{i},r_{i(s^{\prime },t^{\prime })}\right),\\ & \;\;\;\;\;Pl_{G(k_{s^{\prime }},l_{t^{\prime }})}\left(u_{i},r_{i(s^{\prime },t^{\prime })}\right)+Pl_{g(k_{s^{\prime }},l_{t^{\prime }})}\left(u_{i},r_{i(s^{\prime },t^{\prime })}\right)]. \end{array} \end{array}$$**Step 4:** Evaluate the score $S\left(k_{s^{\prime }},l_{t^{\prime }}\right)\left(u_{i}\right);\;\forall \;u_{i}\in U,\left(k_{s^{\prime }},l_{t^{\prime }}\right)\in \left(K\times L\right),\;s^{\prime },t^{\prime }\in \Lambda$ defined as:$$S\left(k_{s^{\prime }},l_{t^{\prime }}\right)\left(u_{i}\right)=S_{1}\left(k_{s^{\prime }},l_{t^{\prime }}\right)\left(u_{i}\right)\times S_{2}\left(k_{s^{\prime }},l_{t^{\prime }}\right)\left(u_{i}\right),$$
   where,
$$\begin{array}{c} \begin{array}{cc} S_{1}\left(k_{s^{\prime }},l_{t^{\prime }}\right)\left(u_{i}\right)=\sum_{t_{q}\in U}[ & \alpha_{1}I_{C(k_{s^{\prime }},l_{t^{\prime }})}\left(u_{i},r_{i(s^{\prime },t^{\prime })}\right)-\alpha_{1}I_{C(k_{s^{\prime }},l_{t^{\prime }})}\left(t_{q},r_{q(s^{\prime },t^{\prime })}\right)+\\ & \alpha_{2}I_{C(k_{s^{\prime }},l_{t^{\prime }})}\left(u_{i},r_{i(s^{\prime },t^{\prime })}\right)-\alpha_{2}I_{C(k_{s^{\prime }},l_{t^{\prime }})}\left(t_{q},r_{q(s^{\prime },t^{\prime })}\right)]. \end{array} \end{array}$$
   where $\alpha_{m}:\left[0,1\right]\to \left[0,1\right]$ is the mth projection mapping such that $\alpha_{1}$ is the lower membership value and $\alpha_{2}$ is the upper membership value of choice interval and
$$S_{2}\left(k_{s^{\prime }},l_{t^{\prime }}\right)\left(u_{i}\right)=\sum_{q=1}^{l}max\left\{r_{i(s^{\prime },t^{\prime })},r_{q(s^{\prime },t^{\prime })}\right\}.$$**Step 5:** For each $S(u_{l})$ it’s weighted value is:$$S\left(u_{l}\right)=\sum_{\left(k_{s^{\prime }},l_{t^{\prime }}\right)\in \left(K\times L\right)}S\left(k_{s^{\prime }},l_{t^{\prime }}\right)\left(u_{l}\right).$$**Step 6:** Now the optimal decision is:$$X=arg\;max\;\left\{S\left(u_{1}\right),S\left(u_{2}\right),cdots,S\left(u_{l}\right)\right\}.$$

**Algorithm 2** Soft min-OR operations
**Step 1:** Let we have two $PBIVNSS\;(B_{b},K,M)$ and $\left(Y_{y},L,N\right)$ (where $B_{b}:K\to BI^{U\times R}\times BI^{U\times R}$ and $Y_{y}:L\to BI^{U\times R}\times BI^{U\times R})$ on universal set $U=\{u_{1},u_{2},\ldots ,u_{l}\}$.
**Step 2:** Evaluate
$$\left(Q_{q},K\times L,P\right)=\left(B_{b},K,M\right)\hat{\vee }\left(Y_{y},L,N\right),$$
   where
$$Q_{q}:K\times L\to BI^{U\times R}\times BI^{U\times R}\;;\;\forall \left(k_{s^{\prime }},l_{t^{\prime }}\right)\in \left(K\times L\right),$$


$\;s^{\prime },t^{\prime }\in \Lambda \;and\;O=min\left(M,N\right).$


**Step 3:** Evaluate the choice value $C\left(k_{s^{\prime }},l_{t^{\prime }}\right)\left(u_{i}\right);\;\forall \;u_{i}\in U,\left(k_{s^{\prime }},l_{t^{\prime }}\right)\in \left(K\times L\right),s^{\prime },t^{\prime }\in \Lambda$ defined as:$$C\left(k_{s^{\prime }},l_{t^{\prime }}\right)\left(u_{i}\right)=\left\{\frac{r_{i(s^{\prime },t^{\prime })}}{I_{C(k_{s^{\prime }},l_{t^{\prime }})}\left(u_{i},r_{i(s^{\prime },t^{\prime })}\right)}\right\},$$
   where the interval of choice value is:$$\begin{array}{c} \begin{array}{cc} I_{C(k_{s^{\prime }},l_{t^{\prime }})}\left(u_{i},r_{i(s^{\prime },t^{\prime })}\right)= & [Bel_{G(k_{s^{\prime }},l_{t^{\prime }})}\left(u_{i},r_{i(s^{\prime },t^{\prime })}\right)+Bel_{g(k_{s^{\prime }},l_{t^{\prime }})}\left(u_{i},r_{i(s^{\prime },t^{\prime })}\right),\\ & \;\;\;\;\;Pl_{G(k_{s^{\prime }},l_{t^{\prime }})}\left(u_{i},r_{i(s^{\prime },t^{\prime })}\right)+Pl_{g(k_{s^{\prime }},l_{t^{\prime }})}\left(u_{i},r_{i(s^{\prime },t^{\prime })}\right)]. \end{array} \end{array}$$
**Step 4:** Evaluate the score $S\left(k_{s^{\prime }},l_{t^{\prime }}\right)\left(u_{i}\right);\;\forall \;u_{i}\in U,\left(k_{s^{\prime }},l_{t^{\prime }}\right)\in \left(K\times L\right),\;s^{\prime },t^{\prime }\in \Lambda$ defined as:$$S\left(k_{s^{\prime }},l_{t^{\prime }}\right)\left(u_{i}\right)=S_{1}\left(k_{s^{\prime }},l_{t^{\prime }}\right)\left(u_{i}\right)\times S_{2}\left(k_{s^{\prime }},l_{t^{\prime }}\right)\left(u_{i}\right),$$
   where,
$$\begin{array}{c} \begin{array}{cc} S_{1}\left(k_{s^{\prime }},l_{t^{\prime }}\right)\left(u_{i}\right)=\sum_{t_{q}\in U}[ & \alpha_{1}I_{C(k_{s^{\prime }},l_{t^{\prime }})}\left(u_{i},r_{i(s^{\prime },t^{\prime })}\right)-\alpha_{1}I_{C(k_{s^{\prime }},l_{t^{\prime }})}\left(t_{q},r_{q(s^{\prime },t^{\prime })}\right)+\\ & \alpha_{2}I_{C(k_{s^{\prime }},l_{t^{\prime }})}\left(u_{i},r_{i(s^{\prime },t^{\prime })}\right)-\alpha_{2}I_{C(k_{s^{\prime }},l_{t^{\prime }})}\left(t_{q},r_{q(s^{\prime },t^{\prime })}\right)], \end{array} \end{array}$$
   where $\alpha_{m}:\left[0,1\right]\to \left[0,1\right]$ is the mth projection mapping such that $\alpha_{1}$ is the lower membership value and $\alpha_{2}$ is the upper membership value of choice interval and
$$S_{2}\left(k_{s^{\prime }},l_{t^{\prime }}\right)\left(u_{i}\right)=\sum_{q=1}^{l}max\left\{r_{i(s^{\prime },t^{\prime })},r_{q(s^{\prime },t^{\prime })}\right\}.$$
**Step 5:** For each $S(u_{l})$ it’s weighted value is:$$S\left(u_{l}\right)=\sum_{\left(k_{s^{\prime }},l_{t^{\prime }}\right)\in \left(K\times L\right)}S\left(k_{s^{\prime }},l_{t^{\prime }}\right)\left(u_{l}\right).$$
**Step 6:** Now the optimal decision is:$X=arg\;max\;\left\{S\left(u_{1}\right),S\left(u_{2}\right),\ldots ,S\left(u_{l}\right)\right\}.$


## 8. Applications

In this section, we give the application of our proposed sets.

**Example** **11.** *Let Mr.*H*wants to buy a smartphone with particular features, and there are three smartphones that are under consideration. Let*$U=\{u_{1},\;u_{2},\;u_{3},\;u_{4},\;u_{5},\;u_{6}\}$*be the set of smartphones. Let E be the set of parameters (particular features) for the evaluation of smartphone and*K,L⊆E*such that*$K=\left\{k_{1}=Reasonable,\;k_{3}=Expensive\right\}\;and\;L=\left\{k_{4}=High\;resolution,\;k_{5}=Good\;battery\;timing,\;k_{6}=Guest\;mode\right\}$. *Assume that Mr.*H*wants to buy one such smartphone depending on two parameters only. Suppose there are two observations*$\left(B_{b},K,4\right)$*and*$\left(Y_{y},L,7\right)$*by two experts as follows:*

$$\begin{array}{c} \begin{array}{cc} (B_{b},K,4)=\{ & (k_{1},(\frac{\left(u_{1},1\right)}{\left[0.1,0.6],[0.4,0.7\right]},\frac{\left(u_{2},3\right)}{\left[0.8,0.9],[0.3,0.8\right]},\frac{\left(u_{3},1\right)}{\left[0.2,0.4],[0.2,0.7\right]},\\ & \;\;\;\;\;\;\;\;\;\;\;\;\;\frac{\left(u_{4},2\right)}{\left[0.7,0.8],[0.5,0.6\right]},\frac{\left(u_{5},0\right)}{\left[0.5,0.9],[0.7,0.9\right]},\frac{\left(u_{6},3\right)}{\left[0.6,0.8],[0.4,0.6\right]})),\\ & (k_{3},(\frac{\left(u_{1},0\right)}{\left[0.5,0.6],[0.2,0.8\right]},\frac{\left(u_{2},3\right)}{\left[0.4,0.8],[0.7,0.9\right]},\frac{\left(u_{3},2\right)}{\left[0.3,0.4],[0.4,0.5\right]},\\ & \;\;\;\;\;\;\;\;\;\;\;\;\;\frac{\left(u_{4},2\right)}{\left[0.2,0.3],[0.5,0.7\right]},\frac{\left(u_{5},1\right)}{\left[0.3,0.7],[0.4,0.8\right]},\frac{\left(u_{6},1\right)}{\left[0.9,1.0],[0.1,0.7\right]}))\}. \end{array} \end{array}$$

$$\begin{array}{c} \begin{array}{cc} (Y_{y},L,7)=\{ & (k_{4},(\frac{\left(u_{1},3\right)}{\left[0.6,0.9],[0.1,0.4\right]},\frac{\left(u_{2},1\right)}{\left[0.2,0.9],[0.4,0.6\right]},\frac{\left(u_{3},4\right)}{\left[0.7,0.8],[0.1,0.2\right]},\\ & \;\;\;\;\;\;\;\;\;\;\;\;\;\frac{\left(u_{4},1\right)}{\left[0.2,0.9],[0.3,0.5\right]},\frac{\left(u_{5},6\right)}{\left[0.4,0.6],[0.8,1.0\right]},\frac{\left(u_{6},0\right)}{\left[0.1,0.9],[0.3,0.8\right]})),\\ & (k_{5},(\frac{\left(u_{1},0\right)}{\left[0.1,0.7],[0.3,0.9\right]},\frac{\left(u_{2},2\right)}{\left[0.7,0.9],[0.6,0.8\right]},\frac{\left(u_{3},6\right)}{\left[0.5,0.9],[0.1,0.5\right]},\\ & \;\;\;\;\;\;\;\;\;\;\;\;\;\frac{\left(u_{4},5\right)}{\left[0.6,1.0],[0.4,0.5\right]},\frac{\left(u_{5},1\right)}{\left[0.7,0.8],[0.3,0.4\right]},\frac{\left(u_{6},3\right)}{\left[0.6,0.7],[0.6,0.8\right]})),\\ & (k_{6},(\frac{\left(u_{1},1\right)}{\left[0.2,0.8],[0.3,0.4\right]},\frac{\left(u_{2},5\right)}{\left[0.8,0.9],[0.2,0.3\right]},\frac{\left(u_{3},0\right)}{\left[0.6,0.8],[0.4,0.8\right]},\\ & \;\;\;\;\;\;\;\;\;\;\;\;\;\frac{\left(u_{4},6\right)}{\left[0.1,0.4],[0.2,0.3\right]},\frac{\left(u_{5},4\right)}{\left[0.6,0.7],[0.5,0.6\right]},\frac{\left(u_{6},6\right)}{\left[0.2,0.9],[0.4,0.7\right]}))\}. \end{array} \end{array}$$

*Firstly, we will evaluate the soft max-AND operation* $\left(G_{g},K\times L,7\right)=\left(B_{b},K,4\right)\hat{\wedge }\left(Y_{y},L,7\right)$ *by using step 2 of Algorithm 1:*

$$\begin{array}{c} \begin{array}{cc} (G_{g},K\times L,7)=\{ & (\left(k_{1},k_{4}\right),(\frac{\left(u_{1},3\right)}{\left[0.26,0.70],[0.20,0.50\right]},\frac{\left(u_{2},3\right)}{\left[0.40,0.90],[0.33,0.66\right]},\frac{\left(u_{3},4\right)}{\left[0.36,0.53],[0.13,0.36\right]},\\ & \;\;\;\;\;\;\;\;\;\;\;\;\;\;\;\;\;\;\;\;\;\frac{\left(u_{4},2\right)}{\left[0.36,0.83],[0.36,0.53\right]},\frac{\left(u_{5},6\right)}{\left[0.43,0.70],[0.73,0.93\right]},\frac{\left(u_{6},3\right)}{\left[0.26,0.83],[0.33,0.66\right]})),\\ & (\left(k_{1},k_{5}\right),(\frac{\left(u_{1},1\right)}{\left[0.10,0.63],[0.33,0.76\right]},\frac{\left(u_{2},3\right)}{\left[0.73,0.90],[0.40,0.80\right]},\frac{\left(u_{3},6\right)}{\left[0.30,0.56],[0.13,0.56\right]},\\ & \;\;\;\;\;\;\;\;\;\;\;\;\;\;\;\;\;\;\;\;\;\frac{\left(u_{4},5\right)}{\left[0.63,0.86],[0.43,0.53\right]},\frac{\left(u_{5},1\right)}{\left[0.56,0.83],[0.43,0.56\right]},\frac{\left(u_{6},3\right)}{\left[0.60,0.73],[0.46,0.66\right]})),\\ & (\left(k_{1},k_{6}\right),(\frac{\left(u_{1},1\right)}{\left[0.13,0.66],[0.33,0.50\right]},\frac{\left(u_{2},5\right)}{\left[0.80,0.90],[0.23,0.46\right]},\frac{\left(u_{3},1\right)}{\left[0.33,0.53],[0.26,0.73\right]},\\ & \;\;\;\;\;\;\;\;\;\;\;\;\;\;\;\;\;\;\;\;\;\frac{\left(u_{4},6\right)}{\left[0.30,0.53],[0.30,0.40\right]},\frac{\left(u_{5},4\right)}{\left[0.53,0.76],[0.56,0.70\right]},\frac{\left(u_{6},6\right)}{\left[0.33,0.83],[0.40,0.63\right]})),\\ & (\left(k_{3},k_{4}\right),(\frac{\left(u_{1},3\right)}{\left[0.53,0.70],[0.13,0.53\right]},\frac{\left(u_{2},3\right)}{\left[0.26,0.83],[0.50,0.70\right]},\frac{\left(u_{3},4\right)}{\left[0.43,0.53],[0.20,0.30\right]},\\ & \;\;\;\;\;\;\;\;\;\;\;\;\;\;\;\;\;\;\;\;\;\frac{\left(u_{4},2\right)}{\left[0.20,0.50],[0.36,0.56\right]},\frac{\left(u_{5},6\right)}{\left[0.33,0.63],[0.53,0.86\right]},\frac{\left(u_{6},1\right)}{\left[0.36,0.93],[0.16,0.73\right]})),\\ & (\left(k_{3},k_{5}\right),(\frac{\left(u_{1},0\right)}{\left[0.23,0.63],[0.23,0.83\right]},\frac{\left(u_{2},3\right)}{\left[0.50,0.83],[0.63,0.83\right]},\frac{\left(u_{3},6\right)}{\left[0.36,0.56],[0.20,0.50\right]},\\ & \;\;\;\;\;\;\;\;\;\;\;\;\;\;\;\;\;\;\;\;\;\frac{\left(u_{4},5\right)}{\left[0.33,0.53],[0.43,0.56\right]},\frac{\left(u_{5},1\right)}{\left[0.43,0.73],[0.33,0.53\right]},\frac{\left(u_{6},3\right)}{\left[0.70,0.80],[0.26,0.73\right]})),\\ & (\left(k_{3},k_{6}\right),(\frac{\left(u_{1},1\right)}{\left[0.30,0.66],[0.23,0.53\right]},\frac{\left(u_{2},5\right)}{\left[0.53,0.83],[0.36,0.50\right]},\frac{\left(u_{3},2\right)}{\left[0.40,0.53],[0.40,0.60\right]},\\ & \;\;\;\;\;\;\;\;\;\;\;\;\;\;\;\;\;\;\;\;\;\frac{\left(u_{4},6\right)}{\left[0.13,0.33],[0.30,0.43\right]},\frac{\left(u_{5},4\right)}{\left[0.40,0.70],[0.43,0.66\right]},\frac{\left(u_{6},6\right)}{\left[0.43,0.93],[0.20,0.70\right]}))\}. \end{array} \end{array}$$

*Then we will evaluate the choice value* $C\left(k_{s^{\prime }},l_{t^{\prime }}\right)\left(u_{i}\right);\;\forall \;u_{i}\in U,\;\left(k_{s^{\prime }},l_{t^{\prime }}\right)\in \left(K\times L\right),\;\;s^{\prime }=1,3\;and\;t^{\prime }=4,5,6$ *by using step 3 of Algorithm 1:*


$C\left(k_{s^{\prime }},k_{t^{\prime }}\right)\left(u_{i}\right)$


***(Grading, Interval Value)***


$C\left(k_{s^{\prime }},k_{t^{\prime }}\right)\left(u_{i}\right)$


***(Grading, Interval Value)***


$C\left(k_{1},k_{4}\right)\left(u_{1}\right)$

(3, [0.46,1.20])

$C\left(k_{3},k_{4}\right)\left(u_{1}\right)$

(3, [0.66,1.23])

$C\left(k_{1},k_{4}\right)\left(u_{2}\right)$

(3, [0.73,1.56])

$C\left(k_{3},k_{4}\right)\left(u_{2}\right)$

(3, [0.76,1.53])

$C\left(k_{1},k_{4}\right)\left(u_{3}\right)$

(4, [0.50,0.90])

$C\left(k_{3},k_{4}\right)\left(u_{3}\right)$

(4, [0.63,0.83])

$C\left(k_{1},k_{4}\right)\left(u_{4}\right)$

(2, [0.73,1.36])

$C\left(k_{3},k_{4}\right)\left(u_{4}\right)$

(2, [0.56,1.06])

$C\left(k_{1},k_{4}\right)\left(u_{5}\right)$

(6, [1.16,1.63])

$C\left(k_{3},k_{4}\right)\left(u_{5}\right)$

(6, [0.86,1.50])

$C\left(k_{1},k_{4}\right)\left(u_{6}\right)$

(3, [0.60,1.50])

$C\left(k_{3},k_{4}\right)\left(u_{6}\right)$

(1, [0.53,1.66])

$C\left(k_{1},k_{5}\right)\left(u_{1}\right)$

(1, [0.43,1.40])

$C\left(k_{3},k_{5}\right)\left(u_{1}\right)$

(0, [0.46,1.46])

$C\left(k_{1},k_{5}\right)\left(u_{2}\right)$

(3, [1.13,1.70])

$C\left(k_{3},k_{5}\right)\left(u_{2}\right)$

(3, [1.13,1.66])

$C\left(k_{1},k_{5}\right)\left(u_{3}\right)$

(6, [0.43,1.13])

$C\left(k_{3},k_{5}\right)\left(u_{3}\right)$

(6, [0.56,1.06])

$C\left(k_{1},k_{5}\right)\left(u_{4}\right)$

(5, [1.06,1.40])

$C\left(k_{3},k_{5}\right)\left(u_{4}\right)$

(5, [0.76,1.10])

$C\left(k_{1},k_{5}\right)\left(u_{5}\right)$

(1, [1.00,1.40])

$C\left(k_{3},k_{5}\right)\left(u_{5}\right)$

(1, [0.76,1.26])

$C\left(k_{1},k_{5}\right)\left(u_{6}\right)$

(3, [1.06,1.40])

$C\left(k_{3},k_{5}\right)\left(u_{6}\right)$

(3, [0.96,1.53])

$C\left(k_{1},k_{6}\right)\left(u_{1}\right)$

(1, [0.46,1.16])

$C\left(k_{3},k_{6}\right)\left(u_{1}\right)$

(1, [0.53,1.20])

$C\left(k_{1},k_{6}\right)\left(u_{2}\right)$

(5, [1.03,1.36])

$C\left(k_{3},k_{6}\right)\left(u_{2}\right)$

(5, [0.90,1.33])

$C\left(k_{1},k_{6}\right)\left(u_{3}\right)$

(1, [0.60,1.26])

$C\left(k_{3},k_{6}\right)\left(u_{3}\right)$

(2, [0.80,1.13])

$C\left(k_{1},k_{6}\right)\left(u_{4}\right)$

(6, [0.60,0.93])

$C\left(k_{3},k_{6}\right)\left(u_{4}\right)$

(6, [0.43,0.76])

$C\left(k_{1},k_{6}\right)\left(u_{5}\right)$

(4, [1.10,1.46])

$C\left(k_{3},k_{6}\right)\left(u_{5}\right)$

(4, [0.83,1.36])

$C\left(k_{1},k_{6}\right)\left(u_{6}\right)$

(6, [0.73,1.46])

$C\left(k_{3},k_{6}\right)\left(u_{6}\right)$

(6, [0.63,1.63])

*Now we will evaluate the Score* $S\left(k_{s^{\prime }},l_{t^{\prime }}\right)\left(u_{i}\right);\;\forall \;u_{i}\in U,\;\left(k_{s^{\prime }},l_{t^{\prime }}\right)\in \left(K\times L\right),\;\;s^{\prime }=1,3\;and\;t^{\prime }=4,5,6$ *by using step 4 of Algorithm 1.*


$S\left(k_{s^{\prime }},k_{t^{\prime }}\right)\left(u_{i}\right)$


***The Score***


$S\left(k_{s^{\prime }},k_{t^{\prime }}\right)\left(u_{i}\right)$


***The Score***


$S\left(k_{1},k_{4}\right)\left(u_{1}\right)$

−52.14

$S\left(k_{3},k_{4}\right)\left(u_{1}\right)$

−10.34

$S\left(k_{1},k_{4}\right)\left(u_{2}\right)$

31.02

$S\left(k_{3},k_{4}\right)\left(u_{2}\right)$

42.46

$S\left(k_{1},k_{4}\right)\left(u_{3}\right)$

−102.18

$S\left(k_{3},k_{4}\right)\left(u_{3}\right)$

−79.3

$S\left(k_{1},k_{4}\right)\left(u_{4}\right)$

4.41

$S\left(k_{3},k_{4}\right)\left(u_{4}\right)$

−41.8

$S\left(k_{1},k_{4}\right)\left(u_{5}\right)$

158.76

$S\left(k_{3},k_{4}\right)\left(u_{5}\right)$

84.6

$S\left(k_{1},k_{4}\right)\left(u_{6}\right)$

5.94

$S\left(k_{3},k_{4}\right)\left(u_{6}\right)$

25.27

$S\left(k_{1},k_{5}\right)\left(u_{1}\right)$

−48.64

$S\left(k_{3},k_{5}\right)\left(u_{1}\right)$

−21.24

$S\left(k_{1},k_{5}\right)\left(u_{2}\right)$

79.12

$S\left(k_{3},k_{5}\right)\left(u_{2}\right)$

92.92

$S\left(k_{1},k_{5}\right)\left(u_{3}\right)$

−150.48

$S\left(k_{3},k_{5}\right)\left(u_{3}\right)$

−107.28

$S\left(k_{1},k_{5}\right)\left(u_{4}\right)$

37.82

$S\left(k_{3},k_{5}\right)\left(u_{4}\right)$

−47.74

$S\left(k_{1},k_{5}\right)\left(u_{5}\right)$

16.34

$S\left(k_{3},k_{5}\right)\left(u_{5}\right)$

−11.02

$S\left(k_{1},k_{5}\right)\left(u_{6}\right)$

28.06

$S\left(k_{3},k_{5}\right)\left(u_{6}\right)$

51.52

$S\left(k_{1},k_{6}\right)\left(u_{1}\right)$

−55.89

$S\left(k_{3},k_{6}\right)\left(u_{1}\right)$

−27.6

$S\left(k_{1},k_{6}\right)\left(u_{2}\right)$

70.08

$S\left(k_{3},k_{6}\right)\left(u_{2}\right)$

59.2

$S\left(k_{1},k_{6}\right)\left(u_{3}\right)$

−22.77

$S\left(k_{3},k_{6}\right)\left(u_{3}\right)$

1.25

$S\left(k_{1},k_{6}\right)\left(u_{4}\right)$

−106.92

$S\left(k_{3},k_{6}\right)\left(u_{4}\right)$

−158.04

$S\left(k_{1},k_{6}\right)\left(u_{5}\right)$

93.09

$S\left(k_{3},k_{6}\right)\left(u_{5}\right)$

46.69

$S\left(k_{1},k_{6}\right)\left(u_{6}\right)$

35.64

$S\left(k_{3},k_{6}\right)\left(u_{6}\right)$

73.08

*By using step 5 of Algorithm 1 the weighted values for each* $S(u_{l})$ *are:*

$$S\left(u_{1}\right)=-215.85,\;S\left(u_{2}\right)=\;374.8,\;S\left(u_{3}\right)=-460.76,$$

$$S\left(u_{4}\right)=-312.27,S\left(u_{5}\right)=\;\;388.46,\;\;S\left(u_{6}\right)=219.51.$$

*Here the optimal decision by using step 6 of Algorithm 1 is:*

$$X=arg\;max\;\{S\left(u_{1}\right),S\left(u_{2}\right),S\left(u_{3}\right),S\left(u_{4}\right),S\left(u_{5}\right),S\left(u_{6}\right)\}.$$

*Thus,*$u_{5}$ is the best choice. Hence Mr. H will buy the $u_{5}$ *smartphone.*

**Figure 3:**
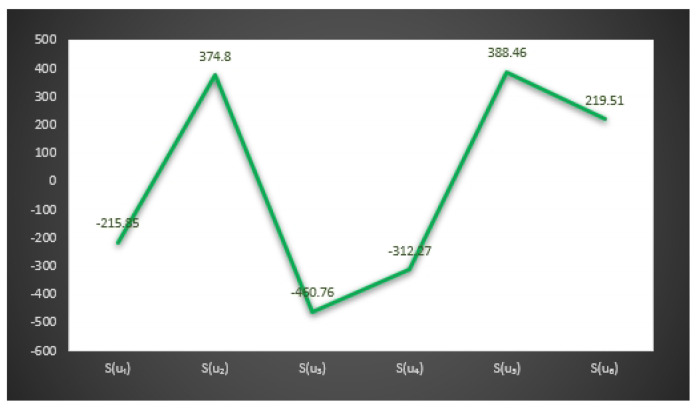
Graphical behavior of score values of Example 11 by means of Algorithm 1.

**Figure 4:**
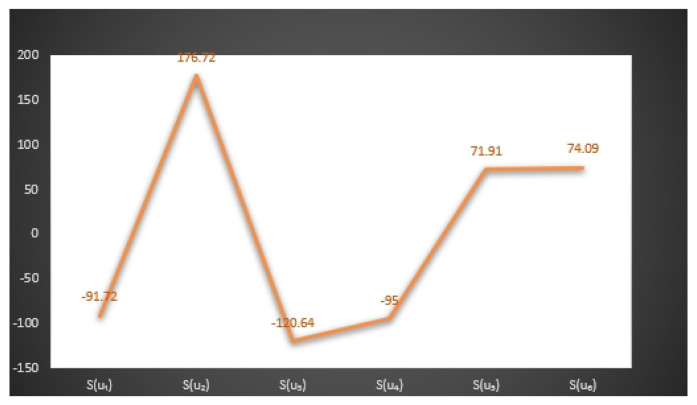
Graphical behavior of score values of Example 12 by means of Algorithm 2.

In Figure 3 and Figure 4, we give the graphical behavior of score values of Examples 11 and 12 by means of Algorithms 1 and 2 respectively.

**Example** **12.** *Consider the Example 11 and assume that Mr.*H*wants to buy one such smartphone depending on one of two parameters only*.

*Firstly, we will evaluate the soft min-OR operation* $\left(Q_{q},K\times L,4\right)=\left(B_{b},K,4\right)\hat{\vee }\left(Y_{y},L,7\right)$ *by using step 2 of Algorithm 2:*

$$\begin{array}{c} \begin{array}{cc} (Q_{q},K\times L,4)=\{ & (\left(k_{1},k_{4}\right),(\frac{\left(u_{1},1\right)}{\left[0.43,0.80],[0.30,0.60\right]},\frac{\left(u_{2},1\right)}{\left[0.60,0.90],[0.36,0.73\right]},\frac{\left(u_{3},1\right)}{\left[0.53,0.66],[0.16,0.53\right]},\\ & \;\;\;\;\;\;\;\;\;\;\;\;\;\;\;\;\;\;\;\;\;\frac{\left(u_{4},1\right)}{\left[0.53,0.86],[0.43,0.56\right]},\frac{\left(u_{5},0\right)}{\left[0.46,0.80],[0.76,0.96\right]},\frac{\left(u_{6},0\right)}{\left[0.43,0.86],[0.36,0.73\right]})),\\ & (\left(k_{1},k_{5}\right),(\frac{\left(u_{1},0\right)}{\left[0.10,0.66],[0.36,0.83\right]},\frac{\left(u_{2},2\right)}{\left[0.76,0.90],[0.50,0.80\right]},\frac{\left(u_{3},1\right)}{\left[0.40,0.73],[0.16,0.63\right]},\\ & \;\;\;\;\;\;\;\;\;\;\;\;\;\;\;\;\;\;\;\;\;\frac{\left(u_{4},2\right)}{\left[0.66,0.93],[0.46,0.56\right]},\frac{\left(u_{5},0\right)}{\left[0.63,0.86],[0.56,0.73\right]},\frac{\left(u_{6},3\right)}{\left[0.60,0.76],[0.53,0.73\right]})),\\ & (\left(k_{1},k_{6}\right),(\frac{\left(u_{1},1\right)}{\left[0.16,0.73],[0.36,0.60\right]},\frac{\left(u_{2},3\right)}{\left[0.80,0.90],[0.26,0.63\right]},\frac{\left(u_{3},0\right)}{\left[0.46,0.66],[0.33,0.76\right]},\\ & \;\;\;\;\;\;\;\;\;\;\;\;\;\;\;\;\;\;\;\;\;\frac{\left(u_{4},2\right)}{\left[0.50,0.66],[0.40,0.50\right]},\frac{\left(u_{5},0\right)}{\left[0.56,0.83],[0.63,0.80\right]},\frac{\left(u_{6},3\right)}{\left[0.46,0.86],[0.40,0.66\right]})),\\ & (\left(k_{3},k_{4}\right),(\frac{\left(u_{1},0\right)}{\left[0.56,0.80],[0.16,0.66\right]},\frac{\left(u_{2},1\right)}{\left[0.33,0.86],[0.60,0.80\right]},\frac{\left(u_{3},2\right)}{\left[0.56,0.66],[0.30,0.40\right]},\\ & \;\;\;\;\;\;\;\;\;\;\;\;\;\;\;\;\;\;\;\;\;\frac{\left(u_{4},1\right)}{\left[0.20,0.70],[0.43,0.63\right]},\frac{\left(u_{5},1\right)}{\left[0.36,0.66],[0.66,0.93\right]},\frac{\left(u_{6},0\right)}{\left[0.63,0.96],[0.23,0.76\right]})),\\ & (\left(k_{3},k_{5}\right),(\frac{\left(u_{1},0\right)}{\left[0.36,0.66],[0.26,0.86\right]},\frac{\left(u_{2},2\right)}{\left[0.60,0.86],[0.66,0.86\right]},\frac{\left(u_{3},2\right)}{\left[0.43,0.73],[0.30,0.50\right]},\\ & \;\;\;\;\;\;\;\;\;\;\;\;\;\;\;\;\;\;\;\;\;\frac{\left(u_{4},2\right)}{\left[0.46,0.76],[0.46,0.63\right]},\frac{\left(u_{5},1\right)}{\left[0.56,0.76],[0.36,0.66\right]},\frac{\left(u_{6},1\right)}{\left[0.80,0.90],[0.43,0.76\right]})),\\ & (\left(k_{3},k_{6}\right),(\frac{\left(u_{1},0\right)}{\left[0.40,0.73],[0.26,0.66\right]},\frac{\left(u_{2},3\right)}{\left[0.66,0.86],[0.53,0.70\right]},\frac{\left(u_{3},0\right)}{\left[0.50,0.66],[0.40,0.70\right]},\\ & \;\;\;\;\;\;\;\;\;\;\;\;\;\;\;\;\;\;\;\;\;\frac{\left(u_{4},2\right)}{\left[0.16,0.36],[0.40,0.56\right]},\frac{\left(u_{5},1\right)}{\left[0.50,0.70],[0.46,0.73\right]},\frac{\left(u_{6},1\right)}{\left[0.66,0.96],[0.30,0.70\right]}))\}. \end{array} \end{array}$$

*Then we will evaluate the choice value* $C\left(k_{s^{\prime }},l_{t^{\prime }}\right)\left(u_{i}\right);\;\forall \;u_{i}\in U,\;\left(k_{s^{\prime }},l_{t^{\prime }}\right)\in \left(K\times L\right),\;\;s^{\prime }=1,3\;and\;t^{\prime }=4,5,6$ *by using step 3 of Algorithm 2:*


$C\left(k_{s^{\prime }},k_{t^{\prime }}\right)\left(u_{i}\right)$


***(Grading, Interval Value)***


$C\left(k_{s^{\prime }},k_{t^{\prime }}\right)\left(u_{i}\right)$


***(Grading, Interval Value)***


$C\left(k_{1},k_{4}\right)\left(u_{1}\right)$

(1, [0.73,1.40])

$C\left(k_{3},k_{4}\right)\left(u_{1}\right)$

(0, [0.73,1.46])

$C\left(k_{1},k_{4}\right)\left(u_{2}\right)$

(1, [0.96,1.63])

$C\left(k_{3},k_{4}\right)\left(u_{2}\right)$

(1, [0.93,1.66])

$C\left(k_{1},k_{4}\right)\left(u_{3}\right)$

(1, [0.70,1.20])

$C\left(k_{3},k_{4}\right)\left(u_{3}\right)$

(2, [0.86,1.06])

$C\left(k_{1},k_{4}\right)\left(u_{4}\right)$

(1, [0.96,1.43])

$C\left(k_{3},k_{4}\right)\left(u_{4}\right)$

(1, [0.63,1.33])

$C\left(k_{1},k_{4}\right)\left(u_{5}\right)$

(0, [1.23,1.76])

$C\left(k_{3},k_{4}\right)\left(u_{5}\right)$

(1, [1.03,1.60])

$C\left(k_{1},k_{4}\right)\left(u_{6}\right)$

(0, [0.80,1.60])

$C\left(k_{3},k_{4}\right)\left(u_{6}\right)$

(0, [0.86,1.73])

$C\left(k_{1},k_{5}\right)\left(u_{1}\right)$

(0, [0.46,1.50])

$C\left(k_{3},k_{5}\right)\left(u_{1}\right)$

(0, [0.63,1.53])

$C\left(k_{1},k_{5}\right)\left(u_{2}\right)$

(2, [1.26,1.70])

$C\left(k_{3},k_{5}\right)\left(u_{2}\right)$

(2, [1.26,1.73])

$C\left(k_{1},k_{5}\right)\left(u_{3}\right)$

(1, [0.56,1.36])

$C\left(k_{3},k_{5}\right)\left(u_{3}\right)$

(2, [0.73,1.23])

$C\left(k_{1},k_{5}\right)\left(u_{4}\right)$

(2, [1.13,1.50])

$C\left(k_{3},k_{5}\right)\left(u_{4}\right)$

(2, [0.93,1.40])

$C\left(k_{1},k_{5}\right)\left(u_{5}\right)$

(0, [1.20,1.60])

$C\left(k_{3},k_{5}\right)\left(u_{5}\right)$

(1, [0.93,1.43])

$C\left(k_{1},k_{5}\right)\left(u_{6}\right)$

(3, [1.13,1.50])

$C\left(k_{3},k_{5}\right)\left(u_{6}\right)$

(1, [1.23,1.66])

$C\left(k_{1},k_{6}\right)\left(u_{1}\right)$

(1, [0.53,1.33])

$C\left(k_{3},k_{6}\right)\left(u_{1}\right)$

(0, [0.66,1.40])

$C\left(k_{1},k_{6}\right)\left(u_{2}\right)$

(3, [1.06,1.53])

$C\left(k_{3},k_{6}\right)\left(u_{2}\right)$

(3, [1.20,1.56])

$C\left(k_{1},k_{6}\right)\left(u_{3}\right)$

(0, [0.80,1.43])

$C\left(k_{3},k_{6}\right)\left(u_{3}\right)$

(0, [0.90,1.36])

$C\left(k_{1},k_{6}\right)\left(u_{4}\right)$

(2, [0.90,1.16])

$C\left(k_{3},k_{6}\right)\left(u_{4}\right)$

(2, [0.56,0.93])

$C\left(k_{1},k_{6}\right)\left(u_{5}\right)$

(0, [1.20,1.63])

$C\left(k_{3},k_{6}\right)\left(u_{5}\right)$

(1, [0.96,1.43])

$C\left(k_{1},k_{6}\right)\left(u_{6}\right)$

(3, [0.86,1.53])

$C\left(k_{3},k_{6}\right)\left(u_{6}\right)$

(1, [0.96,1.66])

*Now we will evaluate the Score* $S\left(k_{s^{\prime }},l_{t^{\prime }}\right)\left(u_{i}\right);\;\forall \;u_{i}\in U,\;\left(k_{s^{\prime }},l_{t^{\prime }}\right)\in \left(K\times L\right),\;\;s^{\prime }=1,3\;and\;t^{\prime }=4,5,6$ *by using step 4 of Algorithm 2.*


$S\left(k_{s^{\prime }},k_{t^{\prime }}\right)\left(u_{i}\right)$


***The Score***


$S\left(k_{s^{\prime }},k_{t^{\prime }}\right)\left(u_{i}\right)$


***The Score***


$S\left(k_{1},k_{4}\right)\left(u_{1}\right)$

−9.72

$S\left(k_{3},k_{4}\right)\left(u_{1}\right)$

−3.7

$S\left(k_{1},k_{4}\right)\left(u_{2}\right)$

6.84

$S\left(k_{3},k_{4}\right)\left(u_{2}\right)$

11.62

$S\left(k_{1},k_{4}\right)\left(u_{3}\right)$

−18

$S\left(k_{3},k_{4}\right)\left(u_{3}\right)$

−28.32

$S\left(k_{1},k_{4}\right)\left(u_{4}\right)$

−0.36

$S\left(k_{3},k_{4}\right)\left(u_{4}\right)$

−14.84

$S\left(k_{1},k_{4}\right)\left(u_{5}\right)$

14.16

$S\left(k_{3},k_{4}\right)\left(u_{5}\right)$

13.3

$S\left(k_{1},k_{4}\right)\left(u_{6}\right)$

0

$S\left(k_{3},k_{4}\right)\left(u_{6}\right)$

8.3

$S\left(k_{1},k_{5}\right)\left(u_{1}\right)$

−25.12

$S\left(k_{3},k_{5}\right)\left(u_{1}\right)$

−13.84

$S\left(k_{1},k_{5}\right)\left(u_{2}\right)$

37.18

$S\left(k_{3},k_{5}\right)\left(u_{2}\right)$

39

$S\left(k_{1},k_{5}\right)\left(u_{3}\right)$

−33.8

$S\left(k_{3},k_{5}\right)\left(u_{3}\right)$

−35.16

$S\left(k_{1},k_{5}\right)\left(u_{4}\right)$

11.44

$S\left(k_{3},k_{5}\right)\left(u_{4}\right)$

−8.52

$S\left(k_{1},k_{5}\right)\left(u_{5}\right)$

15.2

$S\left(k_{3},k_{5}\right)\left(u_{5}\right)$

−4.77

$S\left(k_{1},k_{5}\right)\left(u_{6}\right)$

15.84

$S\left(k_{3},k_{5}\right)\left(u_{6}\right)$

23.85

$S\left(k_{1},k_{6}\right)\left(u_{1}\right)$

−30.8

$S\left(k_{3},k_{6}\right)\left(u_{1}\right)$

−8.54

$S\left(k_{1},k_{6}\right)\left(u_{2}\right)$

28.44

$S\left(k_{3},k_{6}\right)\left(u_{2}\right)$

53.64

$S\left(k_{1},k_{6}\right)\left(u_{3}\right)$

−5.22

$S\left(k_{3},k_{6}\right)\left(u_{3}\right)$

−0.14

$S\left(k_{1},k_{6}\right)\left(u_{4}\right)$

−22.4

$S\left(k_{3},k_{6}\right)\left(u_{4}\right)$

−60.32

$S\left(k_{1},k_{6}\right)\left(u_{5}\right)$

27.18

$S\left(k_{3},k_{6}\right)\left(u_{5}\right)$

6.84

$S\left(k_{1},k_{6}\right)\left(u_{6}\right)$

6.84

$S\left(k_{3},k_{6}\right)\left(u_{6}\right)$

19.26

*By using step 5 of Algorithm 2 the weighted values for each* $S(u_{l})$ *is:*

$$S\left(u_{1}\right)=-91.72,\;S\left(u_{2}\right)=1776.72,\;S\left(u_{3}\right)=-120.64,$$

$$S\left(u_{4}\right)=-95,\;S\left(u_{5}\right)=\;71.91,\;S\left(u_{6}\right)=74.09.$$

*Here the optimal decision by using step 6 of Algorithm 2 is:*

$$X=arg\;max\;\{S\left(u_{1}\right),S\left(u_{2}\right),S\left(u_{3}\right),S\left(u_{4}\right),S\left(u_{5}\right),S\left(u_{6}\right)\}.$$

*Thus,* $u_{2}$ *is the best choice. Hence Mr.* H *will buy the* $u_{2}$ *smartphone.*

**Figure 5:**
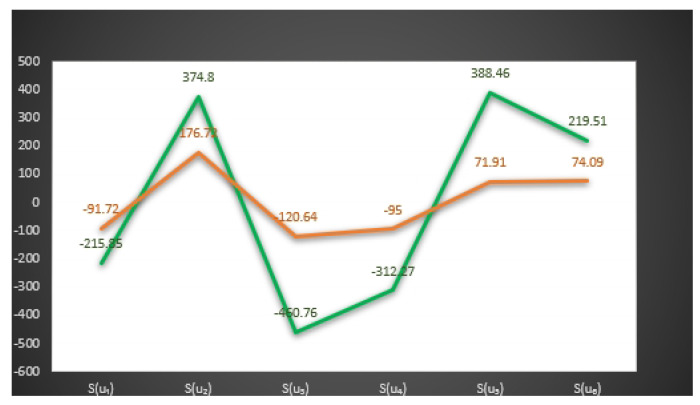
Comparison of score values of Examples 11 and 12 by means of Algorithms 1 and 2.

In Figure 5, we observe that the following relations between the score values of Examples 11 and 12 by means of Algorithms 1 and 2, respectively.

$$S\left(u_{3}\right)⪯S\left(u_{4}\right)⪯S\left(u_{1}\right)⪯S\left(u_{6}\right)⪯S\left(u_{2}\right)⪯S\left(u_{5}\right),$$

$$S\left(u_{3}\right)⪯S\left(u_{4}\right)⪯S\left(u_{1}\right)⪯S\left(u_{5}\right)⪯S\left(u_{6}\right)⪯S\left(u_{2}\right).$$

**Example** **13.** *Mr.*X*wants to select a personal secretary with remarkable qualities, and there are three persons under consideration. Let*$U=\{u_{1},u_{2},u_{3},\;u_{4},\;u_{5},\;u_{6}\}$*be the set of persons. Let*$E=\{k_{1}=communication\;and\;planning\;skills,\;k_{2}=multitasking\;skills,\;k_{3}=time\;management,\;k_{4}=judgement\;ability,\;k_{5}=hardworking,\;k_{6}=confident,\;k_{7}=technology\;skills\}$*be the set of parameters (remarkable qualities) for the selection of personal secretary and*K,L⊆E*such that*$K=\left\{k_{1},k_{3},k_{5}\right\}\;and\;L=\left\{k_{4},k_{5},k_{7}\right\}$. *Assume that Mr.*X*wants to select one such person depending on two parameters only. Suppose there are two observations*$\left(B_{b},K,5\right)$*and*$\left(Y_{y},L,8\right)$*by two experts as follows:*

$$\begin{array}{c} \begin{array}{cc} (B_{b},K,5)=\{ & (k_{1},(\frac{\left(u_{1},1\right)}{\left[0.5,0.8],[0.3,0.5\right]},\frac{\left(u_{2},3\right)}{\left[0.2,0.9],[0.3,0.7\right]},\frac{\left(u_{3},0\right)}{\left[0.1,0.3],[0.1,0.4\right]},\\ & \;\;\;\;\;\;\;\;\;\;\;\;\;\frac{\left(u_{4},2\right)}{\left[0.1,0.3],[0.5,0.6\right]},\frac{\left(u_{5},4\right)}{\left[0.4,0.7],[0.3,0.9\right]},\frac{\left(u_{6},3\right)}{\left[0.6,0.7],[0.3,0.6\right]})),\\ & (k_{3},(\frac{\left(u_{1},0\right)}{\left[0.4,0.9],[0.3,0.6\right]},\frac{\left(u_{2},4\right)}{\left[0.4,0.7],[0.5,0.9\right]},\frac{\left(u_{3},1\right)}{\left[0.8,0.9],[0.4,0.9\right]},\\ & \;\;\;\;\;\;\;\;\;\;\;\;\;\frac{\left(u_{4},3\right)}{\left[0.3,0.5],[0.4,0.6\right]},\frac{\left(u_{5},2\right)}{\left[0.8,0.9],[0.3,0.7\right]},\frac{\left(u_{6},4\right)}{\left[0.2,0.4],[0.3,0.5\right]})),\\ & (k_{5},(\frac{\left(u_{1},4\right)}{\left[0.3,0.7],[0.2,0.5\right]},\frac{\left(u_{2},3\right)}{\left[0.3,0.8],[0.2,0.9\right]},\frac{\left(u_{3},2\right)}{\left[0.7,0.9],[0.5,0.8\right]},\\ & \;\;\;\;\;\;\;\;\;\;\;\;\;\frac{\left(u_{4},3\right)}{\left[0.7,0.8],[0.5,0.6\right]},\frac{\left(u_{5},0\right)}{\left[0.1,0.7],[0.3,0.4\right]},\frac{\left(u_{6},1\right)}{\left[0.7,1.0],[0.5,0.8\right]}))\}. \end{array} \end{array}$$

$$\begin{array}{c} \begin{array}{cc} (Y_{y},L,8)=\{ & (k_{4},(\frac{\left(u_{1},5\right)}{\left[0.1,0.7],[0.3,0.8\right]},\frac{\left(u_{2},6\right)}{\left[0.5,0.7],[0.5,0.7\right]},\frac{\left(u_{3},3\right)}{\left[0.3,0.4],[0.5,0.6\right]},\\ & \;\;\;\;\;\;\;\;\;\;\;\;\;\frac{\left(u_{4},7\right)}{\left[0.1,0.6],[0.2,0.4\right]},\frac{\left(u_{5},4\right)}{\left[0.5,0.6],[0.4,1.0\right]},\frac{\left(u_{6},2\right)}{\left[0.3,0.6],[0.2,0.8\right]})),\\ & (k_{5},(\frac{\left(u_{1},2\right)}{\left[0.3,0.5],[0.6,0.7\right]},\frac{\left(u_{2},1\right)}{\left[0.3,0.9],[0.3,0.6\right]},\frac{\left(u_{3},0\right)}{\left[0.2,0.3],[0.3,0.5\right]},\\ & \;\;\;\;\;\;\;\;\;\;\;\;\;\frac{\left(u_{4},5\right)}{\left[0.5,0.8],[0.6,0.7\right]},\frac{\left(u_{5},4\right)}{\left[0.6,0.9],[0.7,0.8\right]},\frac{\left(u_{6},3\right)}{\left[0.3,0.5],[0.2,0.6\right]})),\\ & (k_{7},(\frac{\left(u_{1},7\right)}{\left[0.8,0.9],[0.4,0.5\right]},\frac{\left(u_{2},4\right)}{\left[0.6,0.8],[0.4,0.7\right]},\frac{\left(u_{3},5\right)}{\left[0.1,0.9],[0.1,0.8\right]},\\ & \;\;\;\;\;\;\;\;\;\;\;\;\;\frac{\left(u_{4},3\right)}{\left[0.1,0.5],[0.2,0.7\right]},\frac{\left(u_{5},2\right)}{\left[0.2,0.3],[0.4,0.5\right]},\frac{\left(u_{6},6\right)}{\left[0.4,0.7],[0.5,0.6\right]}))\}. \end{array} \end{array}$$

*Firstly, we will evaluate the soft max-AND operation* $\left(G_{g},K\times L,8\right)=\left(B_{b},K,5\right)\hat{\wedge }\left(Y_{y},L,8\right)$ *by using step 2 of Algorithm 1:*

$$\begin{array}{c} \begin{array}{cc} (G_{g},K\times L,8)=\{ & (\left(k_{1},k_{4}\right),(\frac{\left(u_{1},5\right)}{\left[0.36,0.76],[0.30,0.70\right]},\frac{\left(u_{2},6\right)}{\left[0.40,0.83],[0.43,0.70\right]},\frac{\left(u_{3},3\right)}{\left[0.23,0.36],[0.36,0.53\right]},\\ & \;\;\;\;\;\;\;\;\;\;\;\;\;\;\;\;\;\;\;\;\;\frac{\left(u_{4},7\right)}{\left[0.10,0.50],[0.40,0.53\right]},\frac{\left(u_{5},4\right)}{\left[0.46,0.66],[0.36,0.96\right]},\frac{\left(u_{6},3\right)}{\left[0.50,0.66],[0.26,0.73\right]})),\\ & (\left(k_{1},k_{5}\right),(\frac{\left(u_{1},2\right)}{\left[0.43,0.70],[0.50,0.63\right]},\frac{\left(u_{2},3\right)}{\left[0.26,0.90],[0.30,0.66\right]},\frac{\left(u_{3},0\right)}{\left[0.16,0.30],[0.23,0.46\right]},\\ & \;\;\;\;\;\;\;\;\;\;\;\;\;\;\;\;\;\;\;\;\;\frac{\left(u_{4},5\right)}{\left[0.36,0.63],[0.56,0.66\right]},\frac{\left(u_{5},4\right)}{\left[0.53,0.83],[0.56,0.86\right]},\frac{\left(u_{6},3\right)}{\left[0.50,0.63],[0.26,0.60\right]})),\\ & (\left(k_{1},k_{7}\right),(\frac{\left(u_{1},7\right)}{\left[0.70,0.86],[0.36,0.50\right]},\frac{\left(u_{2},4\right)}{\left[0.46,0.86],[0.36,0.70\right]},\frac{\left(u_{3},5\right)}{\left[0.10,0.70],[0.10,0.66\right]},\\ & \;\;\;\;\;\;\;\;\;\;\;\;\;\;\;\;\;\;\;\;\;\frac{\left(u_{4},3\right)}{\left[0.10,0.43],[0.40,0.66\right]},\frac{\left(u_{5},4\right)}{\left[0.33,0.56],[0.36,0.76\right]},\frac{\left(u_{6},6\right)}{\left[0.53,0.70],[0.43,0.60\right]})),\\ & (\left(k_{3},k_{4}\right),(\frac{\left(u_{1},5\right)}{\left[0.30,0.83],[0.30,0.73\right]},\frac{\left(u_{2},6\right)}{\left[0.46,0.70],[0.50,0.83\right]},\frac{\left(u_{3},3\right)}{\left[0.63,0.73],[0.46,0.80\right]},\\ & \;\;\;\;\;\;\;\;\;\;\;\;\;\;\;\;\;\;\;\;\;\frac{\left(u_{4},7\right)}{\left[0.23,0.56],[0.33,0.53\right]},\frac{\left(u_{5},4\right)}{\left[0.70,0.80],[0.36,0.90\right]},\frac{\left(u_{6},4\right)}{\left[0.26,0.53],[0.26,0.70\right]})),\\ & (\left(k_{3},k_{5}\right),(\frac{\left(u_{1},2\right)}{\left[0.36,0.76],[0.50,0.66\right]},\frac{\left(u_{2},4\right)}{\left[0.36,0.83],[0.43,0.80\right]},\frac{\left(u_{3},1\right)}{\left[0.60,0.70],[0.36,0.76\right]},\\ & \;\;\;\;\;\;\;\;\;\;\;\;\;\;\;\;\;\;\;\;\;\frac{\left(u_{4},5\right)}{\left[0.43,0.70],[0.53,0.66\right]},\frac{\left(u_{5},4\right)}{\left[0.73,0.90],[0.56,0.76\right]},\frac{\left(u_{6},4\right)}{\left[0.26,0.46],[0.26,0.56\right]})),\\ & (\left(k_{3},k_{7}\right),(\frac{\left(u_{1},7\right)}{\left[0.66,0.90],[0.36,0.56\right]},\frac{\left(u_{2},4\right)}{\left[0.53,0.76],[0.46,0.83\right]},\frac{\left(u_{3},5\right)}{\left[0.56,0.90],[0.30,0.86\right]},\\ & \;\;\;\;\;\;\;\;\;\;\;\;\;\;\;\;\;\;\;\;\;\frac{\left(u_{4},3\right)}{\left[0.23,0.50],[0.33,0.66\right]},\frac{\left(u_{5},2\right)}{\left[0.60,0.70],[0.36,0.63\right]},\frac{\left(u_{6},6\right)}{\left[0.33,0.60],[0.43,0.56\right]})),\\ & (\left(k_{5},k_{4}\right),(\frac{\left(u_{1},5\right)}{\left[0.23,0.70],[0.26,0.70\right]},\frac{\left(u_{2},6\right)}{\left[0.43,0.76],[0.40,0.83\right]},\frac{\left(u_{3},3\right)}{\left[0.56,0.73],[0.50,0.73\right]},\\ & \;\;\;\;\;\;\;\;\;\;\;\;\;\;\;\;\;\;\;\;\;\frac{\left(u_{4},7\right)}{\left[0.50,0.73],[0.40,0.53\right]},\frac{\left(u_{5},4\right)}{\left[0.36,0.66],[0.36,0.80\right]},\frac{\left(u_{6},2\right)}{\left[0.56,0.86],[0.40,0.80\right]})),\\ & (\left(k_{5},k_{5}\right),(\frac{\left(u_{1},4\right)}{\left[0.30,0.63],[0.46,0.63\right]},\frac{\left(u_{2},3\right)}{\left[0.30,0.86],[0.26,0.80\right]},\frac{\left(u_{3},2\right)}{\left[0.53,0.70],[0.43,0.70\right]},\\ & \;\;\;\;\;\;\;\;\;\;\;\;\;\;\;\;\;\;\;\;\;\frac{\left(u_{4},5\right)}{\left[0.63,0.80],[0.56,0.66\right]},\frac{\left(u_{5},4\right)}{\left[0.43,0.83],[0.56,0.66\right]},\frac{\left(u_{6},3\right)}{\left[0.56,0.83],[0.40,0.73\right]})),\\ & (\left(k_{5},k_{7}\right),(\frac{\left(u_{1},7\right)}{\left[0.63,0.83],[0.33,0.50\right]},\frac{\left(u_{2},4\right)}{\left[0.50,0.80],[0.33,0.83\right]},\frac{\left(u_{3},5\right)}{\left[0.50,0.90],[0.36,0.80\right]},\\ & \;\;\;\;\;\;\;\;\;\;\;\;\;\;\;\;\;\;\;\;\;\frac{\left(u_{4},3\right)}{\left[0.50,0.70],[0.40,0.66\right]},\frac{\left(u_{5},2\right)}{\left[0.16,0.56],[0.36,0.46\right]},\frac{\left(u_{6},6\right)}{\left[0.60,0.90],[0.50,0.73\right]}))\}. \end{array} \end{array}$$

*Then we will evaluate the choice value* $C\left(k_{s^{\prime }},l_{t^{\prime }}\right)\left(u_{i}\right);\;\forall \;u_{i}\in U,\;\left(k_{s^{\prime }},l_{t^{\prime }}\right)\in \left(K\times L\right),\;\;s^{\prime }=1,3,5\;and\;t^{\prime }=4,5,7$ *by using step 3 of Algorithm 1:*


$C\left(k_{s^{\prime }},k_{t^{\prime }}\right)\left(u_{i}\right)$


***(Grading, Interval Value)***


$C\left(k_{s^{\prime }},k_{t^{\prime }}\right)\left(u_{i}\right)$


***(Grading, Interval Value)***


$C\left(k_{1},k_{4}\right)\left(u_{1}\right)$

(5, [0.53,1.33])

$C\left(k_{3},k_{5}\right)\left(u_{4}\right)$

(5, [0.83,1.23])

$C\left(k_{1},k_{4}\right)\left(u_{2}\right)$

(6, [0.66,1.46])

$C\left(k_{3},k_{5}\right)\left(u_{5}\right)$

(4, [1.10,1.63])

$C\left(k_{1},k_{4}\right)\left(u_{3}\right)$

(3, [0.40,0.80])

$C\left(k_{3},k_{5}\right)\left(u_{6}\right)$

(4, [0.46,0.96])

$C\left(k_{1},k_{4}\right)\left(u_{4}\right)$

(7, [0.40,0.86])

$C\left(k_{3},k_{7}\right)\left(u_{1}\right)$

(7, [0.86,1.43])

$C\left(k_{1},k_{4}\right)\left(u_{5}\right)$

(4, [0.76,1.56])

$C\left(k_{3},k_{7}\right)\left(u_{2}\right)$

(4, [0.90,1.50])

$C\left(k_{1},k_{4}\right)\left(u_{6}\right)$

(3, [0.63,1.30])

$C\left(k_{3},k_{7}\right)\left(u_{3}\right)$

(5, [0.53,1.73])

$C\left(k_{1},k_{5}\right)\left(u_{1}\right)$

(2, [0.76,1.16])

$C\left(k_{3},k_{7}\right)\left(u_{4}\right)$

(3, [0.43,1.13])

$C\left(k_{1},k_{5}\right)\left(u_{2}\right)$

(3, [0.53,1.53])

$C\left(k_{3},k_{7}\right)\left(u_{5}\right)$

(2, [0.73,1.06])

$C\left(k_{1},k_{5}\right)\left(u_{3}\right)$

(0, [0.30,1.73])

$C\left(k_{3},k_{7}\right)\left(u_{6}\right)$

(6, [0.63,1.03])

$C\left(k_{1},k_{5}\right)\left(u_{4}\right)$

(5, [0.76,1.10])

$C\left(k_{5},k_{4}\right)\left(u_{1}\right)$

(5, [0.40,1.30])

$C\left(k_{1},k_{5}\right)\left(u_{5}\right)$

(4, [0.90,1.60])

$C\left(k_{5},k_{4}\right)\left(u_{2}\right)$

(6, [0.67,1.50])

$C\left(k_{1},k_{5}\right)\left(u_{6}\right)$

(3, [0.63,1.16])

$C\left(k_{5},k_{4}\right)\left(u_{3}\right)$

(3, [0.93,1.23])

$C\left(k_{1},k_{7}\right)\left(u_{1}\right)$

(7, [0.93,1.33])

$C\left(k_{5},k_{4}\right)\left(u_{4}\right)$

(7, [0.60,1.13])

$C\left(k_{1},k_{7}\right)\left(u_{2}\right)$

(4, [0.66,1.53])

$C\left(k_{5},k_{4}\right)\left(u_{5}\right)$

(4, [0.56,1.23])

$C\left(k_{1},k_{7}\right)\left(u_{3}\right)$

(5, [0.20,1.03])

$C\left(k_{5},k_{4}\right)\left(u_{6}\right)$

(2, [0.73,1.53])

$C\left(k_{1},k_{7}\right)\left(u_{4}\right)$

(3, [0.40,1.00])

$C\left(k_{5},k_{5}\right)\left(u_{1}\right)$

(4, [0.63,1.13])

$C\left(k_{1},k_{7}\right)\left(u_{5}\right)$

(4, [0.60,1.06])

$C\left(k_{5},k_{5}\right)\left(u_{2}\right)$

(3, [0.53,1.53])

$C\left(k_{1},k_{7}\right)\left(u_{6}\right)$

(6, [0.83,1.30])

$C\left(k_{5},k_{5}\right)\left(u_{3}\right)$

(2, [0.73,1.10])

$C\left(k_{3},k_{4}\right)\left(u_{1}\right)$

(5, [0.50,1.43])

$C\left(k_{5},k_{5}\right)\left(u_{4}\right)$

(5, [1.10,1.43])

$C\left(k_{3},k_{4}\right)\left(u_{2}\right)$

(6, [0.93,1.46])

$C\left(k_{5},k_{5}\right)\left(u_{5}\right)$

(4, [0.70,1.30])

$C\left(k_{3},k_{4}\right)\left(u_{3}\right)$

(3, [1.90,1.26])

$C\left(k_{5},k_{5}\right)\left(u_{6}\right)$

(3, [0.73,1.33])

$C\left(k_{3},k_{4}\right)\left(u_{4}\right)$

(7, [0.43,1.00])

$C\left(k_{5},k_{7}\right)\left(u_{1}\right)$

(7, [0.73,1.26])

$C\left(k_{3},k_{4}\right)\left(u_{5}\right)$

(4, [0.93,1.50])

$C\left(k_{5},k_{7}\right)\left(u_{2}\right)$

(4, [0.66,1.56])

$C\left(k_{3},k_{4}\right)\left(u_{6}\right)$

(4, [0.46,1.06])

$C\left(k_{5},k_{7}\right)\left(u_{3}\right)$

(5, [0.53,1.70])

$C\left(k_{3},k_{5}\right)\left(u_{1}\right)$

(2, [0.73,1.26])

$C\left(k_{5},k_{7}\right)\left(u_{4}\right)$

(3, [0.60,1.23])

$C\left(k_{3},k_{5}\right)\left(u_{2}\right)$

(4, [0.70,1.46])

$C\left(k_{5},k_{7}\right)\left(u_{5}\right)$

(2, [0.46,0.86])

$C\left(k_{3},k_{5}\right)\left(u_{3}\right)$

(1, [0.73,1.13])

$C\left(k_{5},k_{7}\right)\left(u_{6}\right)$

(6, [1.00,1.46])

*Now we will evaluate the Score* $S\left(k_{s^{\prime }},l_{t^{\prime }}\right)\left(u_{i}\right);\;\forall \;u_{i}\in U,\;\left(k_{s^{\prime }},l_{t^{\prime }}\right)\in \left(K\times L\right),\;\;s^{\prime }=1,3,5\;and\;t^{\prime }=4,5,7$ *by using step 4 of Algorithm 1.*


$S\left(k_{s^{\prime }},k_{t^{\prime }}\right)\left(u_{i}\right)$


***The Score***


$S\left(k_{s^{\prime }},k_{t^{\prime }}\right)\left(u_{i}\right)$


***The Score***


$S\left(k_{1},k_{4}\right)\left(u_{1}\right)$

15.51

$S\left(k_{3},k_{5}\right)\left(u_{4}\right)$

4.2

$S\left(k_{1},k_{4}\right)\left(u_{2}\right)$

75.11

$S\left(k_{3},k_{5}\right)\left(u_{5}\right)$

104

$S\left(k_{1},k_{4}\right)\left(u_{3}\right)$

−97.72

$S\left(k_{3},k_{5}\right)\left(u_{6}\right)$

−92.5

$S\left(k_{1},k_{4}\right)\left(u_{4}\right)$

−131.46

$S\left(k_{3},k_{7}\right)\left(u_{1}\right)$

74.76

$S\left(k_{1},k_{4}\right)\left(u_{5}\right)$

96.9

$S\left(k_{3},k_{7}\right)\left(u_{2}\right)$

73.2

$S\left(k_{1},k_{4}\right)\left(u_{6}\right)$

24.92

$S\left(k_{3},k_{7}\right)\left(u_{3}\right)$

52.8

$S\left(k_{1},k_{5}\right)\left(u_{1}\right)$

−12.16

$S\left(k_{3},k_{7}\right)\left(u_{4}\right)$

−72.8

$S\left(k_{1},k_{5}\right)\left(u_{2}\right)$

4.2

$S\left(k_{3},k_{7}\right)\left(u_{5}\right)$

−32.94

$S\left(k_{1},k_{5}\right)\left(u_{3}\right)$

0.34

$S\left(k_{3},k_{7}\right)\left(u_{6}\right)$

−74

$S\left(k_{1},k_{5}\right)\left(u_{4}\right)$

−30

$S\left(k_{5},k_{4}\right)\left(u_{1}\right)$

−53.13

$S\left(k_{1},k_{5}\right)\left(u_{5}\right)$

71

$S\left(k_{5},k_{4}\right)\left(u_{2}\right)$

44.77

$S\left(k_{1},k_{5}\right)\left(u_{6}\right)$

−29.82

$S\left(k_{5},k_{4}\right)\left(u_{3}\right)$

32.2

$S\left(k_{1},k_{7}\right)\left(u_{1}\right)$

112.98

$S\left(k_{5},k_{4}\right)\left(u_{4}\right)$

−60.06

$S\left(k_{1},k_{7}\right)\left(u_{2}\right)$

68.1

$S\left(k_{5},k_{4}\right)\left(u_{5}\right)$

−32.1

$S\left(k_{1},k_{7}\right)\left(u_{3}\right)$

−115.17

$S\left(k_{5},k_{4}\right)\left(u_{6}\right)$

47.25

$S\left(k_{1},k_{7}\right)\left(u_{4}\right)$

−71.63

$S\left(k_{5},k_{5}\right)\left(u_{1}\right)$

−42

$S\left(k_{1},k_{7}\right)\left(u_{5}\right)$

−27.3

$S\left(k_{5},k_{5}\right)\left(u_{2}\right)$

2.64

$S\left(k_{1},k_{7}\right)\left(u_{6}\right)$

70.67

$S\left(k_{5},k_{5}\right)\left(u_{3}\right)$

−26.46

$S\left(k_{3},k_{4}\right)\left(u_{1}\right)$

−9.24

$S\left(k_{5},k_{5}\right)\left(u_{4}\right)$

88.2

$S\left(k_{3},k_{4}\right)\left(u_{2}\right)$

91.76

$S\left(k_{5},k_{5}\right)\left(u_{5}\right)$

−6

$S\left(k_{3},k_{4}\right)\left(u_{3}\right)$

31.9

$S\left(k_{5},k_{5}\right)\left(u_{6}\right)$

2.64

$S\left(k_{3},k_{4}\right)\left(u_{4}\right)$

−137.76

$S\left(k_{5},k_{7}\right)\left(u_{1}\right)$

−4.62

$S\left(k_{3},k_{4}\right)\left(u_{5}\right)$

81.6

$S\left(k_{5},k_{7}\right)\left(u_{2}\right)$

38.1

$S\left(k_{3},k_{4}\right)\left(u_{6}\right)$

−82.2

$S\left(k_{5},k_{7}\right)\left(u_{3}\right)$

43.89

$S\left(k_{3},k_{5}\right)\left(u_{1}\right)$

−5.88

$S\left(k_{5},k_{7}\right)\left(u_{4}\right)$

−29.96

$S\left(k_{3},k_{5}\right)\left(u_{2}\right)$

18.5

$S\left(k_{5},k_{7}\right)\left(u_{5}\right)$

−111.51

$S\left(k_{3},k_{5}\right)\left(u_{3}\right)$

−21.2

$S\left(k_{5},k_{7}\right)\left(u_{6}\right)$

100.27

*By using step 5 of Algorithm 1 the weighted values for each* $S(u_{l})$ *is:*

$$S\left(u_{1}\right)=\;\;76.22,\;\;\;S\left(u_{2}\right)=416.38,\;S\left(u_{3}\right)=-99.42,$$

$$S\left(u_{4}\right)=-441.27,\;S\left(u_{5}\right)=\;\;143.65,\;S\left(u_{6}\right)=-32.77.$$

*Here the optimal decision by using step 6 of Algorithm 1 is:*

$$X=arg\;max\;\{S\left(u_{1}\right),S\left(u_{2}\right),S\left(u_{3}\right),S\left(u_{4}\right),S\left(u_{5}\right),S\left(u_{6}\right)\}.$$

*Thus,* $u_{2}$ *is the best choice.*

*Hence Mr.*X will select $u_{2}$ person as a personal secretary.

**Figure 6:**
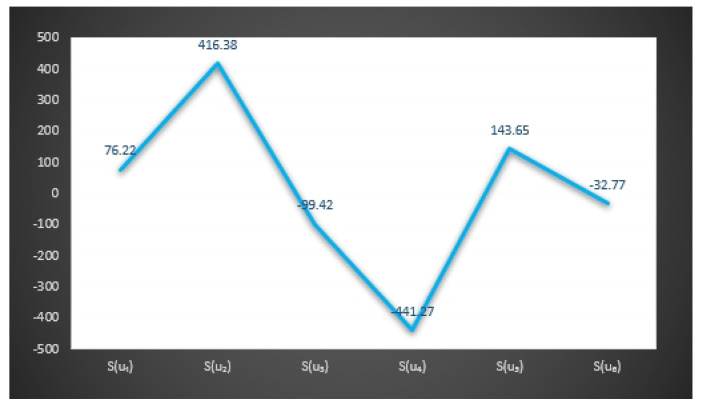
Graphical behavior of score values of Example 13 by means of Algorithm 1.

**Figure 7:**
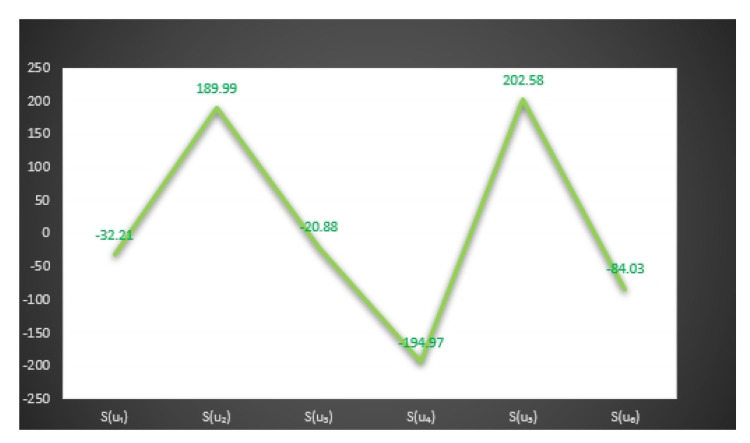
Graphical behavior of score values of Example 14 by means of Algorithm 2.

In Figure 6 and Figure 7, we give the graphical behavior of score values of Examples 13 and 14 by means of Algorithms 1 and 2 respectively.

**Example** **14.** *Consider the Example 13 and assume that Mr.* X *wants to select one such person depending on one of two parameters only. Suppose there are two observations* $\left(B_{b},K,5\right)$ *and* $\left(Y_{y},L,8\right)$ *by two experts as follows:*

*Firstly, we will evaluate the soft min-OR operation* $\left(Q_{q},K\times L,5\right)=\left(B_{b},K,5\right)\hat{\vee }\left(Y_{y},L,8\right)$ *by using step 2 of Algorithm 2:*

$$\begin{array}{c} \begin{array}{cc} (Q_{q},K\times L,5)=\{ & (\left(k_{1},k_{4}\right),(\frac{\left(u_{1},1\right)}{\left[0.36,0.76],[0.30,0.70\right]},\frac{\left(u_{2},3\right)}{\left[0.40,0.83],[0.43,0.70\right]},\frac{\left(u_{3},0\right)}{\left[0.23,0.36],[0.36,0.53\right]},\\ & \;\;\;\;\;\;\;\;\;\;\;\;\;\;\;\;\;\;\;\;\;\frac{\left(u_{4},2\right)}{\left[0.10,0.50],[0.40,0.53\right]},\frac{\left(u_{5},4\right)}{\left[0.46,0.66],[0.36,0.96\right]},\frac{\left(u_{6},2\right)}{\left[0.50,0.66],[0.26,0.73\right]})),\\ & (\left(k_{1},k_{5}\right),(\frac{\left(u_{1},1\right)}{\left[0.43,0.70],[0.50,0.63\right]},\frac{\left(u_{2},1\right)}{\left[0.26,0.90],[0.30,0.66\right]},\frac{\left(u_{3},0\right)}{\left[0.16,0.30],[0.23,0.46\right]},\\ & \;\;\;\;\;\;\;\;\;\;\;\;\;\;\;\;\;\;\;\;\;\frac{\left(u_{4},2\right)}{\left[0.36,0.63],[0.56,0.66\right]},\frac{\left(u_{5},4\right)}{\left[0.53,0.83],[0.56,0.86\right]},\frac{\left(u_{6},3\right)}{\left[0.50,0.63],[0.26,0.60\right]})),\\ & (\left(k_{1},k_{7}\right),(\frac{\left(u_{1},1\right)}{\left[0.70,0.86],[0.36,0.50\right]},\frac{\left(u_{2},3\right)}{\left[0.46,0.86],[0.36,0.70\right]},\frac{\left(u_{3},0\right)}{\left[0.10,0.70],[0.10,0.66\right]}, \end{array} \end{array}$$

$$\begin{array}{c} \begin{array}{cc} & \;\;\;\;\;\;\;\;\;\;\;\;\;\;\;\;\;\;\;\;\;\frac{\left(u_{4},2\right)}{\left[0.10,0.43],[0.40,0.66\right]},\frac{\left(u_{5},2\right)}{\left[0.33,0.56],[0.36,0.76\right]},\frac{\left(u_{6},3\right)}{\left[0.53,0.70],[0.43,0.60\right]})),\\ & (\left(k_{3},k_{4}\right),(\frac{\left(u_{1},0\right)}{\left[0.30,0.83],[0.30,0.73\right]},\frac{\left(u_{2},4\right)}{\left[0.46,0.70],[0.50,0.83\right]},\frac{\left(u_{3},1\right)}{\left[0.63,0.73],[0.46,0.80\right]},\\ & \;\;\;\;\;\;\;\;\;\;\;\;\;\;\;\;\;\;\;\;\;\frac{\left(u_{4},3\right)}{\left[0.23,0.56],[0.33,0.53\right]},\frac{\left(u_{5},2\right)}{\left[0.70,0.80],[0.36,0.90\right]},\frac{\left(u_{6},2\right)}{\left[0.26,0.53],[0.26,0.70\right]})),\\ & (\left(k_{3},k_{5}\right),(\frac{\left(u_{1},0\right)}{\left[0.36,0.76],[0.50,0.66\right]},\frac{\left(u_{2},1\right)}{\left[0.36,0.83],[0.43,0.80\right]},\frac{\left(u_{3},0\right)}{\left[0.60,0.70],[0.36,0.76\right]},\\ & \;\;\;\;\;\;\;\;\;\;\;\;\;\;\;\;\;\;\;\;\;\frac{\left(u_{4},3\right)}{\left[0.43,0.70],[0.53,0.66\right]},\frac{\left(u_{5},2\right)}{\left[0.73,0.90],[0.56,0.76\right]},\frac{\left(u_{6},3\right)}{\left[0.26,0.46],[0.26,0.56\right]})),\\ & (\left(k_{3},k_{7}\right),(\frac{\left(u_{1},0\right)}{\left[0.66,0.90],[0.36,0.56\right]},\frac{\left(u_{2},4\right)}{\left[0.53,0.76],[0.46,0.83\right]},\frac{\left(u_{3},1\right)}{\left[0.56,0.90],[0.30,0.86\right]},\\ & \;\;\;\;\;\;\;\;\;\;\;\;\;\;\;\;\;\;\;\;\;\frac{\left(u_{4},3\right)}{\left[0.23,0.50],[0.33,0.66\right]},\frac{\left(u_{5},2\right)}{\left[0.60,0.70],[0.36,0.63\right]},\frac{\left(u_{6},4\right)}{\left[0.33,0.60],[0.43,0.56\right]})),\\ & (\left(k_{5},k_{4}\right),(\frac{\left(u_{1},4\right)}{\left[0.23,0.70],[0.26,0.70\right]},\frac{\left(u_{2},3\right)}{\left[0.43,0.76],[0.40,0.83\right]},\frac{\left(u_{3},2\right)}{\left[0.56,0.73],[0.50,0.73\right]},\\ & \;\;\;\;\;\;\;\;\;\;\;\;\;\;\;\;\;\;\;\;\;\frac{\left(u_{4},3\right)}{\left[0.50,0.73],[0.40,0.53\right]},\frac{\left(u_{5},0\right)}{\left[0.36,0.66],[0.36,0.80\right]},\frac{\left(u_{6},1\right)}{\left[0.56,0.86],[0.40,0.80\right]})),\\ & (\left(k_{5},k_{5}\right),(\frac{\left(u_{1},2\right)}{\left[0.30,0.63],[0.46,0.63\right]},\frac{\left(u_{2},1\right)}{\left[0.30,0.86],[0.26,0.80\right]},\frac{\left(u_{3},0\right)}{\left[0.53,0.70],[0.43,0.70\right]},\\ & \;\;\;\;\;\;\;\;\;\;\;\;\;\;\;\;\;\;\;\;\;\frac{\left(u_{4},3\right)}{\left[0.63,0.80],[0.56,0.66\right]},\frac{\left(u_{5},0\right)}{\left[0.43,0.83],[0.56,0.66\right]},\frac{\left(u_{6},1\right)}{\left[0.56,0.83],[0.40,0.73\right]})),\\ & (\left(k_{5},k_{7}\right),(\frac{\left(u_{1},4\right)}{\left[0.63,0.83],[0.33,0.50\right]},\frac{\left(u_{2},3\right)}{\left[0.50,0.80],[0.33,0.83\right]},\frac{\left(u_{3},2\right)}{\left[0.50,0.90],[0.36,0.80\right]},\\ & \;\;\;\;\;\;\;\;\;\;\;\;\;\;\;\;\;\;\;\;\;\frac{\left(u_{4},3\right)}{\left[0.50,0.70],[0.40,0.66\right]},\frac{\left(u_{5},0\right)}{\left[0.16,0.56],[0.36,0.46\right]},\frac{\left(u_{6},1\right)}{\left[0.60,0.90],[0.50,0.73\right]}))\}. \end{array} \end{array}$$

*Then we will evaluate the choice value* $C\left(k_{s^{\prime }},l_{t^{\prime }}\right)\left(u_{i}\right);\;\forall \;u_{i}\in U,\;\left(k_{s^{\prime }},l_{t^{\prime }}\right)\in \left(K\times L\right),\;\;s^{\prime }=1,3,5\;and\;t^{\prime }=4,5,7$ *by using step 3 of Algorithm 2:*


$C\left(k_{s^{\prime }},k_{t^{\prime }}\right)\left(u_{i}\right)$


***(Grading, Interval Value)***


$C\left(k_{s^{\prime }},k_{t^{\prime }}\right)\left(u_{i}\right)$


***(Grading, Interval Value)***


$C\left(k_{1},k_{4}\right)\left(u_{1}\right)$

(1, [0.66,1.46])

$C\left(k_{3},k_{5}\right)\left(u_{4}\right)$

(3, [0.96,1.36])

$C\left(k_{1},k_{4}\right)\left(u_{2}\right)$

(3, [0.83,1.53])

$C\left(k_{3},k_{5}\right)\left(u_{5}\right)$

(2, [1.30,1.66])

$C\left(k_{1},k_{4}\right)\left(u_{3}\right)$

(0, [0.60,0.90])

$C\left(k_{3},k_{5}\right)\left(u_{6}\right)$

(3, [0.53,1.03])

$C\left(k_{1},k_{4}\right)\left(u_{4}\right)$

(2, [0.50,1.03])

$C\left(k_{3},k_{7}\right)\left(u_{1}\right)$

(0, [1.03,1.46])

$C\left(k_{1},k_{4}\right)\left(u_{5}\right)$

(4, [0.83,1.63])

$C\left(k_{3},k_{7}\right)\left(u_{2}\right)$

(4, [1.00,1.60])

$C\left(k_{1},k_{4}\right)\left(u_{6}\right)$

(2, [0.76,1.40])

$C\left(k_{3},k_{7}\right)\left(u_{3}\right)$

(1, [0.86,1.76])

$C\left(k_{1},k_{5}\right)\left(u_{1}\right)$

(1, [0.93,1.33])

$C\left(k_{3},k_{7}\right)\left(u_{4}\right)$

(3, [0.56,1.16])

$C\left(k_{1},k_{5}\right)\left(u_{2}\right)$

(1, [0.56,1.56])

$C\left(k_{3},k_{7}\right)\left(u_{5}\right)$

(2, [0.96,1.33])

$C\left(k_{1},k_{5}\right)\left(u_{3}\right)$

(0, [0.40,0.76])

$C\left(k_{3},k_{7}\right)\left(u_{6}\right)$

(4, [0.76,1.16])

$C\left(k_{1},k_{5}\right)\left(u_{4}\right)$

(2, [0.93,1.30])

$C\left(k_{5},k_{4}\right)\left(u_{1}\right)$

(4, [0.50,1.40])

$C\left(k_{1},k_{5}\right)\left(u_{5}\right)$

(4, [1.10,1.70])

$C\left(k_{5},k_{4}\right)\left(u_{2}\right)$

(3, [0.83,1.60])

$C\left(k_{1},k_{5}\right)\left(u_{6}\right)$

(3, [0.76,1.23])

$C\left(k_{5},k_{4}\right)\left(u_{3}\right)$

(2, [1.06,1.46])

$C\left(k_{1},k_{7}\right)\left(u_{1}\right)$

(1, [1.06,1.36])

$C\left(k_{5},k_{4}\right)\left(u_{4}\right)$

(3, [0.90,1.26])

$C\left(k_{1},k_{7}\right)\left(u_{2}\right)$

(3, [0.83,1.56])

$C\left(k_{5},k_{4}\right)\left(u_{5}\right)$

(0, [0.73,1.46])

$C\left(k_{1},k_{7}\right)\left(u_{3}\right)$

(0, [0.20,1.36])

$C\left(k_{5},k_{4}\right)\left(u_{6}\right)$

(1, [0.96,1.66])

$C\left(k_{1},k_{7}\right)\left(u_{4}\right)$

(2, [0.50,1.10])

$C\left(k_{5},k_{5}\right)\left(u_{1}\right)$

(2, [0.76,1.26])

$C\left(k_{1},k_{7}\right)\left(u_{5}\right)$

(2, [0.70,1.33])

$C\left(k_{5},k_{5}\right)\left(u_{2}\right)$

(1, [0.56,1.66])

$C\left(k_{1},k_{7}\right)\left(u_{6}\right)$

(3, [0.96,1.30])

$C\left(k_{5},k_{5}\right)\left(u_{3}\right)$

(0, [0.96,1.40])

$C\left(k_{3},k_{4}\right)\left(u_{1}\right)$

(0, [0.60,1.56])

$C\left(k_{5},k_{5}\right)\left(u_{4}\right)$

(3, [1.20,1.46])

$C\left(k_{3},k_{4}\right)\left(u_{2}\right)$

(4, [0.96,1.53])

$C\left(k_{5},k_{5}\right)\left(u_{5}\right)$

(0, [1.00,1.50])

$C\left(k_{3},k_{4}\right)\left(u_{3}\right)$

(1, [1.10,1.53])

$C\left(k_{5},k_{5}\right)\left(u_{6}\right)$

(1, [0.96,1.56])

$C\left(k_{3},k_{4}\right)\left(u_{4}\right)$

(3, [0.56,1.10])

$C\left(k_{5},k_{7}\right)\left(u_{1}\right)$

(4, [0.96,1.33])

$C\left(k_{3},k_{4}\right)\left(u_{5}\right)$

(2, [1.06,1.70])

$C\left(k_{5},k_{7}\right)\left(u_{2}\right)$

(3, [0.83,1.63])

$C\left(k_{3},k_{4}\right)\left(u_{6}\right)$

(2, [0.53,1.23])

$C\left(k_{5},k_{7}\right)\left(u_{3}\right)$

(2, [0.86,1.70])

$C\left(k_{3},k_{5}\right)\left(u_{1}\right)$

(0, [0.86,1.43])

$C\left(k_{5},k_{7}\right)\left(u_{4}\right)$

(3, [0.90,1.36])

$C\left(k_{3},k_{5}\right)\left(u_{2}\right)$

(1, [0.80,1.63])

$C\left(k_{5},k_{7}\right)\left(u_{5}\right)$

(0, [0.53,1.03])

$C\left(k_{3},k_{5}\right)\left(u_{3}\right)$

(0, [0.96,1.46])

$C\left(k_{5},k_{7}\right)\left(u_{6}\right)$

(1, [1.10,1.63])

*Now we will evaluate the Score* $S\left(k_{s^{\prime }},l_{t^{\prime }}\right)\left(u_{i}\right);\;\forall \;u_{i}\in U,\;\left(k_{s^{\prime }},l_{t^{\prime }}\right)\in \left(K\times L\right),\;\;s^{\prime }=1,3,5\;and\;t^{\prime }=4,5,7$ *by using step 4 of Algorithm 2.*


$S\left(k_{s^{\prime }},k_{t^{\prime }}\right)\left(u_{i}\right)$


***The Score***


$S\left(k_{s^{\prime }},k_{t^{\prime }}\right)\left(u_{i}\right)$


***The Score***


$S\left(k_{1},k_{4}\right)\left(u_{1}\right)$

7.67

$S\left(k_{3},k_{5}\right)\left(u_{4}\right)$

−1.08

$S\left(k_{1},k_{4}\right)\left(u_{2}\right)$

38.57

$S\left(k_{3},k_{5}\right)\left(u_{5}\right)$

52.92

$S\left(k_{1},k_{4}\right)\left(u_{3}\right)$

−37.56

$S\left(k_{3},k_{5}\right)\left(u_{6}\right)$

−83.16

$S\left(k_{1},k_{4}\right)\left(u_{4}\right)$

−44.25

$S\left(k_{3},k_{7}\right)\left(u_{1}\right)$

18.2

$S\left(k_{1},k_{4}\right)\left(u_{5}\right)$

63.12

$S\left(k_{3},k_{7}\right)\left(u_{2}\right)$

47.04

$S\left(k_{1},k_{4}\right)\left(u_{6}\right)$

12.45

$S\left(k_{3},k_{7}\right)\left(u_{3}\right)$

31.2

$S\left(k_{1},k_{5}\right)\left(u_{1}\right)$

12

$S\left(k_{3},k_{7}\right)\left(u_{4}\right)$

−66.4

$S\left(k_{1},k_{5}\right)\left(u_{2}\right)$

1.92

$S\left(k_{3},k_{7}\right)\left(u_{5}\right)$

1.7

$S\left(k_{1},k_{5}\right)\left(u_{3}\right)$

−61.6

$S\left(k_{3},k_{7}\right)\left(u_{6}\right)$

−50.88

$S\left(k_{1},k_{5}\right)\left(u_{4}\right)$

12.3

$S\left(k_{5},k_{4}\right)\left(u_{1}\right)$

−58.08

$S\left(k_{1},k_{5}\right)\left(u_{5}\right)$

101.76

$S\left(k_{5},k_{4}\right)\left(u_{2}\right)$

14.44

$S\left(k_{1},k_{5}\right)\left(u_{6}\right)$

−11.78

$S\left(k_{5},k_{4}\right)\left(u_{3}\right)$

20.8

$S\left(k_{1},k_{7}\right)\left(u_{1}\right)$

27.12

$S\left(k_{5},k_{4}\right)\left(u_{4}\right)$

−16.34

$S\left(k_{1},k_{7}\right)\left(u_{2}\right)$

37.44

$S\left(k_{5},k_{4}\right)\left(u_{5}\right)$

−8.84

$S\left(k_{1},k_{7}\right)\left(u_{3}\right)$

−31.9

$S\left(k_{5},k_{4}\right)\left(u_{6}\right)$

26.6

$S\left(k_{1},k_{7}\right)\left(u_{4}\right)$

−37.24

$S\left(k_{5},k_{5}\right)\left(u_{1}\right)$

−28.08

$S\left(k_{1},k_{7}\right)\left(u_{5}\right)$

−1.12

$S\left(k_{5},k_{5}\right)\left(u_{2}\right)$

−8.64

$S\left(k_{1},k_{7}\right)\left(u_{6}\right)$

23.4

$S\left(k_{5},k_{5}\right)\left(u_{3}\right)$

−0.84

$S\left(k_{3},k_{4}\right)\left(u_{1}\right)$

−6

$S\left(k_{5},k_{5}\right)\left(u_{4}\right)$

30.24

$S\left(k_{3},k_{4}\right)\left(u_{2}\right)$

35.52

$S\left(k_{5},k_{5}\right)\left(u_{5}\right)$

5.04

$S\left(k_{3},k_{4}\right)\left(u_{3}\right)$

30.16

$S\left(k_{5},k_{5}\right)\left(u_{6}\right)$

7.56

$S\left(k_{3},k_{4}\right)\left(u_{4}\right)$

−66.5

$S\left(k_{5},k_{7}\right)\left(u_{1}\right)$

−2.88

$S\left(k_{3},k_{4}\right)\left(u_{5}\right)$

46.5

$S\left(k_{5},k_{7}\right)\left(u_{2}\right)$

17.1

$S\left(k_{3},k_{4}\right)\left(u_{6}\right)$

−43.5

$S\left(k_{5},k_{7}\right)\left(u_{3}\right)$

24

$S\left(k_{3},k_{5}\right)\left(u_{1}\right)$

−2.16

$S\left(k_{5},k_{7}\right)\left(u_{4}\right)$

−5.7

$S\left(k_{3},k_{5}\right)\left(u_{2}\right)$

6.6

$S\left(k_{5},k_{7}\right)\left(u_{5}\right)$

−58.5

$S\left(k_{3},k_{5}\right)\left(u_{3}\right)$

4.86

$S\left(k_{5},k_{7}\right)\left(u_{6}\right)$

35.28

*By using step 5 of Algorithm 2 the weighted values for each* $S(u_{l})$ *is:*

$$S\left(u_{1}\right)=-32.21,\;S\left(u_{2}\right)=189.99,\;S\left(u_{3}\right)=-20.88,$$

$$S\left(u_{4}\right)=-194.97,\;S\left(u_{5}\right)=202.58,\;\;S\left(u_{6}\right)=-84.03.$$

*Here the optimal decision by using step 6 of Algorithm 2 is:*

$$X=arg\;max\;\{S\left(u_{1}\right),S\left(u_{2}\right),S\left(u_{3}\right),S\left(u_{4}\right),S\left(u_{5}\right),S\left(u_{6}\right)\}.$$

*Thus,* $u_{5}$ *is the best choice.*

*Hence Mr.*X*will select*$u_{5}$*person as a personal secretary*.

**Figure 8:**
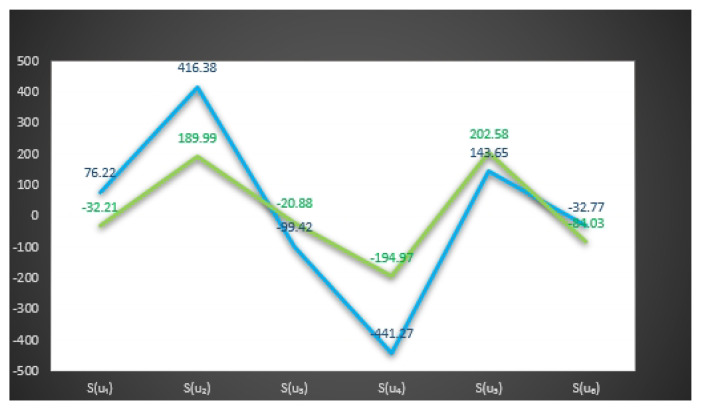
Comparison of score values of Examples 13 and 14 by means of Algorithms 2 and 2.

In Figure 8, we observe the following relations between the score values of Examples 13 and 14 by means of Algorithms 1 and 2, respectively.

$$S\left(u_{4}\right)⪯S\left(u_{3}\right)⪯S\left(u_{6}\right)⪯S\left(u_{1}\right)⪯S\left(u_{5}\right)⪯S\left(u_{2}\right),$$

$$S\left(u_{4}\right)⪯S\left(u_{6}\right)⪯S\left(u_{1}\right)⪯S\left(u_{3}\right)⪯S\left(u_{2}\right)⪯S\left(u_{5}\right).$$

## 9. Conclusions

The main intent of this work is to present a possibility belief interval-valued N-soft set by consolidating a belief interval value and possibility with N-soft set and its applications for solving the complicated decision-making problems in various fields of life. There are great applications for the belief interval in many different fields of life, while the other tool N-soft set theory is arising as a prosperous mathematical approach for manipulating ambivalent information. First, we defined basic theory and definitions of important sets in a very clear way. Then we discussed the BIVNSS, various algebraic operations, and their fundamental properties. Then we defined PBIVNSS, its algebraic operations, its elemental properties, and also its applications for decision-making problems. We have also provided algorithms for these decision-making methods and showed how decision-making methods are applied successfully in the problems of real life. In further work, this idea can be seen in many other algebraic expressions and topological structures.

## Data Availability

No data were used to support this study.
